# Mitochondria supply sub-lethal signals for cytokine secretion and DNA-damage in *H. pylori* infection

**DOI:** 10.1038/s41418-022-01009-9

**Published:** 2022-05-03

**Authors:** Benedikt Dörflinger, Mohamed Tarek Badr, Aladin Haimovici, Lena Fischer, Juliane Vier, Arlena Metz, Bianca Eisele, Peter Bronsert, Konrad Aumann, Jens Höppner, Collins Waguia Kontchou, Ishita Parui, Arnim Weber, Susanne Kirschnek, Georg Häcker

**Affiliations:** 1 Faculty of Medicine, Institute of Medical Microbiology and Hygiene, Medical Center, University of Freiburg, Freiburg, Germany; 2 Faculty of Medicine, Institute for Surgical Pathology, Medical Center, University of Freiburg, Freiburg, Germany; 3 Comprehensive Cancer Center Freiburg, Medical Center, University of Freiburg, Freiburg, Germany; 4 Center for Pathology Allgäu, Kempten Allgäu, Germany; 5 Department of Surgery, University Medical Center Schleswig-Holstein, Lübeck, Germany; 6 BIOSS Centre for Biological Signalling Studies, University of Freiburg, Freiburg, Germany

**Keywords:** Cell death and immune response, Infectious diseases, Acute inflammation, Infectious diseases, Cancer

## Abstract

The bacterium *Helicobacter pylori* induces gastric inflammation and predisposes to cancer. *H. pylori*-infected epithelial cells secrete cytokines and chemokines and undergo DNA-damage. We show that the host cell’s mitochondrial apoptosis system contributes to cytokine secretion and DNA-damage in the absence of cell death. *H. pylori* induced secretion of cytokines/chemokines from epithelial cells, dependent on the mitochondrial apoptosis machinery. A signalling step was identified in the release of mitochondrial Smac/DIABLO, which was required for alternative NF-κB-activation and contributed to chemokine secretion. The bacterial *cag*-pathogenicity island and bacterial muropeptide triggered mitochondrial host cell signals through the pattern recognition receptor NOD1. *H. pylori*-induced DNA-damage depended on mitochondrial apoptosis signals and the caspase-activated DNAse. In biopsies from *H. pylori*-positive patients, we observed a correlation of Smac-levels and inflammation. Non-apoptotic cells in these samples showed evidence of caspase-3-activation, correlating with phosphorylation of the DNA-damage response kinase ATM. Thus, *H. pylori* activates the mitochondrial apoptosis pathway to a sub-lethal level. During infection, Smac has a cytosolic, pro-inflammatory role in the absence of apoptosis. Further, DNA-damage through sub-lethal mitochondrial signals is likely to contribute to mutagenesis and cancer development.

## Introduction

*Helicobacter pylori* (*Hp*) colonizes the gastric mucus layer of approximately half of the world’s human population. In most cases *H. pylori* is transmitted in families in childhood and remains associated with its host for decades. An innate and adaptive immune response to the infection ensues, causing chronic gastritis. Infection is mostly asymptomatic but is a major risk factor for gastric and duodenal ulcers and gastric malignancies [1, 2]. *H. pylori* uses a type IV-secretion system, encoded on the 37 kb *cag*-pathogenicity island (*cag*-PAI), to interact closely with gastric epithelial cells and to deliver the cytotoxin associated antigen A (CagA) into the cells [3, 4]. Epithelial cells recognize *Hp* and activate a number of signalling pathways. Various pattern recognition receptors (PRR) have been implicated, and the cells secrete cytokines/chemokines [5]. This epithelial response likely contributes to the initiation of inflammation. *Hp* can induce DNA-damage, manifesting as an increased mutation rate [6, 7] but also double-strand breaks (DSBs) [8, 9]. DSBs depended on the type IV-secretion system and received a contribution from host cell factors [8, 10, 11]. DNA-damage induced by direct contact of *Hp* with gastric epithelial cells may introduce genomic mutations, contributing to cancer development.

Apoptotic cell death can contribute to protecting the replicative niche and to pathogen dissemination. Further, some pathogens can drive and inhibit more than one form of regulated cells death [12]; *Hp* has also been found to be able to induce pyroptosis in myeloid cells [13]. Many cases of apoptosis are orchestrated through the mitochondrial pathway. In this pathway, an apoptotic stimulus drives the release of the mitochondrial intermembrane space proteins cytochrome *c* and Smac into the cytosol, where they activate caspases. The intriguing feature of low-level activation of the apoptotic apparatus in the absence of cell death is a recent discovery [14, 15]. Mitochondrial release of cytochrome *c* does not have to be the point of no return. Rather, during mitochondrial pro-apoptotic signalling, small amounts of cytochrome *c* may be released, inducing little caspase activity. Small-scale mitochondrial outer membrane permeabilization (‘minority MOMP’ [16]) may however activate the caspase-activated DNAse (CAD), which can introduce persistent DNA-mutations [16, 17].

The apoptosis system often plays a role in infection, and many pathogens have pro- or anti-apoptotic effects, sometimes both [18]. We have recently reported that sub-lethal mitochondrial apoptosis signalling occurs during infection of epithelial cells with intracellular pathogens, from viruses to bacteria and a parasite [19]. Intriguingly, the mitochondrial apoptosis apparatus contributed to cytokine/chemokine secretion. The apoptosis apparatus can be very easily activated. Its low-level activation may therefore be a sensitive way to sense stimuli including infectious agents. High numbers of *Hp* in cell culture induce apoptosis [20, 21] but apoptosis is not a regular feature during *Hp*-infection of the stomach. We therefore hypothesized that ‘physiological’, lower numbers of *Hp*-infection activate the apoptosis pathway in epithelial cells to a sub-lethal level, which may contribute to the inflammatory response of the infected cells.

## Results

### Inactivation of mitochondrial apoptosis reduces the cytokine response during Hp-infection of epithelial cells

**Fig. 1:**
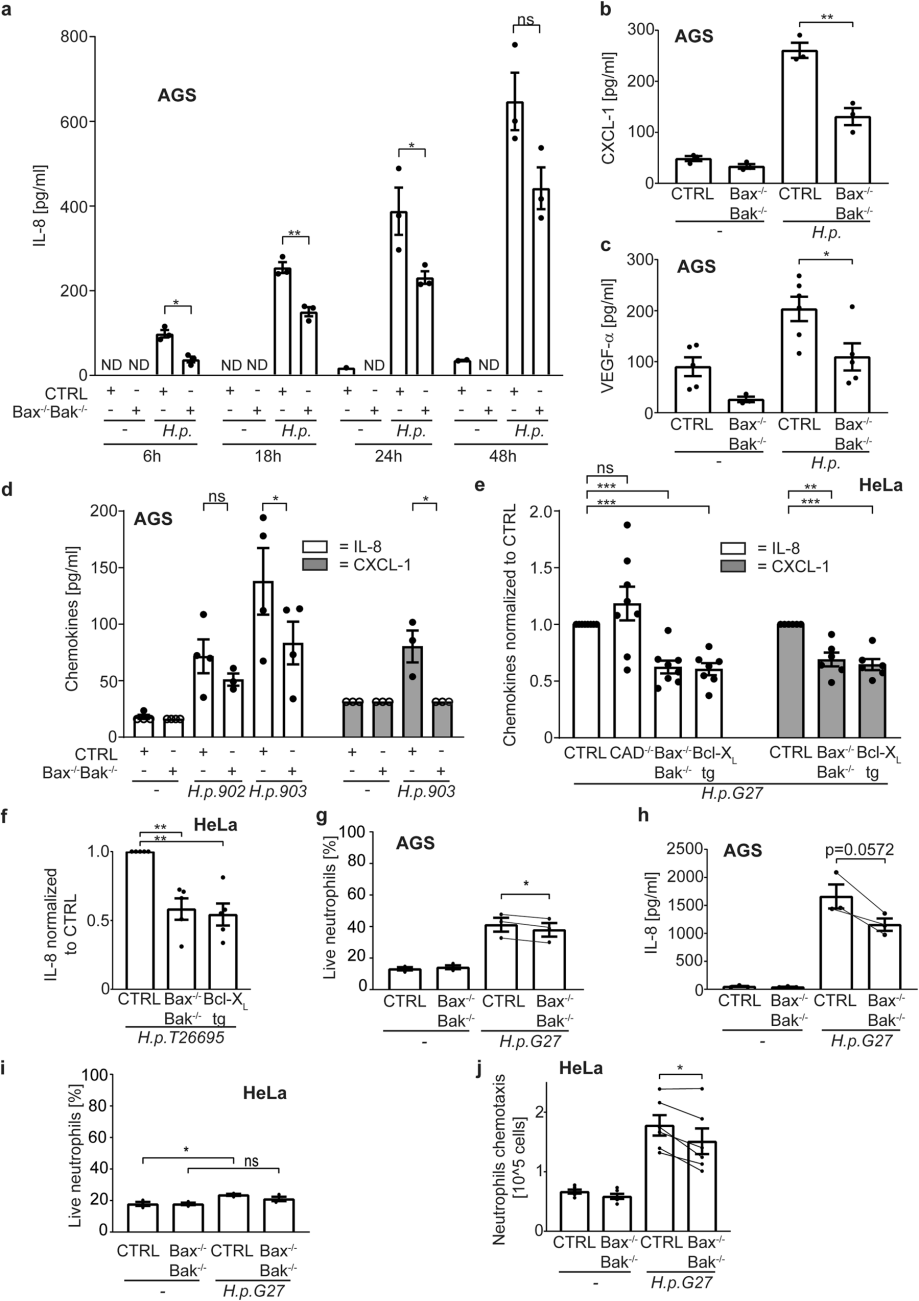
Cytokine/chemokine secretion is reduced in cells lacking mitochondrial apoptosis. **a** AGS cell lines were infected with *H. pylori* G27 strain with a multiplicity of infection (MOI) of 100 for different time periods. IL-8 in cell culture supernatant was measured by ELISA in three independent experiments. **b**, **c** AGS cell lines were infected with *H. pylori* G27 strain with a MOI of 100 for 18 h. CXCL-1 (**b**) and VEGF-α (**c**) were measured by a bead-based immunoassay and by ELISA for VEGF-α in three (**b**) or six (**c**) independent experiments. **d** AGS cell lines were infected with H. pylori clinical isolates 902, 903 at an MOI of 100 for 18 h. IL-8 and CXCL-1 were measured by ELISA in at least three independent experiments. The values of detection limit were used for statistical analyses if the measured chemokines were under detection limit. One data point was removed after Grubbs outlier testing. **e**, **f** HeLa cell lines were infected with *H. pylori* G27 (**e**) or T26695 (**f**) strains at an MOI of 100 for 24 h. IL-8 (**e**, **f**) and CXCL-1 (**e**) were measured by ELISA in eight (**e**), seven (**e**) and five (**f**) independent experiments. One data point was removed (**e**) after Grubbs outlier testing. All infected samples were normalized to CTRL infected. Not normalized data are shown in S8A-B. **g** AGS cell lines were infected with *H. pylori* G27 strain at an MOI of 100 for 18 h. The cell culture supernatant was passed through a 0.2 µM filter and co-incubated with human neutrophils for 24 h. Neutrophil survival was determined by AnnexinV/Live-Dead staining. (data are from three individual donors, with supernatants from three independent infection experiments tested for each donor). **h** AGS cell lines were infected with *H. pylori* G27 strain at an MOI of 100 for 18 h. The cell culture supernatant was passed through a 0.2 µM filter and co-incubated with human neutrophils derived from a healthy donor. Neutrophil supernatants were harvested after 24 h and assayed for IL-8 by ELISA. Neutrophil IL-8 secretion was calculated as the difference between total IL-8 amounts in supernatants after co-incubation and IL-8 amounts of AGS supernatants prior co-incubation; all secretion data are shown in Fig. S8C (data are from supernatants derived from three independent infection experiments). Lines connect data point from the same individual experiments. **I** HeLa cell lines were infected with *H. pylori* G27 strain at an MOI of 100 for 20 h. The cell culture supernatant was passed through a 0.2 µM filter and co-incubated with human neutrophils for 24 h. Neutrophil survival was determined by AnnexinV/PI staining. (data are from supernatants derived from three independent infection experiments). **j** HeLa cell lines were infected with *H. pylori* G27 strain at an MOI of 100 for 20 h. The cell culture supernatant was passed through a 0.2 µM filter. Human neutrophil chemotaxis was measured in a transwell migration assay (data are from six independent experiments). Lines demonstrate connected individual experiments. Data information: Bars represent the mean and dots the value of independent experiments. Hollow dots represent detection limit. Error bars show standard error of mean. Ns: *p* > 0.05, *, *p* < 0.05, **, *p* < 0.01, ***, *p* < 0.001. The significance were tested by parametric two-Way ANOVA with Dunnett’s post hoc testing (**a**, **d:** IL-8), parametric one-Way ANOVA with Sidak’s post hoc testing (**d:** CXCL-1), one sample *T*-Test (**e**, **f**), unpaired *T*-Test (**b**, **c**) and paired *T*-Test (**g**, **h**, **i**, **j**). CTRL, non-targeting control gRNA; Bax^−/−^Bak^−/−^, double knockout of Bax and Bak by CRISPR/Cas9; Bcl-X_L_ tg, overexpressing Bcl-X_L_; CAD^−/−^, depletion of CAD by CRISPR/Cas9.

Mitochondrial apoptosis occurs through the activity of Bcl-2-family members. Anti-apoptotic Bcl-2 proteins such as Bcl-X_L_ block apoptosis by binding to the pro-apoptotic members, including the trigger molecules (BH3-only proteins) and the two effectors, Bax and Bak [22]. To test for a potential contribution of the mitochondrial apoptosis system to *Hp*-induced inflammation, we first infected AGS gastric carcinoma cells with *Hp*. Infected cells secreted increasing amounts of IL-8 over time, and this was consistently reduced in Bax/Bak-deficient cells (Fig. 1a). In a screen for soluble products secreted by AGS cells upon *Hp*-infection we also detected CXCL1 and VEGFα (not shown; notably, the inflammasome products IL1β and IL-18 were not detected), and secretion of both was reduced from AGS cells lacking Bax and Bak (Fig. 1b, c; the confirmation of gene-modified cells made in this study is shown in Fig. S1). Chemokine-induction by a clinical *Hp*-isolate was also reduced (Fig. 1d), as was secretion from HeLa cells lacking Bax and Bak or overexpressing Bcl-X_L_ (Fig. 1e; for some experiments, the results were normalized. The original data from these experiments are all shown in Fig. S8). The same was seen for IL-8 with a second laboratory strain (Fig. 1f). IL-8-secretion from PMA-treated mutant cells was unaltered (Fig. S1o–q).

Supernatants from *Hp*-infected AGS cells increased neutrophil survival but to a smaller extent when supernatants were from Bax/Bak-deficient cells (Fig. 1g). Neutrophils secreted high amounts of IL-8, and this was reduced in supernatants from Bax/Bak-deficient AGS cells (Fig. 1h). Neutrophil survival in supernatants from *Hp*-infected HeLa cells showed the same pattern (Fig. 1i), and supernatants from Bax/Bak-deficient HeLa cells induced less neutrophil migration than supernatants from control cells (Fig. 1j). Thus, the mitochondrial apoptosis machinery contributes to chemokine secretion and inflammatory activity in *Hp*-infected epithelial cells.

### Hp-infection induces sub-lethal caspase-activation

**Fig. 2:**
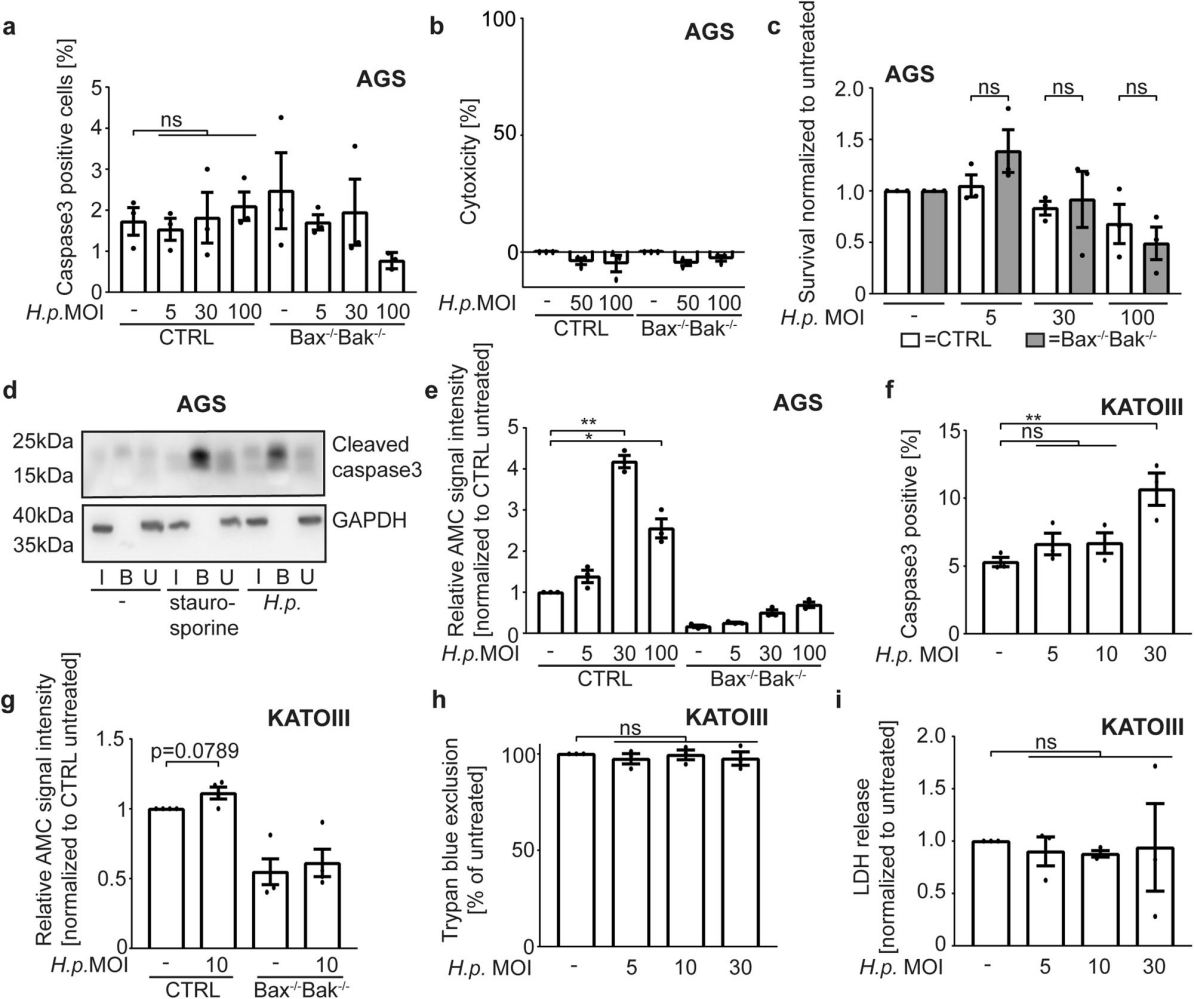
Detection of caspase-activation and cell death upon infection with Helicobacter pylori. **a** AGS cell lines were infected with different multiplicities of infection (MOI) of *H. pylori* G27 strain for 18 h. Active caspase3 was measured by flow cytometry using an antibody recognizing the cleavage product of caspase-3-activation in three independent experiments. **b** AGS cell lines were infected with different MOI of *H. pylori* G27 strain for 24 h. Epithelial cell survival was measured by LDH release assay in three independent experiments. Cytotoxicity was calculated as described in Methods. **c** AGS cell lines were infected with different MOI of *H. pylori* G27 strain. Cells were counted and 500 cells were seeded in triplicates in medium supplemented with antibiotics after 18 h of infection. Clonogenic growth was determined with crystal violet staining after one week. Data are from three independent experiments. Not normalized data are shown in S8D. **d** AGS cell lines were infected with *H. pylori* G27 strain at an MOI of 100 for 18 h. After 15 h, biotinylated VAD-fmk was added. Cells were lysed, and inhibitor-bound caspases were precipitated from cell lysates using neutravidin beads. Precipitated (i.e. inhibitor-bound, activated) caspase3 was detected by Western blotting. Shown is a representative Western blot from two independent experiments. **e** AGS cell lines were infected with various MOI of *H. pylori* G27 strain for 18 h. Effector caspase activity was measured in cell lysates with an AC-DEVD-AMC reporter substrate. Increasing fluorescence indicates effector caspase activity. Data are from three independent experiments. Not normalized data are shown in S8E. **f** KATOIII wild type cell lines were infected with different MOI of *H. pylori* G27 strain for 18 h. Active caspase3 was measured by flow cytometry using an antibody recognizing the cleavage product of caspase-3-activation (data are from three independent experiments). **g** KATOIII cell lines were infected with *H. pylori* G27 strain at an MOI of 10 for 5 h. Effector caspase activity was measured in cell lysates with an AC-DEVD-AMC reporter substrate. Increasing fluorescence was detected then effector caspases were active (data are from four independent experiments). Not normalized data are shown in S8F. **h** KATOIII wild type cell lines were infected with different MOI of *H. pylori* G27 strain for 18 h. Cell survival was measured by trypan blue exclusion in three independent experiments. Not normalized data are shown in S8G. **i** KATOIII wild type cell lines were infected with different MOI of *H. pylori* G27 strain for 18 h. Epithelial cell survival was measured by LDH release assay in three independent experiments. Not normalized data are shown in S8H. Data information: Bars represent the mean and dots the value of independent experiments. Error bars show standard error of mean. Ns: *p* > 0.05, *, *p* < 0.05, **, *p* < 0.01. Significance was tested by one sample *T*-Test (**g**, **h**, **i**) parametric (**a**, **f:** Dunnett´s post hoc test**; c:** Sidak ´s post hoc test) and non-parametric (**e:** Dunn´s post hoc test) one-Way ANOVA. CTRL, non-targeting control gRNA; Bax^−/−^Bak^−/−^, double knockout of Bax and Bak by CRISPR/Cas9. I, input; B, bound to beads; U, unbound.

High MOI of *Hp* have been reported to activate caspase-3 [21]. Effector caspase activity during sub-lethal signalling may be too low to be detectable by standard assays [16, 19]. No caspase-3 activation was observed by flow cytometry in *Hp*-infected AGS or KATOIII cells (Fig. 2a, S2A). No cytotoxicity (LDH-release and Trypan blue uptake) was seen up to 24 h (Fig. 2b, Fig. S2B). Infection up to MOI = 30 did not reduce long-term colony formation in AGS cells. At MOI = 100, there was some reduction, possibly because of the effect of *Hp* on the cytoskeleton [4]. This was however Bax/Bak independent (Fig. 2c) and therefore independent of mitochondrial apoptosis.

Biotinylated caspase-inhibitory peptide (bio-VAD) was however able to precipitate active caspase-3 from lysates of infected AGS cells (Fig. 2d; staurosporine was used as a positive control). Fluorigenic enzyme assay further showed a moderate but significant activation of effector caspases in lysates from *Hp*-infected AGS cells (Fig. 2e), which was blocked by the caspase-inhibitor Q-VD-OPh (Fig. S2B). A second gastric cell line, KATOIII, was more sensitive to *Hp*-induced apoptosis: caspase-3-positive cells were detectable upon infection at an MOI of 30 (Fig. 2f), with a trend to small amounts of DEVD-cleaving activity at MOI = 10 (Fig. 2g), where no caspase-3-positive cells were detected (Fig. 2f) and other measurements of cell death were negative (Fig. 2h, i). In *Hp* -infected HeLa cells, no active effector caspase was detectable using a reporter line [19] (Fig. S2A, C), and no signal was obtained by enzyme assay (Fig. S2D). No active effector caspases were precipitated from these cells (Fig. S2E), and no cytotoxicity was observed (Fig. S2F). A trend to a higher number of Hela cells with reduced mitochondrial membrane potential was seen (Fig. S2G). A time course over 48 h of infection of AGS control and Bax/Bak-deficient cells with *Hp* and the read-outs of Trypan blue uptake, LDH-release and DEVD-cleaving activity is shown in Fig. S3.

Low-level, sub-lethal caspase-activation can thus be measured in *Hp*-infected AGS and KATOIII but not HeLa cells in conditions where the mitochondrial apoptosis apparatus contributed to cytokine secretion. Thus, *Hp*-infection can generate a sub-lethal signal in the apoptosis pathway.

### Hp-infection induces release of Smac

Mitochondrial cytochrome *c*-release is required for caspase-activation, so small amounts of cytochrome *c* were likely to be released in the conditions here. Smac is released during (full) apoptosis concomitant with cytochrome *c*-release [23]; it therefore seemed possible that it is also released upon the *Hp*-dependent, sub-lethal signals. Smac is a mitochondrial protein but all known molecular functions of Smac are in the cytosol. Cytosolic Smac binds the X-linked inhibitor of apoptosis protein (XIAP), releasing its caspase-inhibitory function. Cytosolic Smac further inactivates cIAP1/2 [24–26]. Small-molecule Smac-mimetics have pro-inflammatory activity, primarily through the activation of alternative NF-κB: inactivation of cIAP1/2 by Smac and Smac-mimetics increases the levels of NIK and triggers alternative NF-κB, detectable as its active form, p52 [26, 27].

**Fig. 3:**
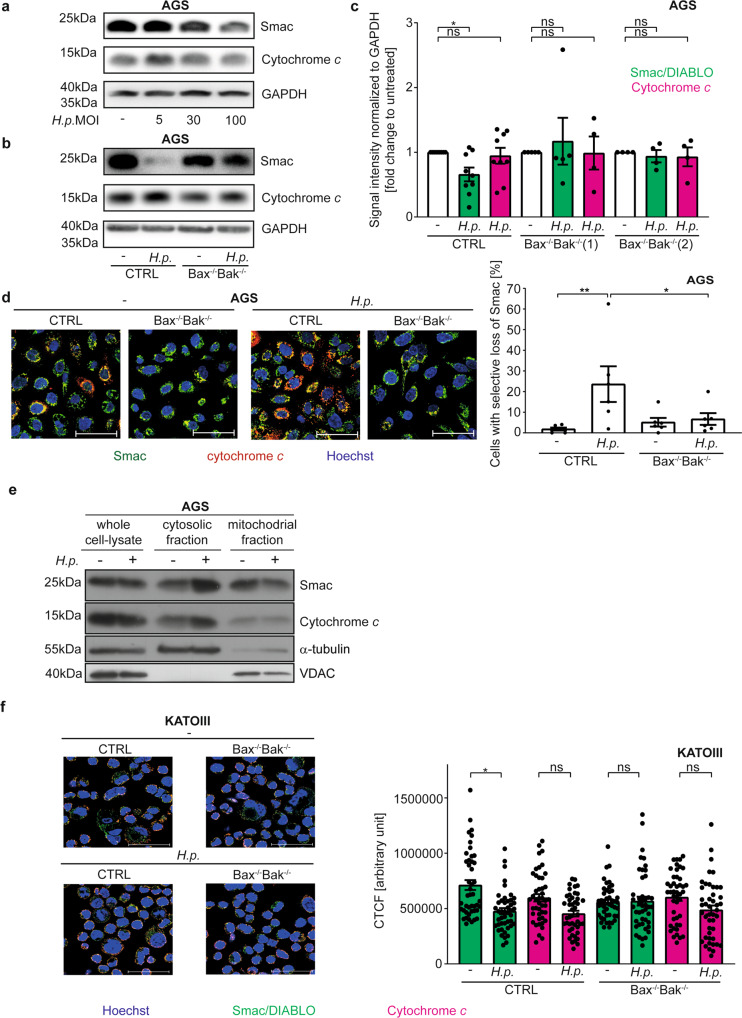
H. pylori induces Bax/Bak-dependent Smac-release. **a**–**c** AGS cell lines were infected with *H. pylori* G27 strain at an MOI of 100 for 18 h. Endogenous levels of Smac and cytochrome *c* from whole cell lysates were detected by Western blotting. Shown are representative Western blots from at least three (**a**: CTRL) or four (**b**: Bax^−/−^Bak^−/−^, clone 2) independent experiments and a quantification of all blots (**c**). Not normalized data are shown in S8I. **d** AGS cell lines (Bax^−/−^Bak^−/−^, clone 1) were infected with *H. pylori* G27 strain at an MOI of 100 for 18 h. Endogenous Smac (green) and cytochrome *c* (red) were detected by immunofluorescence. Shown are representative pictures and quantification of five independent experiments with at least 340 cells/condition. Scale bars indicate 50 µM. A larger magnification and individual fluorescence channels are shown in Fig. S4A. **e** AGS cells (vector control (CTRL)) infected with Helicobacter (G27, MOI 100 for 18 h) were separated into cytoplasmic and mitochondrial fractions for analysis of Smac- und cytochrome *c*-release. Fractionation was confirmed by Western blot analysis. α-tubulin was used as a cytoplasmic marker protein and VDAC as a marker of mitochondria. The immunoblots are representative of three independent experiments. **f** KATOIII cell lines were infected with *H. pylori* G27 strain at an MOI of 10 for 5 h. Endogenous Smac (green) and cytochrome *c* (red) were detected by immunofluorescence. Shown are representative pictures of three independent experiments. Bar, graph, Smac (green bars) and cytochrome *c* (red bars) fluorescence intensity were quantified by calculating the corrected total cell fluorescence (CTCF) in the same cells. Shown are the values of the individual cells of three independent experiments. Significance was calculated with the mean value of each individual experiment. A larger magnification and individual fluorescence channels are shown in Fig. S4C. Data information: Bars represent the mean and dots the value of independent experiments (**c**, **d**) or single cells (**f**). Error bars show standard error of mean. Ns: *p* > 0.05, *, *p* < 0.05, **, *p* < 0.01. The significance were tested by parametric (**d**, **f**: Sidak´s post hoc test) and non-parametric (**c**: Dunn´s post hoc test) one-way ANOVA. CTRL, non-targeting control gRNA; Bax^−/−^Bak^−/−^, double deletion of Bax and Bak by CRISPR/Cas9.

We hypothesized that Smac is released during *Hp* -infection, and a physiological function of Smac may be NF-κB-activation in pathogen-recognition. Increasing doses of *Hp* caused progressive loss of Smac but not cytochrome *c* from wt but not Bax/Bak-deficient AGS cells (Fig. 3a–c). By immunostaining, mitochondria of *Hp*-infected wt but not Bax/Bak-deficient cells showed cytochrome *c* but little Smac-fluorescence, indicating preferential loss of mitochondrial Smac (Fig. 3d, S4A). We further measured the loss of Smac and cytochrome *c* in AGS cells single-Bax or -Bak-deficient by microscopy. As shown in Fig. S4B, there was a trend towards a loss of both Smac and cytochrome *c* in wt cells and in cells lacking Bax but not AGS cells lacking Bak, suggesting it is Bak-activation that drives this loss. Subcellular fractionation showed detectable release of both Smac and cytochrome *c* into the cytosol of *Hp*-infected cells (Fig. 3e). A similar effect was observed by measuring total immunofluorescence in KATOIII cells; a trend to Bax/Bak-independent reduction in cytochrome *c*-fluorescence was also noted in these cells (Fig. 3f, Fig. S4C). In HeLa cells, we observed mitochondrial loss of Smac-GFP but retention of cytochrome *c* during *Hp*-infection (Fig. S4G) and found Bax/Bak-dependent loss of Smac and Bax/Bak-independent decrease in cytochrome *c*-levels (Fig. S4H). There was no detectable loss of mitochondrial membrane potential or mitochondrial mass during *Hp*-infection of AGS or KATOIII cells (Fig. S4D, E).

The results strongly suggest that Smac is released due to the sub-lethal action of Bax/Bak upon *Hp*-infection and is degraded in the cytosol. Cytosolic Smac is degraded by the proteasome [28]. During apoptosis, caspases counter-regulate degradation [23], probably through proteasome-inhibition [29]. We reproduced this effect: Smac was released from mitochondria by staurosporine-treatment, and Smac was lost in the presence of caspase-inhibitor (Fig. S4I). The low activity of caspases induced by *Hp* is likely insufficient for proteasomal inhibition, and therefore cytosolic Smac is degraded. Proteasome inhibition protected Smac from degradation (Fig. S4J). These results indicate that *Hp*-infection induces Bax/Bak-dependent, non-apoptotic mitochondrial Smac-release.

### Smac triggers the activation of NF-κB p100

**Fig. 4:**
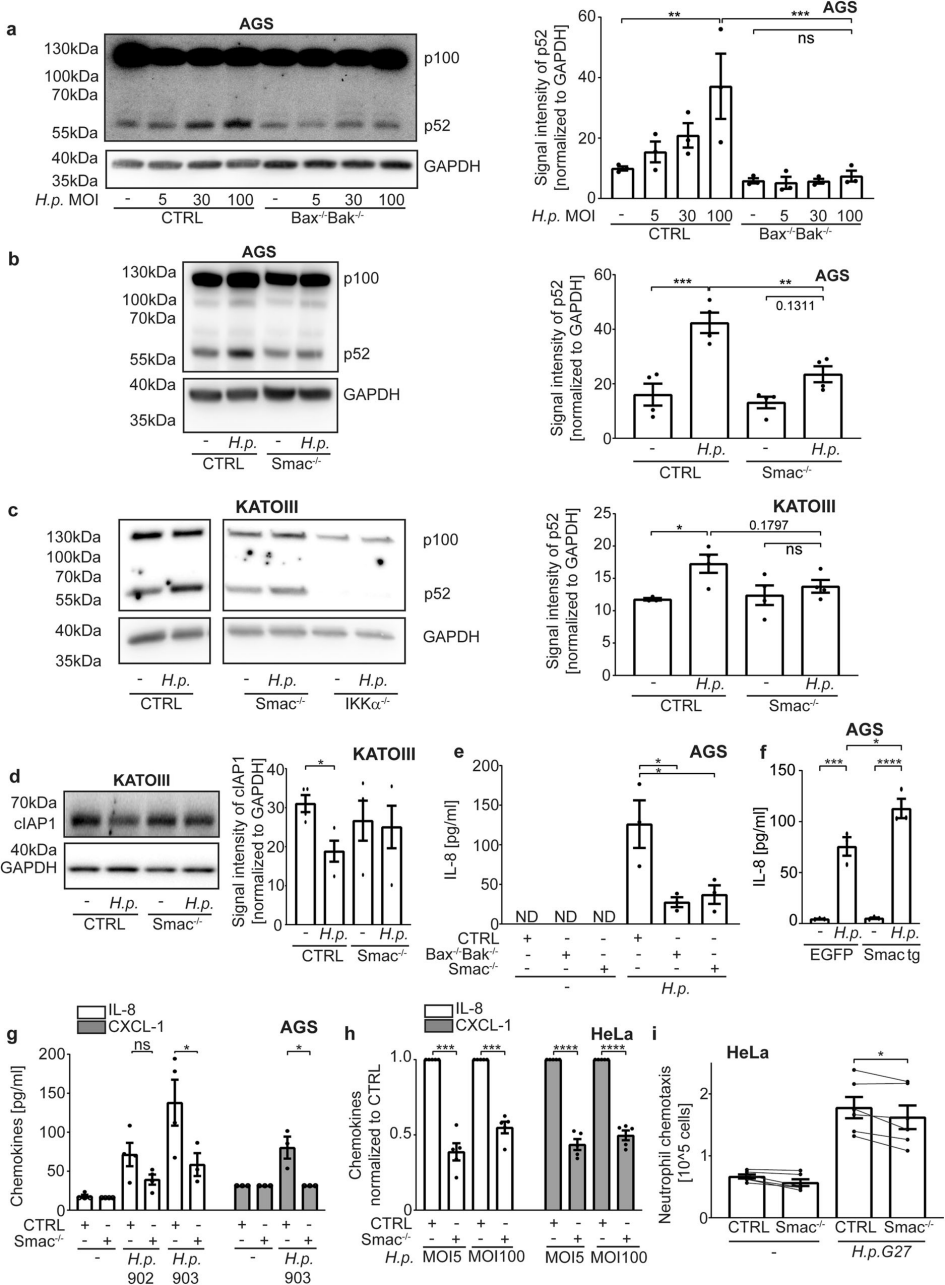
Smac is a mitochondrial mediator of host cell stimulation by H. pylori. **a**–**c** AGS cell lines (**a**: Bax^-/-^Bak^-/-^(clone 1); **b**: Smac^-/-^(gRNA 2)) and KATOIII (**c**) were infected with different MOI (**b**: MOI 100; **c**: MOI 10) of *H. pylori* G27 for 18 h (**a**, **b**) or 5 h (**c**). The inactive (p100) and active (p52) forms of NF-κB were detected by Western blotting. A representative Western blot and quantification of three (**a**) and four (**b**, **c**) independent experiments are shown. One data point was removed in **c** after Grubbs outlier testing. **d** KATOIII cell lines were infected with *H. pylori* G27 strain at an MOI of 10 for 5 h. cIAP1 was detected in whole cell lysates by Western blotting. Shown are a representative Western blot and quantification of four independent experiments. **e** AGS cell lines were infected with *H. pylori* G27 strain at an MOI of 100 for 6 h. IL-8 in cell culture supernatant was measured by ELISA in three independent experiments. **f** AGS Smac^−/−^ cells stably reconstituted with Smac (Smac tg) or stably expressing EGFP as a control were infected with *H. pylori* G27 strain at an MOI of 100 for 6 h. IL-8 in cell culture supernatants was measured by ELISA. Results are from three independent experiments. **g** AGS cell lines were infected with H. pylori clinical isolates 902, 903 at an MOI of 100 for 18 h. IL-8 and CXCL-1 were measured by ELISA in at least three independent experiments. The values of detection limit were used for statistical analyses if the measured chemokines were under detection limit. One data point was removed after Grubbs outlier testing. The experiment was conducted in parallel with the one shown in Fig. 1d, and control samples are identical. Not normalized data are shown in S8J. **h** HeLa cell lines were infected with different MOI of *H. pylori* G27 strain for 24 h. IL-8 and CXCL-1 were measured by ELISA. All infected samples were normalized to CTRL infected. Data are from five separate experiments. **i** HeLa cell lines were infected with *H. pylori* G27 strain at an MOI of 100 for 20 h. The cell culture supernatant was passed through a 0.2 µM filter. Human neutrophil chemotaxis was measured by transwell migration assay in six independent experiments. The experiment was conducted in parallel with the one shown in Fig. 1j, and control samples are identical. Lines connect the results from the same experiment. Data information: Bars represent the mean and dots the value of independent experiments. Hollow dots represent detection limit. Error bars show standard error of mean. Ns: *p* > 0.05, *, *p* < 0.05, **, *p* < 0.01, ***, *p* < 0.001, ****, *p* < 0.0001. The significance were tested by parametric one-way ANOVA (**a**, **b**, **c**, **g** [CXCL-1]**:** Sidak´s post hoc test**; e:** Dunnett´s post hoc test), ordinary one-way ANOVA (**f**), parametric two-way ANOVA (**g** [IL-8]: Dunnett´s post hoc test), one sample *T*-Test (**h**), unpaired *T*-Test (**d**) and paired *T*-Test (**i**). CTRL, non-targeting control gRNA; Bax^−/−^Bak^−/−^, double deletion of Bax and Bak by CRISPR/Cas9; IKKα^−/−^, deletion of IKKα by CRISPR/Cas9, Smac^−/−^, deletion of Smac by CRISPR/Cas9; Smac tg, Smac^-/-^ stably expressing Smac; EGFP, Smac^−/−^ stably expressing EGFP.

When experimentally expressed in the cytosol, Smac stimulates alternative NF-κB, as do small molecule Smac-mimetics [26, 30]. We hypothesized that Smac, released from mitochondria during sub-lethal apoptosis signalling, has the same activity. The activation of alternative NF-κB during *Hp*-infection (processing of p100 to p52), has been described in vitro, as has been its role in gene-deficient mice in vivo [31, 32]. We confirmed NF-κB p100-processing (Fig. 4a). Processing was not seen in Bax/Bak-deficient AGS (Fig. 4a) or HeLa (Fig. S5A) cells. Deletion of Smac reduced p100-processing in AGS (Fig. 4b, S5B), KATOIII (Fig. 4c) and HeLa cells (Fig. S5A; normal p100-processing in response to Smac-mimetic in Bax/Bak and Smac-mutant AGS cells was confirmed, Fig. S1M, N). Phosphorylation of NF-κB p65, i.e. classical NF-κB-signalling, was unaltered (Fig. S5C). We further performed reporter assays in HeLa cells. An NFκB-reporter construct was introduced into control, Bax/Bak-deficient and Smac-deficient cells. These cells showed induction of the reporter upon infection with Hp, and this induction was smaller in Bax/Bak and in Smac-deficient cells (Fig. S5D).

Smac-mimetic-signalling involves the inhibition of cIAP1/2, activating NIK and IKKα [26]. As predicted, KATOIII cells lacking IKKα showed no p100-processing (Fig. 4c). *Hp*-infection reduced cIAP1 but not XIAP-levels, depending on Smac in KATOIII cells (Fig. 4d, S5E). There was no clear difference in cIAP2-levels in KATOIII cells, and no reproducible loss of cIAP1/2 in AGS or HeLa cells (not shown). In AGS cells infected with the G27-strain, a contribution of Smac to IL-8-secretion was seen at an early time point (6 h, Fig. 4e) but not later on (18 h; not shown). Restoring Smac-expression in the Smac-deficient AGS cells also restored IL-8-secretion (Fig. 4f). Upon infection with the clinical isolate, chemokine secretion at 18 h also required a Smac-contribution (Fig. 4g). In HeLa cells, Smac-deficiency reduced secretion of IL-8 and CXCL1 (Fig. 4h) as well as migration-inducing capacity towards neutrophils (Fig. 4i). Thus, *Hp* causes the Bax/Bak-dependent release of Smac, which provides a major part of the p100-processing signal, and which contributes to chemokine secretion and neutrophil attraction.

### Hp -induced DNA-damage is due to sub-lethal apoptosis signalling

*Hp*-infection can cause DNA-strand breaks, which may contribute to malignant transformation [8]. Sub-lethal apoptosis signalling can activate the caspase-activated DNAse (CAD), causing DNA-damage and inducing permanent mutations [16]. CAD is activated by the caspase-mediated cleavage of its inhibitor ICAD [33]. Activation of CAD has been described in the published situations of sub-lethal apoptosis signalling, and the CAD-dependent DNA-damage response, detectable as the phosphorylation of the histone H2AX (γH2AX), is a very sensitive way to detect sub-lethal apoptosis signalling [16, 19].

**Fig. 5:**
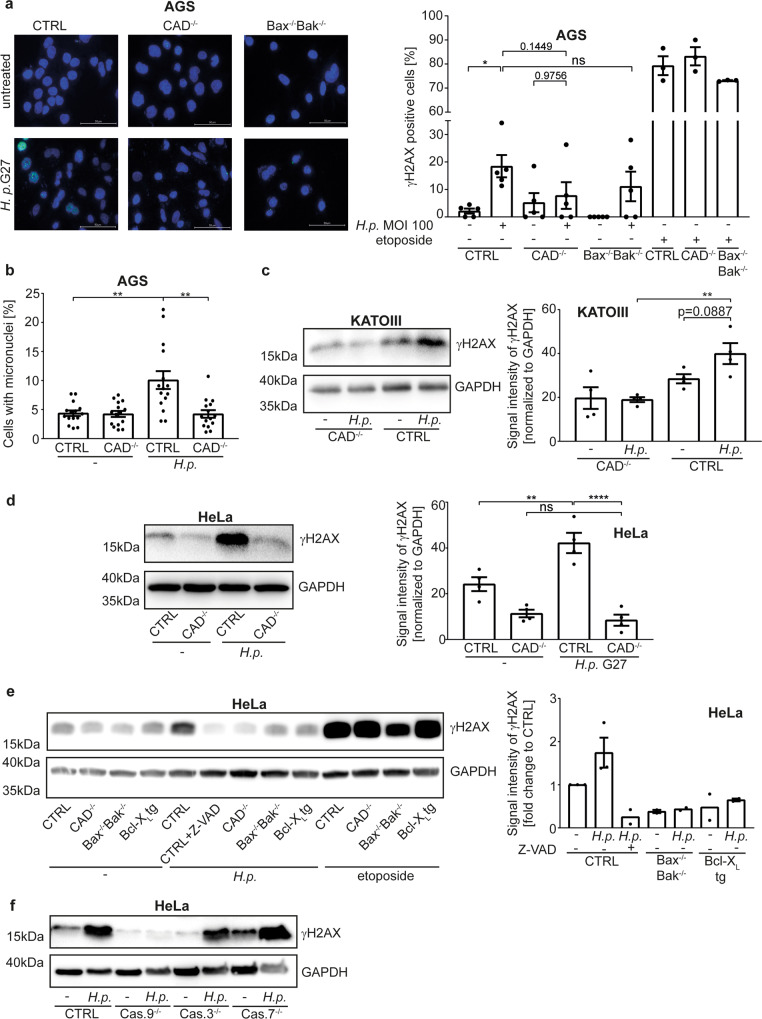
H. pylori causes DNA-damage and a DNA-damage response through the mitochondrial apoptosis pathway. **a** AGS cell lines were infected with *H. pylori* G27 strain at an MOI of 100 for 18 h. The DNA-damage response was measured as γH2AX-signal by immunofluorescence. Nuclei were counted positive for γH2AX if they had more than 4 dots. Shown are representative pictures and the quantification of five independent experiments with at least 250 cells per condition. The etoposide (10 µM, 18 h) positive control was done three times. Scale bars indicate 50 µM. **b** AGS cell lines were infected with *H. pylori* G27 strain at an MOI of 25 for 72 h. Micronuclei were detected by fluorescence microscopy after DNA staining with DAPI and cell staining with β-Tubulin. Shown are the values of the individual pictures of three independent experiments with at least 600 cells per condition. Significance was calculated with the mean value of each individual experiment. **c** KATOIII cell lines were infected with *H. pylori* G27 strain at an MOI of 10 for 5 h. The DNA-damage response was measured as γH2AX-signal by Western blot. Shown are representative Western blots and quantification of four independent experiments. **d**, **e** HeLa cell lines were infected with *H. pylori* G27 strain at an MOI of 100 for 18 h. The DNA-damage response was measured as γH2AX-signal by Western blot. Shown are representative Western blots and quantification of four (**d**) and at least two (**e**) independent experiments. Not normalized data are shown in S8K. **f** HeLa cell lines deficient in caspase-9 (Cas.9^−/−^), caspase-3 (Cas.3^−/−^) or caspase-7 (Cas.7^−/−^) were infected with *H. pylori* G27 strain at an MOI of 100 for 24 h. The DNA-damage response was measured as γH2AX-signal by Western blot. Shown are representative Western blots of three (Cas.9^−/−^, Cas.7^−/−^) or two (Cas.3^−/−^) independent experiments. Data information: Bars represent the mean and dots the value of independent experiments (**a**, **c**, **d**, **e**) or of micronuclei per picture (**b**). Error bars show standard error of mean. Ns: *p* > 0.05, *, *p* < 0.05, **, *p* < 0.01. Significance was tested by parametric one-way ANOVA (Sidak´s post hoc testing). CTRL non-targeting control gRNA; Bax^−/−^Bak^−/−^, double deletion of Bax and Bak, by CRISPR/Cas9; Bcl-X_L_, overexpressing Bcl-X_L_; CAD^−/−^, deletion of CAD by CRISPR/Cas9; Cas.9^−/−^, deletion of caspase-9 by CRISPR/Cas9; Cas.3^−/−^, deletion of caspase-3 by CRISPR/Cas9; Cas.7^−/−^, deletion of caspase-7 by CRISPR/Cas9. Z-VAD, the pan-caspase-inhibitor z-VAD-fmk.

We hypothesized that sub-lethal apoptosis signalling may contribute to the DNA-damage during *Hp*-infection. As reported, there was a clear γH2AX-response in *Hp*-infected AGS cells. This response was almost abrogated in CAD-deficient cells (Fig. 5a, S6A,B; two bacterial strains were used). The γH2AX-response was also reduced in Bax/Bak-deficient AGS cells (Fig. 5a, S6A). To test for actual DNA-damage, we scored infected AGS cells for micronuclei, which can form when a cell with damaged genomic DNA goes through mitosis [34, 35]. *Hp*-infection of AGS cells caused the CAD-dependent formation of micronuclei (Fig. 5b). The γH2AX-signal was abolished by the deletion of CAD in KATOIII (Fig. 5c) and HeLa cells (Fig. 5d, e) and not detectable in HeLa cells when caspase activity was inhibited or mitochondrial apoptosis had been disabled (Bax/Bak-deficiency or Bcl-X_L_-overexpression, Fig. 5e, S6C,D). The γH2AX-signal was not seen in cells deficient in caspase-9, while individual caspase-3- or caspase-7-deficiency did not block the signal (Fig. 5f). Thus, *Hp-*infection induces DNA-damage and a DNA-damage-response through the sub-lethal activation of the mitochondrial apoptosis apparatus and CAD. Both caspase-3 and -7 appear to be able to activate CAD downstream of caspase-9.

### Upstream signals in the engagement of mitochondria

Our results show that *Hp*-recognition by epithelial cells generates a signal that triggers the release of mitochondrial Smac. To approach the question of the upstream signals, we analysed bacterial factors and host cell receptor candidates. We infected AGS or HeLa cells with *Hp-*mutants lacking CagA, the Cag-pathogenicity island (PAI) or the adhesion protein BabA. As has been reported, the appearance of γH2AX and the activation of alternative NF-κB depended on the PAI and BabA but not CagA (Fig. S7A–D) [8, 31, 36]. Two receptor systems can mediate the PAI-dependent recognition of *Hp* in human cells. The TIFA-signalling axis acts as a recognition machinery of the LPS biosynthesis precursor, heptose-1,7-bisphosphate [37, 38], and NOD1 responds to *Hp* peptidoglycan fragments (muropeptides) [39].

**Fig. 6:**
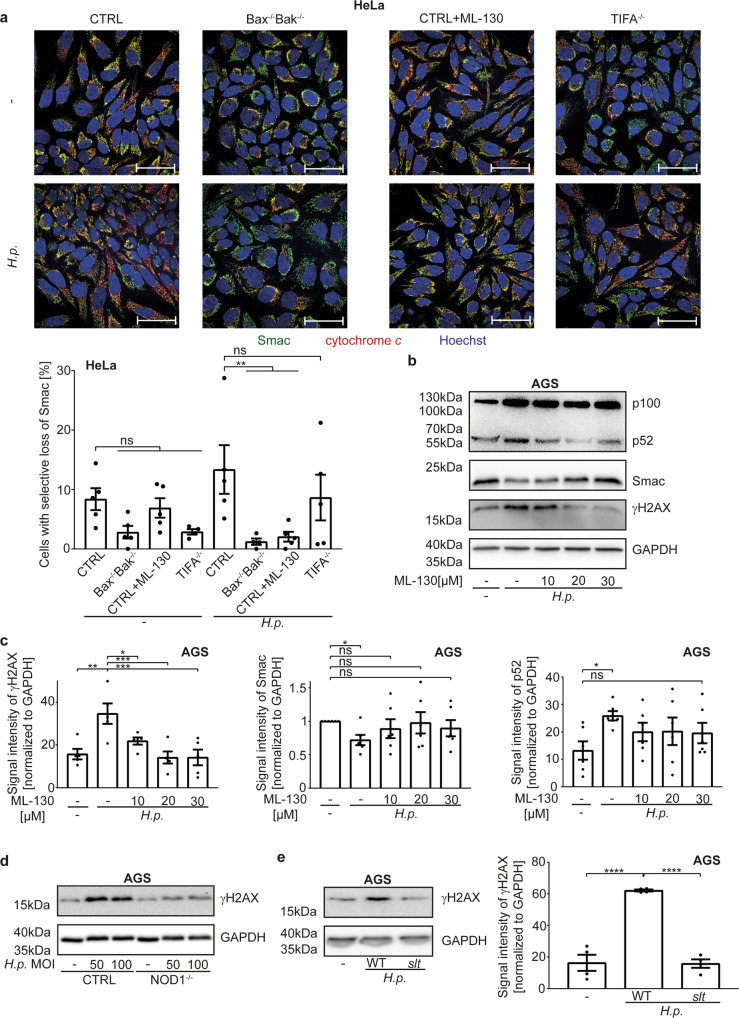
NOD1 activated by H. pylori induces low-level activation of the apoptosis apparatus. **a** HeLa cell lines were infected with *H. pylori G27* strain at an MOI of 100 for 18 h. The NOD1-inhibitor ML-130 was added to CTRL cells at the same time as the *H. pylori* infection. Endogenous levels of Smac (green) and cytochrome *c* (red) were detected by immunofluorescence. Shown are representative pictures and quantification of five independent experiments with at least 640 cells per condition. Two outliers were removed after Grubbs outlier testing. Scale bars represent 50 µm. A larger magnification and individual fluorescence channels are shown in Fig. S7E. **b**, **c** AGS cell lines were infected with *H. pylori G27* at an MOI of 100 for 18 h. Various concentrations of the NOD1-inhibitor ML-130 were added at the same time as the *H. pylori* infection. Processing of NF-κB p100 to p52, endogenous level of Smac in whole cell lysates and the DNA-damage response by appearance of a γH2AX-signal were measured by Western blotting. Shown is a representative Western blot (**b**) and quantification (**c**) of five individual experiments. Not normalized data are shown in S8I. **d** AGS CTRL or NOD1^−/−^ cells were infected with *H. pylori* G27 at an MOI of 100 for 24 h. The DNA-damage response by appearance of a γH2AX-signal was measured by Western blotting. Shown is one Western blot representative of two independent experiments. **e** AGS cell lines were infected at an MOI of 100 using a *H. pylori* T26695 deletion strains in lytic transglycosylase activity or wild type strain for 18 h. The DNA-damage response was measured as γH2AX-signal by Western blotting. Shown is a representative Western blot and quantification of three independent experiments. Data information: Bars represent the mean and dots the value of independent experiments. Error bars show standard error of mean. Ns: *p* > 0.05; **, *p* < 0.01; ***: *p* < 0.001. Significance was tested by two-way ANOVA (**a**), one-way ANOVA (**c**, **e**: Dunnett´s post hoc test; **c**: Sidak´s post hoc test) or one sample *T*-Test (**c**). CTRL, non-targeting control; NOD1^−/−^, deletion of NOD1 by CRISPR/Cas9; *slt*, T26695 deletion strains in lytic transglycosylase activity; WT, *H. pylori* T26695 wild typ; ML-130, NOD1 inhibitor.

Loss of Smac upon *Hp*-infection (Fig. 6a, S7E, G, H) and γH2AX-induction (Fig. S7G) were unaltered in TIFA-deficient cells (as a control in HeLa cells, Smac-loss is also shown, Fig. 6a, S7E). We used a chemical inhibitor of NOD1, ML-130 [40]. This inhibitor blocked release (Fig. 6a, S7E) and loss of Smac (Fig. 6b, c), as well as the activation of NF-κB p100 (Fig. 6b, c) and the γH2AX-signal in AGS cells (Fig. 6b, c). The NOD1-inhibitor reduced p100-upregulation, the appearance of p52 and Smac-loss also in HeLa cells (Fig. S7F). Genomic deletion of the NOD1-gene further reduced IL-8-secretion (Fig. S4F) and the γH2AX-DNA-damage response in AGS cells (Fig. 6d). The signaling pathway upstream of mitochondria therefore appears to originate from NOD1 while TIFA, which is required for the activation of classical NF-κB upon *Hp*-infection, does not partake in mitochondrial signaling. The ligands of NOD1 are *Hp* peptidoglycan-fragments (muropeptides). While it has not been possible to generate a peptidoglycan-deficient strain of *Hp*, a strain deficient in lytic transglycosylase activity, which has a defect in the release of the NOD1-binding muropeptides, has been found to be less active in NOD1-dependent induction of IL-8 in AGS cells [39]. This strain had lost the ability to generate a γH2AX-signal upon infection of AGS cells (Fig. 6e), consistent with the interpretation that NOD1 is required to drive this signalling.

### Evidence of sub-lethal apoptosis signaling in Hp-patients

**Fig. 7:**
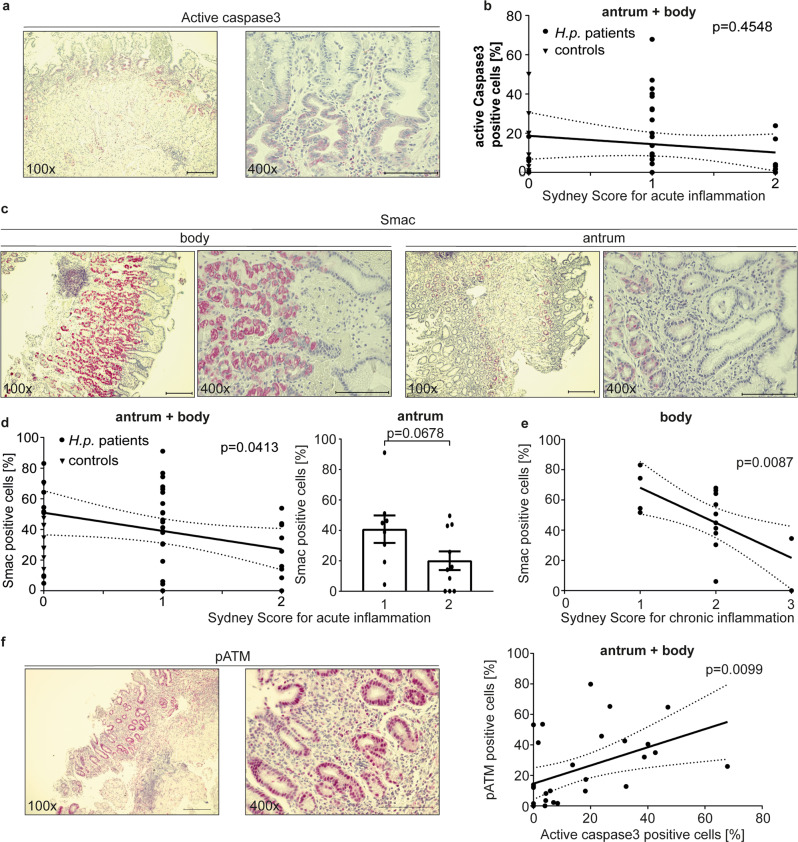
Detection of active caspase-3 and Smac in gastric biopsies from patients infected with H. pylori. **a**, **c** Biopsies of the human stomach were stained for the presence of active caspase-3 (**a**; using an antibody that recognizes only the cleavage product of caspase-3-activation) and Smac (**c**) by immunohistochemistry. Shown are representative images from a collection of 36 biopsies from 18 patients (20 antrum, 16 gastric body samples) infected with *H. pylori* and 17 patients without *H. pylori* infection (samples are from bariatric surgery) for each of the stains. Scale bar indicates 200 µm for 100x magnification and 100 µm for 400x magnification. Throughout the figure, the red stain identifies a positive antibody reaction. **b** The gastric biopsies described in **a** were scored for acute inflammation and correlated to the levels of staining for active caspase-3 (percent positive cells). At least 400 epithelial cells were counted for each biopsy. Each dot represents one biopsy. Sydney score for acute inflammation was calculated as described (Caselli et al., 1990). **d**, **e** Biopsies as in (**a**–**c**) were subjected to immunohistochemistry using an antibody specific for Smac. A total of 36 biopsies from 18 patients infected with *H. pylori* (gastric antrum (*n* = 20) and body (*n* = 16)) and 17 patients without *H. pylori* infection (samples are from bariatric surgery) were included as in (**a**). Smac-positive epithelial cells were scored in pictures taken as high power fields. At least 400 epithelial cells were counted for each biopsy. Sydney scores for acute (**d**) and chronic (**e**) inflammation were calculated and were correlated to Smac levels. No significant correlation was observed for acute inflammation in the gastric body or chronic inflammation in the antrum (not shown). Each dot represents one biopsy. **f** Biopsies of the human stomach were stained for the presence of DNA-damage response phosphorylated ATM by immunohistochemistry. Shown are representative images from a collection of 36 biopsies from 18 patients (20 antrum, 16 gastric body samples) infected with *H. pylori* for each of the stains. Scale bar indicates 200 µm for 100x magnification and 100 µm for 400x magnification. The gastric biopsies were correlated to the levels of staining for active cspase-3 (percent positive cells). At least 400 epithelial cells were counted for each biopsy. Each dot represents one biopsy. Data information: Bars represent the mean and dots the value of biopsies. Error bars show standard error of mean. Continuous line represents the linear regression of the data values. Interrupted lines represent the 95% confidence interval of linear regression. Ns: *p* > 0.05, *, *p* < 0.05, **, *p* < 0.01. The significance were tested by linear regression analysis (**b**, **d**, **e**, **f**) or unpaired *T*-Test (**d**).

We analyzed biopsies from a cohort of *Hp*-positive gastritis patients. Most patients showed histological evidence of gastritis (Sydney score for acute inflammation of 1–2, Fig. 7a, d, for chronic inflammation 1–3, Fig. 7e). In a substantial number of samples we detected epithelial cells in the gastric glands that gave a clear signal when stained with an antibody recognizing active caspase-3. The frequency of positive cells varied (mostly in the range of 5–50%); typically, large fractions of the cells in the neck region of the gastric glands, where *Hp* is commonly seen, were positive (Fig. 7a). Hardly any apoptoses were observed (under 1% of cells). No correlation between caspase-3-activation and inflammatory score was observed (Fig. 7b). Parietal cells gave a generally stronger Smac-signal, and the corpus (more parietal cells) was more strongly positive than the antrum (Fig. 7c). Intriguingly, the overall proportion of Smac-positive cells inversely correlated with acute and chronic inflammatory scores, with a similar trend for acute inflammation in the antrum and chronic inflammation in the body separately (Fig. 7d, e). This is reminiscent of the Smac content of cell lines, where *Hp* -infection reduced Smac. Biopsies from patients without (known) acute gastric inflammation, who had undergone gastric resection during bariatric surgery, were also stained and showed low levels of caspase-3-positive cells (Fig. 7b). In vitro, the most sensitive parameter of sub-lethal signals in the apoptosis pathway has been the DNA-damage response. We tested for DNA-damage response in these patient samples, using phosphorylation of ATM-kinase as a read-out. The signal for pATM showed a clear correlation with the level of cells expressing active caspase-3 (Fig. 7f), consistent with the model where *Hp* activates caspase-3 to a sub-lethal level of activity, causing DNA-damage and a DNA-damage response.

## Discussion

It is clear now that the mitochondrial apoptosis pathway can be triggered to sub-lethal activity but potential physiological roles and their pathways need to be worked out. This study shows that the pathway is triggered by a PRR during infection with a common bacterium, which causes chronic and oncogenic inflammation. It identifies a signalling role for Smac: Smac can be released in non-apoptotic cells and assume the pro-inflammatory role previously identified for Smac-mimetics. The results further suggest that sub-lethal mitochondrial apoptosis signals contribute to the development of infection-associated cancer.

We detected release of small amounts of both cytochrome *c* and Smac upon *Hp*-infection, and the data suggest that Smac was preferentially released. How such preferential release of Smac is achieved is unclear: during apoptosis, Smac-release occurs alongside the release of cytochrome *c* [23]. However, loss of the mitochondrial fission protein Drp1 reduced the release of cytochrome *c* but not Smac [41], and the apoptotic release of cytochrome *c* itself is far from clear [42]. Release of Smac has been reported in human cells infected with *Shigella* bacteria, where it has been suggested to block anti-bacterial immunity through interference with XIAP [43].

Most information of a pro-inflammatory role of Smac comes from investigations of Smac-mimetics, with some studies of experimental overexpression of Smac. In these studies, a dramatic cIAP-downregulation has regularly been observed (see for example [26]). We observed a relatively small loss of cIAP1 only in one of the cell lines we used, although deletion and reconstitution experiments clearly identified a role of Smac in IL-8-secretion upon *Hp*-infection. It seems conceivable that the generation of a signal activating alternative NF-κB through Smac may occur in the absence of obvious loss of cIAPs, especially if classical NF-κB is activated at the same time. Indeed, it may be the case that the massive loss of these proteins that are seen during Smac-mimetic stimulation is not necessarily what occurs during perhaps physiological, small stimuli. Further, cIAP-levels may be concurrently up-regulated by other signalling pathways, such as canonical NF-κB [44] or, as recently shown for cIAP2 and *Hp*-infection, through Brd4 [45].

Our data suggest a model where peptidoglycan, whose delivery required PAI but not CagA, as well as bacterial adhesion, stimulate NOD1 as the most upstream sensor. It has been reported earlier that p100/p52-processing [31] as well as the induction of DNA-damage [8, 36] depend on these bacterial factors. Additional factors are likely involved: at least one of our clinical isolates had reduced activity in activating sub-lethal apoptosis signals. Which such factors may be involved is uncertain; it is known *Hp* can evolve heavily in individual subjects over time and alter for instance its pro-inflammatory activity [46]. How *Hp* regulates its activities in the human stomach during long-term infection is an intriguing question, and how this is linked to the induction of sub-lethal signals in the mitochondrial apoptosis pathway will need careful future investigation.

It may be surprising that a PRR, a receptor from a class much better known to activate NF-κB and interferon responses, triggers the mitochondrial apoptosis apparatus. However, there are many examples where in experimental situations PRR can cause apoptotic cell death. This has been found for NOD1 itself [47], for a number of Toll-like receptors [48–51], RIG-I and Mda5 [52] and cGAS/STING [53]. It seems conceivable that the generation of sub-lethal apoptosis signals is an activity that is common to a number of pattern recognition receptors and that adds to immune activation.

The direct introduction of DNA-damage by *Hp* required the mitochondrial apoptosis apparatus. Our results suggest that mitochondrial signals have physiological functions in signalling immune alert in non-professional immune cells. At the same time, chronic signalling through Smac and NF-κB may contribute to malignant transformation, as has been proposed for modifications to XIAP [54]. The accompanying DNA-damage may be a necessary side effect that is outweighed by the benefit of Smac-dependent immune activation. The carcinogenic effect of bacterial infection is small: *Hp* -infection is a significant but small risk factor for gastric carcinoma, and cancer development typically takes many years. This supports the interpretation that indeed CAD-induced DNA-damage remains at an acceptable level, given the probably more important immune function of Smac-release.

## Material and methods

### Cell lines and cell culture

AGS cells (89090402-1VL, Sigma-Aldrich) were cultured in Ham´s F-12K medium (21-127-022, Thermo Fisher Scientific), HeLa and KATOIII in RPMI 1640 medium (61870044, Life Technologies) with 10% FCS. CRISPR-Cas9 (52961, Addgene) genome editing was done as described previously [19]; cells deficient in CAD, Bax/Bak were established using the gRNAs described [19]. Guide RNAs used here were Smac1 (TTAGTAGTGAAGCATTGATG), Smac2 (GTGCAATAGGAACCGCACAC), TIFA1 (GAAACTCCCTTCCAGCGAAG) and TIFA2 (CATCCTGGCCAGTTGCAGTG) caspase-3 (ATTGTGGAATTGATGCGTGA), caspase-7 (TGTACTGATATGTAGGCACT) (Human CRISPR Knockout Pooled Library (Brunello) [55]); NOD1 (GCAACTCGCAGATGCCTACG), Ikkα (ACAGACGTTCCCGAAGCCGCCGG), Caspase-9 (ATCTCCTGCTTAGAGGACAC) was designed using GPP web portal (Broad Institute). HeLa cell lines overexpressing Bcl-X_L_ have been described [19]. Reconstitution of human Smac in AGS Smac knockout cells (Smac2 gRNA used for Knockout) was done by cloning human Smac (mutated in the gRNA sequence) into lentiviral construct pEF1-GW-Puro-hSmac. All knock outs were tested by western blotting (Fig. S1a–k). NOD1 knockout was confirmed by sequencing (Fig. S1k).

### Bacterial culture and infection

*Hp* strains G27, T26695 and isogenic mutants were provided by Wolfgang Fischer and Rainer Haas, LMU Munich. The slt-deficient and its parental strain were from Ivo Gomperts Boneca, Paris. Bacteria were cultured at 37 °C, 5% O_2_ and streaked freshly the day prior to infection. Bacteria were diluted in Brucella browth. Infection was controlled microscopically.

### Antibodies

Antibodies were against active caspase-3 (clone C92-605, 559565, BD; clone5A1E, Cell Signaling), phospho-ATM (S1981, Abcam), beta-tubulin (9F3,2128 L, NEB), α-tubulin (Sigma-Aldrich, #t9026), Bax (Cell Signaling #2772), Bak (Cell Signaling #21105), Bcl-X_L_ (54H6, Cell Signaling), CAD (PA5-19913, Thermo Fisher, and CAD (F11), SantaCruz sc-374067), caspase-3 (Cell Signaling #9662), caspase-7 (Cell Signaling #9494), caspase-9 (Cell Signaling, #9502), cIAP1 (D5G9, Cell Signaling), cytochrome *c* (D18C7, Cell Signaling), cytochrome *c* (6H2B4, Cell Signaling), GAPDH (MAB374, Millipore), γH2AX (2577 L, Cell Signaling), IKKα (Cell Signaling #2682), NF-κB p100/p52 (18D10, Cell Signaling), phospho-NF-κB p65 (93H1, Cell Signaling), Smac (#15108 or #2954, Cell Signaling), TIFA (CSB-PA839301LA01HU, Cusabio), VDAC (Cell Signaling #4661), XIAP (Cell Signaling #14334). Secondary antibodies: anti mouse IgG-Cy5 (715-175-151, Dianova), donkey anti rabbit IgG-Alexa Fluor647 (711-605-152, Dianova), anti rabbit IgG-Alexa488 (711-545-152, Dianova), anti mouse IG-HRP (115-035-166, Dianova), anti rabbit-HRP (A6667, Sigma).

### Reagents

DAPI, etoposide, Hoechst, PMA, staurosporine (Sigma); Fugene (Promega), LCL161 (Active Biochem), Mito Tracker Deep Red (Life Technologies), ML-130 (Tocris), Z-VAD-fmk (Gentaur) were used as indicated.

### ELISA

We identified soluble AGS-products in a screen by bead array (Eve Technologies, Calgary). Cytokines in supernatants were measured by ELISA: IL-8 (Biolegend), CXCL-1 (RnD Systems), VEGF-α (Boster Bio).

### Primary peripheral blood neutrophils

Neutrophils were obtained from healthy adult volunteers by negative selection with a magnetic cell separation system (EasySep kit, Stem Cell Technologies). Purity of cell preparations was confirmed by Giemsa staining.

### Transwell migration assay

A 24-well transwell system (3 µm pore, Corning Costar) was used. HeLa cell supernatants (400 µl) were added into the lower chamber, and 3.5 × 10^5^ freshly isolated neutrophils in 200 µl complete medium were placed into the upper chamber. Negative controls (medium) and positive controls (human IL-8 (5 ng/ml)) were included. After 75 min incubation migration was stopped. Cells in the lower chamber were harvested and counted (CASY cell counter, Omni Life Science).

### Neutrophil function

Neutrophils (3 × 10^5^/300 µl) were co-incubated with supernatants from AGS cells infected with *Hp* G27 (diluted 1:2) for 24 h. Cells were harvested, stained with Annexin V-FITC (Thermo Fisher) and Live/ Dead Fixable Far Red Dye (Thermo Fisher), fixed in 4% PFA (Morphisto) and analyzed by flow cytometry (FACS Calibur, BD). Supernatants from neutrophils were collected for ELISA.

### Caspase activity assays

Cells (1.5 × 10^5^) were seeded and infected in duplicates in 6-well plates, fixed in 4% formalin and stained for active caspase-3. Caspase-3 reporter cells have been described [19]. Analyses were performed with a FACS Calibur (BD). For enzyme assay, cells were pooled from duplicates and lysed (buffer 9803, Cell Signaling/ protease inhibitors (Roche)). Ten µl of lysate were incubated with reaction buffer (90 µl, MDB buffer, 11 µM Ac-DEVD-AMC (Bachem), 100 µg/ml BSA, 0,1% CHAPS) in triplicates. Analyses were performed with a Spark 10 M (Tecan). For precipitation of active caspases, biotinylated VAD-fmk (Santa Cruz) was added 3 h before harvesting. Cells were lysed with RIPA buffer. Aliquots were boiled at 95 °C in Laemmli buffer. Supernatants were incubated with neutravidin beads (Thermo Fischer) at 4 °C overnight. The beads were washed with RIPA buffer. Laemmli buffer was added and beads were boiled, followed by Western blotting for active caspase-3.

### LDH-release assay

Cells (6 × 10^4^) were seeded in 24-well plates. LDH concentration in filtered supernatants was measured by cytotoxicity detection kit (Roche). Cytotoxicity was calculated as ratio of experiment value after background reduction divided by Triton X-100 lysed cells.

### Colony assay

Cells (1.5 × 10^5^) were seeded in 6-well plates. Following infection, cells were counted and plated (500 cells/well) in triplicates (medium contained 100U/ml Ampicillin). Seven days later, colonies were stained with cristal violet and counted.

### Immunofluorescence

Cells were fixed on IBIDI-slides and permeabilized with 0.2% Triton-X100 in PBS (Smac and cytochrome *c*) or with methanol at −20 °C (γH2AX). Staining was done by consecutive incubation with primary and secondary antibody in the same buffer. Nuclei were stained with Hoechst. Pictures were taken blinded with a Zeiss LSM 880 (Smac, cytochrome *c*) or with a Keyence BZ-9000 (γH2AX). γH2AX dots per cell were counted with ImageJ. In KATOIII cells, corrected total cell fluorescence (CTCF) was determined with ImageJ. Photos of at least 50 Smac and cytochrome *c*-co-stained cells per condition and per experiment were acquired (Zeiss LSM 880). CTCF was calculated using the formula CTCF = integrated density − (area of selected cell x mean fluorescence of background readings).

### Western blotting

Cells (1.5 × 10^5^) were seeded in 6-well plates. Cells were lysed with Laemmli buffer in the wells. Samples were sonicated and heated to 95 °C before loading to SDS PAGE. PVDF membranes were blocked with 5% milk. Proteins were detected with ECL substrate. Signal intensity was calculated with ImageJ. Full length original western blots are provided in Supplementary File 1.

### Subcellular fractionation to analyze cytochrome c and Smac subcellular localization

AGS cells containing an empty vector were either mock- or Helicobacter-infected (G27, MOI 100) for 18 h to assess the release of Smac and cytochrome *c*. Cells were harvested, washed and resuspended in MB-EDTA buffer. Mitochondria were obtained by flashing cells through a 27 G needle using 1 mL syringe as described [56]. Mitochondrial fractions were isolated and supernatants were centrifuged for 60 min at 4 °C and 120,000xg. The resulting supernatants (cytoplasmic fractions) together with mitochondrial fractions were analyzed by immunoblotting using VDAC and α-tubulin as marker proteins for mitochondrial and cytoplasmic fractions.

### Micronuclei assay

AGS cells in IBIDI 8 µm microscopy well were fixed with 4% PFA and stained with anti-tubulin antibody and DAPI in 1% BSA/0.1% Tween-20/PBS. Approximately 200 cells per experiment in five pictures (Zeiss LSM 880 confocal microscope) were analyzed in a blinded fashion.

### Human stomach samples

Sampling of biopsies from *Hp* -infected patients was approved by the local ethics board. Thirty-six samples from 18 patients (20x antrum, 16x body) were analyzed. Tissues were blocked and immunostained for Smac, pATM and cleaved caspase-3 using a Dako detection system and counterstained with hematoxcylin. Epithelial cells (at least 400 cells per biopsy) were counted in high power fields of representative areas. Inflammation was scored in H&E stains using the Sydney classification [57].

### Statistics

Statistics were calculated with Prism (V7, GraphPad). Unpaired *T*-test was used when comparing two samples. One-way and two-way ANOVA were used for multiple testing. Normalized data were analyzed by one-sample *T*-test. All statistical tests were performed two-sided. Linear regression was used to compare biopsies from *Hp-*infected patients.

## Supplementary information

Supplementary figure legends

Suppl. FigS1

Suppl. FigS2

Suppl. FigS3

Suppl. FigS4_part1

Suppl. FigS4_part2

Suppl. FigS5

Suppl. FigS6

Suppl. FigS7_part1

Suppl. FigS7_part2

Suppl. FigS8_part1

Suppl. FigS8_part2

Supplementary File 1

Reproducibility Checklist

Pre-authorship form

## Data Availability

All data supporting the findings of this study are available from the corresponding author upon reasonable request. Information on the human stomach biopsies can be accessed upon request from the CCCF tumour bank Freiburg.

## References

[1] Suerbaum S, Michetti P. Helicobacter pylori infection. N Engl J Med. 2002;347:1175–86. 12374879 10.1056/NEJMra020542

[2] Peek RM Jr, Blaser MJ. Helicobacter pylori and gastrointestinal tract adenocarcinomas. Nat Rev Cancer. 2002;2:28–37. 11902583 10.1038/nrc703

[3] Odenbreit S, Puls J, Sedlmaier B, Gerland E, Fischer W, Haas R. Translocation of Helicobacter pylori CagA into gastric epithelial cells by type IV secretion. Science. 2000;287:1497–1500. 10688800 10.1126/science.287.5457.1497

[4] Segal ED, Cha J, Lo J, Falkow S, Tompkins LS. Altered states: involvement of phosphorylated CagA in the induction of host cellular growth changes by Helicobacter pylori. Proc Natl Acad Sci USA. 1999;96:14559–64. 10588744 10.1073/pnas.96.25.14559 PMC24475

[5] Blosse A, Lehours P, Wilson KT, Gobert AP. Helicobacter: Inflammation, immunology, and vaccines. Helicobacter. 2018;23:e12517. 30277626 10.1111/hel.12517 PMC6310010

[6] Jenks PJ, Jeremy AH, Robinson PA, Walker MM, Crabtree JE. Long-term infection with Helicobacter felis and inactivation of the tumour suppressor gene p53 cumulatively enhance the gastric mutation frequency in Big Blue transgenic mice. J Pathol. 2003;201:596–602. 14648663 10.1002/path.1488

[7] Touati E, Michel V, Thiberge JM, Wuscher N, Huerre M, Labigne A. Chronic Helicobacter pylori infections induce gastric mutations in mice. Gastroenterology. 2003;124:1408–19. 12730880 10.1016/s0016-5085(03)00266-x

[8] Toller IM, Neelsen KJ, Steger M, Hartung ML, Hottiger MO, Stucki M, et al. Carcinogenic bacterial pathogen Helicobacter pylori triggers DNA double-strand breaks and a DNA damage response in its host cells. Proc Natl Acad Sci USA. 2011;108:14944–9. 21896770 10.1073/pnas.1100959108 PMC3169107

[9] Hanada K, Uchida T, Tsukamoto Y, Watada M, Yamaguchi N, Yamamoto K, et al. Helicobacter pylori infection introduces DNA double-strand breaks in host cells. Infect Immun. 2014;82:4182–9. 25069978 10.1128/IAI.02368-14 PMC4187860

[10] Hartung ML, Gruber DC, Koch KN, Gruter L, Rehrauer H, Tegtmeyer N, et al. H. pylori-Induced DNA strand breaks are introduced by nucleotide excision repair endonucleases and promote NF-kappaB target gene expression. Cell Rep. 2015;13:70–79. 26411687 10.1016/j.celrep.2015.08.074

[11] Bauer M, Nascakova Z, Mihai AI, Cheng PF, Levesque MP, Lampart S, et al. The ALPK1/TIFA/NF-kappaB axis links a bacterial carcinogen to R-loop-induced replication stress. Nat Commun. 2020;11:5117. 33037203 10.1038/s41467-020-18857-z PMC7547021

[12] Tummers B, Green DR. The evolution of regulated cell death pathways in animals and their evasion by pathogens. Physiol Rev. 2022;102:411–54. 34898294 10.1152/physrev.00002.2021 PMC8676434

[13] Pachathundikandi SK, Muller A, Backert S. Inflammasome activation by Helicobacter pylori and its implications for persistence and immunity. Curr Top Microbiol Immunol. 2016;397:117–31. 27460807 10.1007/978-3-319-41171-2_6

[14] Lovric MM, Hawkins CJ. TRAIL treatment provokes mutations in surviving cells. Oncogene. 2010;29:5048–60. 20639907 10.1038/onc.2010.242 PMC2997681

[15] Orth JD, Loewer A, Lahav G, Mitchison TJ. Prolonged mitotic arrest triggers partial activation of apoptosis, resulting in DNA damage and p53 induction. Mol Biol cell. 2012;23:567–76. 22171325 10.1091/mbc.E11-09-0781 PMC3279386

[16] Ichim G, Lopez J, Ahmed SU, Muthalagu N, Giampazolias E, Delgado ME, et al. Limited mitochondrial permeabilization causes DNA damage and genomic instability in the absence of cell death. Mol cell. 2015;57:860–72. 25702873 10.1016/j.molcel.2015.01.018 PMC4352766

[17] Miles MA, Hawkins CJ. Executioner caspases and CAD are essential for mutagenesis induced by TRAIL or vincristine. Cell death Dis. 2017;8:e3062. 28981092 10.1038/cddis.2017.454 PMC5680576

[18] Hacker G. Apoptosis in infection. Microbes Infect / Inst Pasteur. 2018;20:552–9. 10.1016/j.micinf.2017.10.006 29109017

[19] Brokatzky D, Dorflinger B, Haimovici A, Weber A, Kirschnek S, Vier J, et al. A non-death function of the mitochondrial apoptosis apparatus in immunity. EMBO J. 2019;38:e102325. 30979778 10.15252/embj.2018100907 PMC6545560

[20] Maeda S, Yoshida H, Mitsuno Y, Hirata Y, Ogura K, Shiratori Y, et al. Analysis of apoptotic and antiapoptotic signalling pathways induced by Helicobacter pylori. Gut. 2002;50:771–8. 12010877 10.1136/gut.50.6.771 PMC1773255

[21] Potthoff A, Ledig S, Martin J, Jandl O, Cornberg M, Obst B, et al. Significance of the Caspase family in Helicobacter pylori induced gastric epithelial apoptosis. Helicobacter. 2002;7:367–77. 12485124 10.1046/j.1523-5378.2002.00112.x

[22] Chipuk JE, Moldoveanu T, Llambi F, Parsons MJ, Green DR. The BCL-2 family reunion. Mol cell. 2010;37:299–310. 20159550 10.1016/j.molcel.2010.01.025 PMC3222298

[23] Rehm M, Dussmann H, Prehn JH. Real-time single cell analysis of Smac/DIABLO release during apoptosis. J cell Biol. 2003;162:1031–43. 12975347 10.1083/jcb.200303123 PMC2172837

[24] Du C, Fang M, Li Y, Li L, Wang X. Smac, a mitochondrial protein that promotes cytochrome c-dependent caspase activation by eliminating IAP inhibition. Cell. 2000;102:33–42. 10929711 10.1016/s0092-8674(00)00008-8

[25] Verhagen AM, Ekert PG, Pakusch M, Silke J, Connolly LM, Reid GE, et al. Identification of DIABLO, a mammalian protein that promotes apoptosis by binding to and antagonizing IAP proteins. Cell. 2000;102:43–53. 10929712 10.1016/s0092-8674(00)00009-x

[26] Vince JE, Wong WW, Khan N, Feltham R, Chau D, Ahmed AU, et al. IAP antagonists target cIAP1 to induce TNFalpha-dependent apoptosis. Cell. 2007;131:682–93. 18022363 10.1016/j.cell.2007.10.037

[27] Silke J, Vucic D. IAP family of cell death and signaling regulators. Methods Enzymol. 2014;545:35–65. 25065885 10.1016/B978-0-12-801430-1.00002-0

[28] MacFarlane M, Merrison W, Bratton SB, Cohen GM. Proteasome-mediated degradation of Smac during apoptosis: XIAP promotes Smac ubiquitination in vitro. J Biol Chem. 2002;277:36611–6. 12121969 10.1074/jbc.M200317200

[29] Sun XM, Butterworth M, MacFarlane M, Dubiel W, Ciechanover A, Cohen GM. Caspase activation inhibits proteasome function during apoptosis. Mol cell. 2004;14:81–93. 15068805 10.1016/s1097-2765(04)00156-x

[30] Varfolomeev E, Blankenship JW, Wayson SM, Fedorova AV, Kayagaki N, Garg P, et al. IAP antagonists induce autoubiquitination of c-IAPs, NF-kappaB activation, and TNFalpha-dependent apoptosis. Cell. 2007;131:669–81. 18022362 10.1016/j.cell.2007.10.030

[31] Mejias-Luque R, Zoller J, Anderl F, Loew-Gil E, Vieth M, Adler T, et al. Lymphotoxin beta receptor signalling executes Helicobacter pylori-driven gastric inflammation in a T4SS-dependent manner. Gut. 2017;66:1369–81. 27196595 10.1136/gutjnl-2015-310783

[32] Maeda S, Yoshida H, Ogura K, Mitsuno Y, Hirata Y, Yamaji Y, et al. H. pylori activates NF-kappaB through a signaling pathway involving IkappaB kinases, NF-kappaB-inducing kinase, TRAF2, and TRAF6 in gastric cancer cells. Gastroenterology. 2000;119:97–108. 10889159 10.1053/gast.2000.8540

[33] Enari M, Sakahira H, Yokoyama H, Okawa K, Iwamatsu A, Nagata S. A caspase-activated DNase that degrades DNA during apoptosis, and its inhibitor ICAD. Nature. 1998;391:43–50. 9422506 10.1038/34112

[34] Harding SM, Benci JL, Irianto J, Discher DE, Minn AJ, Greenberg RA. Mitotic progression following DNA damage enables pattern recognition within micronuclei. Nature. 2017;548:466–70. 28759889 10.1038/nature23470 PMC5857357

[35] Mackenzie KJ, Carroll P, Martin CA, Murina O, Fluteau A, Simpson DJ, et al. cGAS surveillance of micronuclei links genome instability to innate immunity. Nature. 2017;548:461–5. 28738408 10.1038/nature23449 PMC5870830

[36] Koeppel M, Garcia-Alcalde F, Glowinski F, Schlaermann P, Meyer TF. Helicobacter pylori infection causes characteristic DNA damage patterns in human cells. Cell Rep. 2015;11:1703–13. 26074077 10.1016/j.celrep.2015.05.030

[37] Stein SC, Faber E, Bats SH, Murillo T, Speidel Y, Coombs N, et al. Helicobacter pylori modulates host cell responses by CagT4SS-dependent translocation of an intermediate metabolite of LPS inner core heptose biosynthesis. PLoS Pathog. 2017;13:e1006514. 28715499 10.1371/journal.ppat.1006514 PMC5531669

[38] Gall A, Gaudet RG, Gray-Owen SD, Salama NR. TIFA signaling in gastric epithelial cells initiates the cag Type 4 secretion system-dependent innate immune response to Helicobacter pylori infection. mBio. 2017;8:e01168-17. 28811347 10.1128/mBio.01168-17 PMC5559637

[39] Viala J, Chaput C, Boneca IG, Cardona A, Girardin SE, Moran AP, et al. Nod1 responds to peptidoglycan delivered by the Helicobacter pylori cag pathogenicity island. Nat Immunol. 2004;5:1166–74. 15489856 10.1038/ni1131

[40] Khan PM, Correa RG, Divlianska DB, Peddibhotla S, Sessions EH, Magnuson G, et al. Identification of Inhibitors of NOD1-Induced Nuclear Factor-kappaB Activation. ACS Med Chem Lett. 2011;2:780–5. 22003428 10.1021/ml200158b PMC3193285

[41] Ishihara N, Nomura M, Jofuku A, Kato H, Suzuki SO, Masuda K, et al. Mitochondrial fission factor Drp1 is essential for embryonic development and synapse formation in mice. Nat cell Biol. 2009;11:958–66. 19578372 10.1038/ncb1907

[42] Dewson G. Doughnuts, daisy chains and crescent moons: the quest for the elusive apoptotic pore. EMBO J. 2016;35:371–3. 26783365 10.15252/embj.201593723 PMC4755113

[43] Andree M, Seeger JM, Schull S, Coutelle O, Wagner-Stippich D, Wiegmann K, et al. BID-dependent release of mitochondrial SMAC dampens XIAP-mediated immunity against Shigella. EMBO J. 2014;33:2171–87. 25056906 10.15252/embj.201387244 PMC4282505

[44] Chu ZL, McKinsey TA, Liu L, Gentry JJ, Malim MH, Ballard DW. Suppression of tumor necrosis factor-induced cell death by inhibitor of apoptosis c-IAP2 is under NF-kappaB control. Proc Natl Acad Sci USA. 1997;94:10057–62. 9294162 10.1073/pnas.94.19.10057 PMC23303

[45] Chen Y, Sheppard D, Dong X, Hu X, Chen M, Chen R, et al. H. pylori infection confers resistance to apoptosis via Brd4-dependent BIRC3 eRNA synthesis. Cell death Dis. 2020;11:667. 32820150 10.1038/s41419-020-02894-z PMC7441315

[46] Jackson LK, Potter B, Schneider S, Fitzgibbon M, Blair K, Farah H, et al. Helicobacter pylori diversification during chronic infection within a single host generates sub-populations with distinct phenotypes. PLoS Pathog. 2020;16:e1008686. 33370399 10.1371/journal.ppat.1008686 PMC7794030

[47] Inohara N, Koseki T, del Peso L, Hu Y, Yee C, Chen S, et al. Nod1, an Apaf-1-like activator of caspase-9 and nuclear factor-kappaB. J Biol Chem. 1999;274:14560–7. 10329646 10.1074/jbc.274.21.14560

[48] Weber A, Kirejczyk Z, Besch R, Potthoff S, Leverkus M, Hacker G. Proapoptotic signalling through Toll-like receptor-3 involves TRIF-dependent activation of caspase-8 and is under the control of inhibitor of apoptosis proteins in melanoma cells. Cell death Differ. 2010;17:942–51. 20019748 10.1038/cdd.2009.190

[49] Ruckdeschel K, Pfaffinger G, Haase R, Sing A, Weighardt H, Hacker G, et al. Signaling of apoptosis through TLRs critically involves toll/IL-1 receptor domain-containing adapter inducing IFN-beta, but not MyD88, in bacteria-infected murine macrophages. J Immunol. 2004;173:3320–8. 15322195 10.4049/jimmunol.173.5.3320

[50] Fischer SF, Rehm M, Bauer A, Hofling F, Kirschnek S, Rutz M, et al. Toll-like receptor 9 signaling can sensitize fibroblasts for apoptosis. Immunol Lett. 2005;97:115–22. 15626483 10.1016/j.imlet.2004.10.015

[51] Aliprantis AO, Yang RB, Mark MR, Suggett S, Devaux B, Radolf JD, et al. Cell activation and apoptosis by bacterial lipoproteins through toll-like receptor-2. Science. 1999;285:736–9. 10426996 10.1126/science.285.5428.736

[52] Besch R, Poeck H, Hohenauer T, Senft D, Hacker G, Berking C, et al. Proapoptotic signaling induced by RIG-I and MDA-5 results in type I interferon-independent apoptosis in human melanoma cells. J Clin Investig. 2009;119:2399–411. 19620789 10.1172/JCI37155 PMC2719920

[53] Gulen MF, Koch U, Haag SM, Schuler F, Apetoh L, Villunger A, et al. Signalling strength determines proapoptotic functions of STING. Nat Commun. 2017;8:427. 28874664 10.1038/s41467-017-00573-w PMC5585373

[54] Palrasu M, Zaika E, El-Rifai W, Garcia-Buitrago M, Piazuelo MB, Wilson KT, et al. Bacterial CagA protein compromises tumor suppressor mechanisms in gastric epithelial cells. J Clin Investig. 2020;130:2422–34. 32250340 10.1172/JCI130015 PMC7190987

[55] Doench JG, Fusi N, Sullender M, Hegde M, Vaimberg EW, Donovan KF, et al. Optimized sgRNA design to maximize activity and minimize off-target effects of CRISPR-Cas9. Nat Biotechnol. 2016;34:184–91. 26780180 10.1038/nbt.3437 PMC4744125

[56] Wilfling F, Weber A, Potthoff S, Vogtle FN, Meisinger C, Paschen SA, et al. BH3-only proteins are tail-anchored in the outer mitochondrial membrane and can initiate the activation of Bax. Cell death Differ. 2012;19:1328–36. 22343714 10.1038/cdd.2012.9 PMC3392640

[57] Caselli M, Aleotti A, Barboni A, Alvisi V. Sydney classification for gastritis and Helicobacter pylori. Lancet. 1990;336:1445–6. 1978898 10.1016/0140-6736(90)93146-g

