# Correction to: Mitochondria supply sub-lethal signals for cytokine secretion and DNA-damage in *H. pylori* infection

**DOI:** 10.1038/s41418-022-01019-7

**Published:** 2022-05-16

**Authors:** Benedikt Dörflinger, Mohamed Tarek Badr, Aladin Haimovici, Lena Fischer, Juliane Vier, Arlena Metz, Bianca Eisele, Peter Bronsert, Konrad Aumann, Jens Höppner, Collins Waguia Kontchou, Ishita Parui, Arnim Weber, Susanne Kirschnek, Georg Häcker

**Affiliations:** 1 Faculty of Medicine, Institute of Medical Microbiology and Hygiene, Medical Center, University of Freiburg, Freiburg, Germany; 2 Faculty of Medicine, Institute for Surgical Pathology, Medical Center, University of Freiburg, Freiburg, Germany; 3 Comprehensive Cancer Center Freiburg, Medical Center, University of Freiburg, Freiburg, Germany; 4 Center for Pathology Allgäu, Kempten Allgäu, Germany; 5 Department of Surgery, University Medical Center Schleswig-Holstein, Lübeck, Germany; 6 BIOSS Centre for Biological Signalling Studies, University of Freiburg, Freiburg, Germany

**Keywords:** Cell death and immune response, Infectious diseases, Acute inflammation, Infectious diseases, Cancer

Correction to: *Cell Death & Differentiation* 10.1038/s41418-022-01009-9, published online 03 May 2022

The original version of this article contained a mistake. The following funding information was unfortunately lost during production: “This work was supported by the MOTI-VATE graduate school of the Freiburg University Medical School (grant to BD) and the Deutsche Forschungsgemeinschaft (DFG, German Research Foundation) through the IMM-PACT-Program for Clinician Scientists– 413517907 (grant to MTB) and a grant to GH (HA 2128). Part of the project was supported by the Wilhelm Sander-Stiftung (2019.151.1).” We apologize for the error. The original article has been corrected.

