# Supplementary material for: Mitochondria supply sub-lethal signals for cytokine secretion and DNA-damage in H. pylori infection
Source: Cell Death Differ. 2022 May 3;29(11):2218–32. doi: 10.1038/s41418-022-01009-9 (PMC9613881; doi:10.1038/s41418-022-01009-9)
Supplement: Supplementary file 1 — Supplementary figure legends [file 41418_2022_1009_MOESM1_ESM.docx]

**Figure S1** *Gene modified cells used in this study*

**a-j** Protein levels in CRISPR/Cas9 modified cells. Protein levels were detected with Western blotting.

**k** Verification of NOD1-deletion in AGS cells by sequence analysis indicated a frame-shift mutation in NOD1 in near proximity to the gRNA sequence.

**l** Detection of Smac reconstitution and EGFP expression in AGS cells by Western blotting.

**m-n** AGS cell lines (**h**: Bax^-/-^Bak^-/-^ clone 1; **i**: Smac^-/-^1 and Smac^-/-^2) were treated with 0.5 µM of the Smac-mimetic LCL-161 for 24 hours. Processing of p100 to p52 was detected by Western blotting. Shown are representative Western blots of two independent experiments. Smac 1 and Smac 2 refers to two different gRNAs.

**o-q** AGS (**j**) and HeLa (**k-l**) cell lines were treated with 0.5 nM, 1 nM (**j**) or 10 nM (**k-l**) PMA for 5 hours (**k-l**) or 24 hours (**j**). IL-8 in the supernatants was measured by ELISA in three independent experiments.

**Data information:** Bars represent the mean and dots the value of independent experiments. Error bars show standard error of mean. Ns: p>0.05. Significance was tested by parametric one-way ANOVA with Sidak´s post hoc test. CTRL, non-targeting control gRNA; Bax^-/-^Bak^-/-^, double deficiency of Bax and Bak; Bcl-X_L_, cells overexpressing Bcl-X_L_; CAD^-/-^, deletion of CAD by CRISPR/Cas9; Smac^-/-^, deletion of Smac by CRISPR/Cas9; TIFA^-/-^, deletion of TIFA by CRISPR/Cas9; IKKα^-/-^, deletion of IKKα by CRISPR/Cas9; Smac tg, Smac^-/-^  stably expressing Smac; EGFP, Smac^-/-^ stably expressing EGFP; Cas.9^-/-^, deletion of caspase-9 by CRISPR/Cas9; Cas.3^-/-^, deletion of caspase-3 by CRISPR/Cas9; Cas.7^-/-^, deletion of caspase-7 by CRISPR/Cas9.

**Figure S2** *Absence of detectable caspase-activation and cell death upon* H. pylori *infection of HeLa cells.*

**a** Caspase-activity in different cell lines (AGS, KatoIII: staining for active caspase-3; HeLa: FRET-reporter cells). Cells were left uninfected or infected with *H. pylori* (AGS, MOI=100; KATO III, MOI=10; HeLa, MOI=100) and analyzed by flow cytometry. Staurosporine (right panel) served as positive control. Note that the positive signal in the right panel is represented by a FRET-loss, i.e. movement to the left. Histograms are representative of at least three separate experiments.

**b** AGS cells were infected with *H. pylori* G27 strain at an MOI of 100 for 18 hours in the presence or absence of QVD (20 µM). Cell survival was measured by Trypan blue exclusion (left panel) or LDH release assay (middle panel). Effector caspase activity was measured in cell lysates with an AC-DEVD-AMC substrate (right panel). Data are from three independent experiments.

**c** HeLa cell lines carrying a reporter for active caspase-3 (Brokatzky et al., 2019) were infected with different MOI of *H. pylori* G27 or T26695 strains for 18 hours. Active caspase-3 was measured as FRET-loss by flow cytometry in three independent experiments for each *H. pylori* strain. Staurosporine was used as a positive control. R., reporter cells treated with staurosporine, c., control cells (without reporter).

**d** HeLa cell lines were infected with different MOI of *H. pylori* G27 and T26695 strains for 18 hours. Effector caspase activity was measured in cell lysates with an Ac-DEVD-AMC reporter substrate. Data are from three independent experiments for each *H. pylori* strain. Staurosporine was used as positive control.

**e** HeLa cells (CTRL) were infected with *H. pylori* G27 strain at an MOI of 100 for 18 hours. After 15 h, biotinylated VAD-fmk was added. Active effector caspases were precipitated from cell lysates using biotinylated VAD-fmk and neutravidin beads. Precipitated caspase-3 was detected by Western blotting. Shown is a representative Western blot taken from two independent experiments. Staurosporine was used as positive control.

**f** HeLa cell lines were infected with different MOI of *H. pylori* G27 strain for 48 hours. Staurosporine was added after 24 hours. Epithelial cell survival was measured by LDH release assay. Cytotoxicity was calculated as described in Methods. Staurosporine was used as positive control. Data are from three independent experiments

**g** HeLa cell lines were infected with different MOI of *H. pylori* G27 for 18 hours. Loss of mitochondrial membrane potential (mmp) was analyzed by flow cytometry after staining with a mitochondrial membrane potential dependent mitotracker. Shown are representative histograms and a quantification of five independent experiments. Staurosporine control was performed two times.

**Data information:** Bars represent the mean and dots the value of independent experiments. Error bars show standard error of mean. Ns: p>0.05. Significance was tested by parametric (**c**) and non-parametric (**d**;**f:** Dunn´s post hoc test) one-way ANOVA. CTRL/CT., non-targeting control gRNA; Bax^-/-^Bak^-/-^/BB, cells double-deficient in Bax and Bak. I, input; B, bound to beads; U, unbound.

**Figure S3** *Time-course of* *Caspase-3 activation and loss of cell membrane integrity upon* H. pylori *infection in AGS cells*

**a, b** AGS cell lines were infected with *H. pylori G27* strain at an MOI of 100 for different time periods. Cell survival was measured by Trypan blue exclusion (**a**) or by LDH release assay (**b**) in three independent experiments.

**c** AGS cell lines were infected with *H. pylori G27* strain at an MOI of 100 for different time periods. Effector caspase activity was measured in cell lysates with an AC-DEVD-AMC reporter substrate in three independent experiments.

**Data information:** Bars represent the mean and dots the values of independent experiments. Error bars show standard error of mean. CTRL, non-targeting control gRNA; Bax^-/-^Bak^-/-^, cells double-deficient in Bax and Bak.

**Figure S4** H. pylori *induces a Smac dependent activation of the alternative NFκB pathway.*

**a** AGS cell lines were infected with *H. pylori G27* strain at an MOI of 100 for 18 hours. Endogenous levels of Smac (green) and cytochrome *c* (red) were detected by co-staining and immunofluorescence. Shown are individual fluorescence channels and magnified pictures of Fig.3D. Brightness and contrast were adjusted after magnification. Scale bars indicate 10 µM. Asterisk indicates a cell showing selective loss of Smac. Arrows indicate mitochondrial regions with a loss of Smac.

**b** AGS cell lines (control, Bax or Bak single-deficient or Bax/Bak double-deficient) were infected with *H. pylori* G27 strain at an MOI of 100 for 18 hours. Endogenous Smac and cytochrome *c* were detected by immunofluorescence as in S3A. Smac and cytochrome *c* fluorescence intensity were quantified by calculating the corrected total cell fluorescence (CTCF) in the same cells. Shown are three independent experiments and the means. Significance was calculated with the mean value of each individual experiment.

**c** KATOIII cell lines (control or Bax/Bak-deficient) were infected with *H. pylori* G27 strain at an MOI of 10 for 5 hours. Endogenous Smac (green) and cytochrome *c* (red) were detected by immunofluorescence. Shown are the zoomed-in pictures from Fig.3F. Brightness and contrast were adjusted after magnification. Scale bar: 2µm

**d** AGS and KATOIII cells were infected with *H. pylori* G27 strain at an MOI of 100 for 18 hours (AGS) and an MOI of 10 for 5 hours (KATOIII). Mitochondria mass was analysed by flow cytometry after staining for the mitochondrial protein Hsp60. Shown is a quantification of the mean fluorescence intensity (MFI) of three independent experiments.

**e** AGS and KATOIII cells (control or Bax/Bak-deficient) were infected with *H. pylori* G27 strain at an MOI of 100 for 18 hours (AGS) and an MOI of 10 for 5 hours (KATOIII). Mitochondria membrane potential was analysed by flow cytometry after staining with a mitochondrial membrane potential dependent mitotracker. Shown is a quantification of the mean fluorescence intensity (MFI) of three independent experiments.

**f** AGS cells (control or Nod1-deficient) were infected with *H. pylori* G27 at an MOI of 100 for 24 hours. IL-8 was measured by ELISA in three independent experiments.

**g** HeLa cells expressing Smac-GFP were infected with *H. pylori* G27 at an MOI of 100 for 18 hours. Smac-GFP (green) and endogenous cytochrome *c* (red) were detected by fluorescence microscopy. Shown are representative pictures and the quantification of four independent experiments with at least 180 cells per condition. Scale bars indicate 50 µm.

**h** HeLa cell lines were infected with *H. pylori* G27 strain at an MOI of 100 for 18 hours. Endogenous levels of Smac and cytochrome *c* from whole cell lysates were detected by Western blotting. Shown are representative Western blots and quantification of five independent experiments. Three outliers were removed after Grubbs outlier testing. Not normalized data are shown in S8L.

**i** HeLa cells expressing Smac-GFP were infected with *H. pylori* G27 at an MOI of 100 in combination with staurosporine (0,25 µM) and/or Z-VADfmk (50 µM). Smac-GFP (green) and endogenous cytochrome *c* (red) were detected by fluorescence microscopy. Scale bars indicate 50 µm.

**j** AGS cells were infected with *H. pylori* G27 at an MOI of 100 in combination with a proteasome inhibitor (MG-132, 5µM) for 6 hours. Endogenous Smac (green) and endogenous cytochrome *c* (red) were detected by fluorescence microscopy. Shown are representative pictures of three independent experiments with at least 75 cells per condition. 54% of MG-132-treated cells and 11.62% of untreated, *Hp*-infected cells showed presence of cytosolic Smac after *H. pylori* and MG-132 treatment. Scale bars indicate 5 µm.

**Data information:** Bars represent mean and dots value of independent experiments. Error bars show standard error of mean. NS: p>0.05, *, p<0.05, **, p<0.01, ***, p<0.001. Significance was tested by unpaired T-Test (**f,g**), one-sample T-Test (**h**) and parametric one-way-ANOVA with Sidak´s post hoc test (**b, d, e**) . CTRL, non-targeting control gRNA; Bax^-/-^Bak^-/-^, double deletion of Bax and Bak by CRISPR/Cas9; Bak^-/-^, deletion of Bak by CRISPR/Cas9; Bax^-/-^, deletion of Bax by CRISPR/Cas9; Smac^-/-^, deletion of Smac by CRISPR/Cas9.

**Figure S5**

**a-b** HeLa (**a**) and AGS (**b:** Smac^-/-^ gRNA 1) cell lines were infected with *H. pylori* G27 strain at an MOI of 100 for 18 hours (**a**) or 24 hours (**b**). Processing of NFκB p100 to p52 was detected by Western blotting. Shown are representative Western blots and quantification from three independent experiments.

**c** HeLa cell lines were infected with *H. pylori* G27 strain at an MOI of 100 for 18 hours. Phosphorylation of NFκB p65 was detected by Western blot as indicator of the activation of classical NF-κB. Shown is a representative Western blot and a quantification of five independent experiments.

**d** HeLa cells expressing a reporter for NF-κB were infected with *H. pylori* G27 strain at an MOI of 100 for 18 hours. EGFP expression is induced after NF-κB activation and was measured by flow cytometry. Shown is a quantification of the mean fluorescence intensity (MFI) of six independent experiments.

**e** KATOIII cell lines were infected with *H. pylori* G27 strain at an MOI of 10 for 5 hours. XIAP was detected by Western blot. Shown is a representative Western blot and a quantification of four independent experiments.

**Data information:** Bars represent mean and dots value of independent experiments. Error bars show standard error of mean. NS: p>0.05, *, p<0.05, **, p<0.01, ***, p<0.001. Significance was tested by parametric one-way-ANOVA with Sidak´s post hoc test (a), paired T-Test (d), or unpaired T-Test (c,e). CTRL, non-targeting control gRNA; Bax^-/-^Bak^-/-^, double deletion of Bax and Bak by CRISPR/Cas9; Smac^-/-^, deletion of Smac by CRISPR/Cas9

**Figure S6** *H. pylori* causes a DNA-damage response through the mitochondrial apoptosis pathway

**a-b** AGS cell lines (Bax^-/-^Bak^-/-^ clone 1) were infected with *H. pylori* T26695 strain at an MOI of 5 (**b**) or *H. pylori* G27 strain at an MOI of 30 (**a**) for 18 hours. The DNA-damage response was measured as γH2AX-signal by Western blot. Signal intensity of γH2AX was measured and normalized to GAPDH.

**c-d** HeLa cell lines were infected with *H. pylori* G27 strain at an MOI of 5 (**c**) or *H. pylori* T26695 strain at different MOI (**d**) for 18 hours. The caspase inhibitor Z-VADfmk was added with the bacteria. The DNA-damage response was measured as γH2AX-signal by Western blotting. Shown are representative Western blots and quantification (**c**) of at least two independent experiments. Not normalized data are shown in S8M.

**Data information:** Bars represent the mean and dots the value of independent experiments. Error bars show standard error of mean. CTRL, non-targeting control gRNA; Bax^-/-^Bak^-/-^, cells double deficient in Bax and Bak; CAD^-/-^, deletion of CAD by CRISPR/Cas9.

**Figure S7** H. pylori *activates the mitochondrial apoptosis system through its type 4 secretion system and host NOD1 but independent of CagA.*

**a-b** AGS cell lines were infected at an MOI of 100 using various *H. pylori* deletion strains for 22 hours (**a,** T26695 strain; **b,** G27 strain). The DNA-damage response was measured as γH2AX-signal by Western blotting. Shown are representative Western blots and quantification of at least three independent experiments.

**c-d** HeLa cell lines were infected at an MOI of 100 using various *H. pylori* deletion strains for 22 hours (**c,** G27 strain; **d,** T26695 strain). Processing of NF-κB p100 to p52 and the DNA-damage response (γH2AX-signal) were measured by Western blotting. Shown are representative Western blots of two (**c**) and three (**d**, including quantification) independent experiments.

**e** HeLa cell lines were infected with *H. pylori G27* strain at an MOI of 100 for 18 hours. Endogenous levels of Smac (green) and cytochrome *c* (red) were detected by immunofluorescence. Shown are magnified pictures of Fig. **6a**. Brightness and contrast were adjusted after magnification. Scale bars indicate 20 µm. Asterisk indicates a cell with selective loss of Smac. Arrows indicate mitochondrial regions with a loss of Smac.

**f** .HeLa cell lines were infected with *H. pylori G27* strain at an MOI of 100 for 18 hours. The NOD1-inhibitor ML-130 was added at various concentrations at the time of *H. pylori* infection. Processing of NF-κB p100 to p52 and endogenous level of Smac in whole cell lysates were measured by Western blotting. Shown are one representative Western blot and the quantification of five independent experiments.

**g** AGS cell lines were infected with *H. pylori G27* at an MOI of 100 for 18 hours. Endogenous level of Smac in whole cell lysates and the DNA-damage response by appearance of a γH2AX-signal were measured by Western blotting. Shown are representative Western blots of three individual experiments.

**h** Hela cell lines were infected at an MOI of 100 of *H. pylori* G27 strain for 18 hours. The endogenous level of Smac was measured in whole cell lysates by Western blotting. Shown are representative Western blots and the quantification of five independent experiments. One data point was removed after Grubbs outlier testing. Not normalized data are shown in S8O.

**Data information:** Bars represent the mean and dots the value of independent experiments. Error bars show standard error of mean. Ns: p>0.05, *, p<0.05, **, p<0.01, ***, p<0.001, ****, p<0.0001. Significance was tested by one-way ANOVA with Dunnett´s post hoc test (**a**,**d,f**), unpaired T-test (**b**) and one sample T-test (**h**). WT, wild type Helicobacter pylori; ΔCagA, CagA was deleted in *H.p.*; ΔPAI, deletion of the cag-pathogenicity island in *H.p.*; ΔBabA, deletion of BabA in *H.p.;* CTRL, non-targeting control; TIFA^-/-^, deletion of TIFA by CRISPR/Cas9.

**Fig. S8** *Original non-normalized data corresponding to normalized data in main and supplementary figures*

**a-k** Original data relating to normalized data in main figures 1-6

**l-n** Original data relating to normalized data in supplementary figures S4-S7
