# Supplementary figures and images for: Mitochondria supply sub-lethal signals for cytokine secretion and DNA-damage in H. pylori infection
Source: Cell Death Differ. 2022 May 3;29(11):2218–32. doi: 10.1038/s41418-022-01009-9 (PMC9613881; doi:10.1038/s41418-022-01009-9)

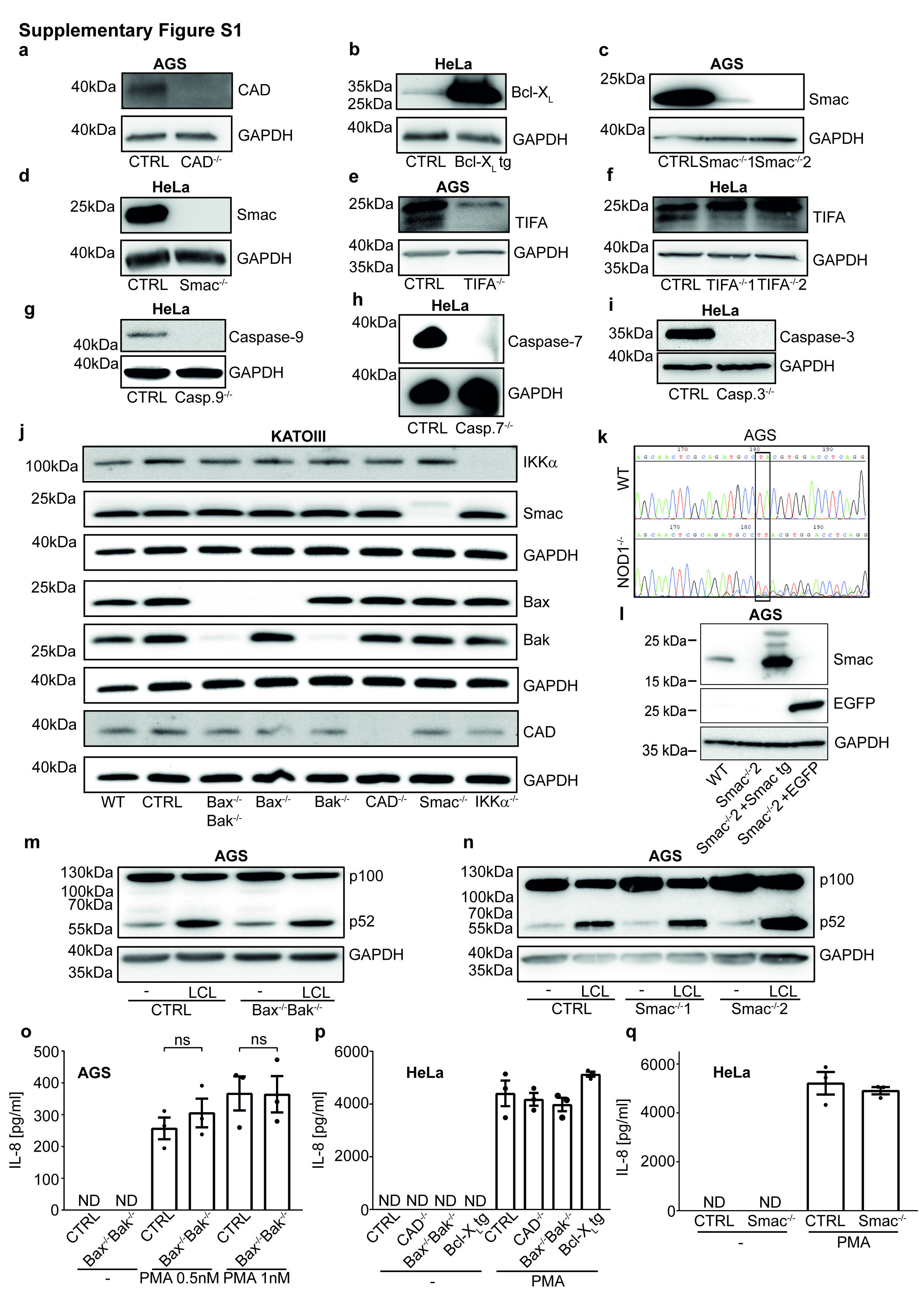

Supplement: Supplementary file 2 — Suppl. FigS1 [file 41418_2022_1009_MOESM2_ESM.tif]

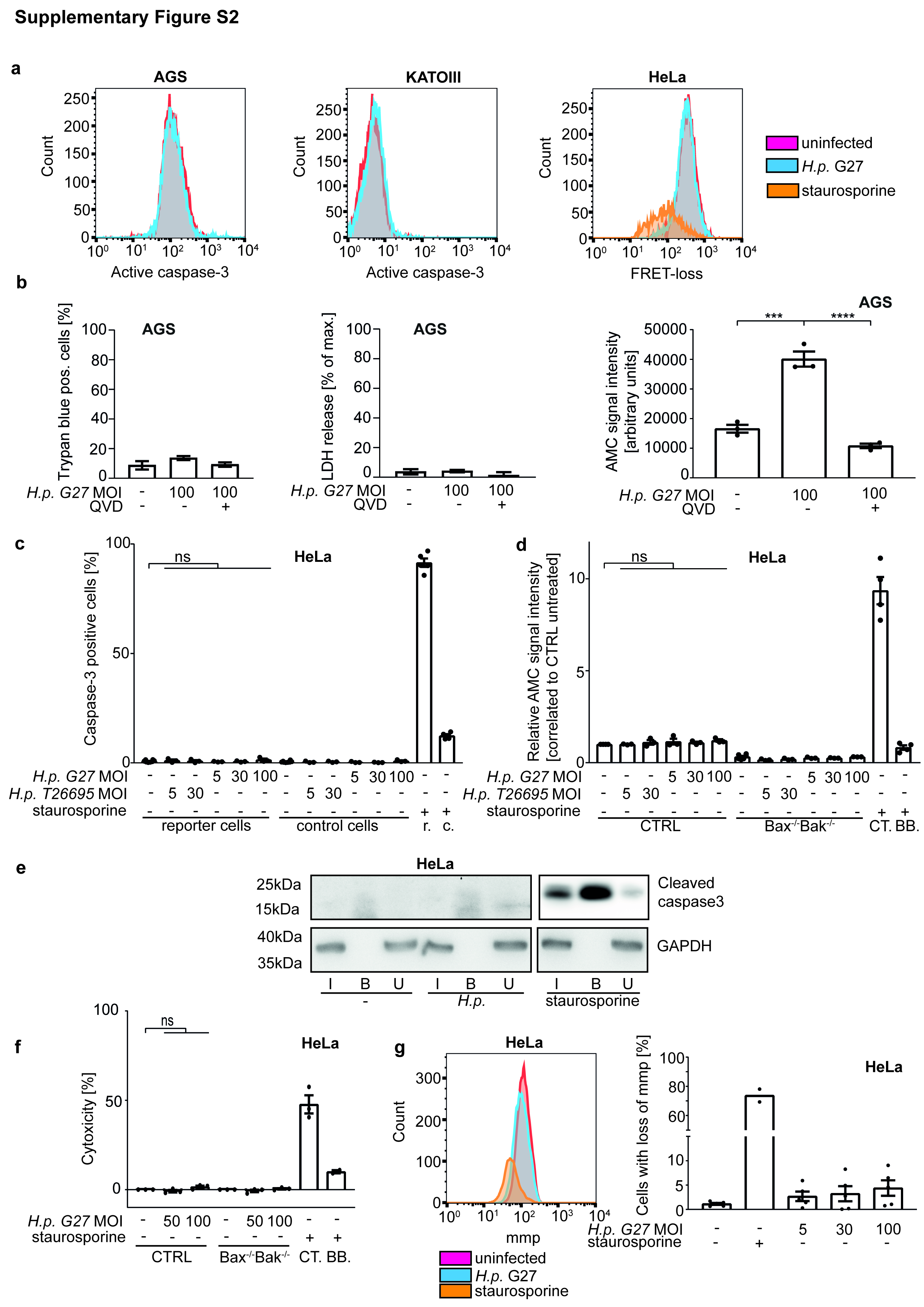

Supplement: Supplementary file 3 — Suppl. FigS2 [file 41418_2022_1009_MOESM3_ESM.tif]

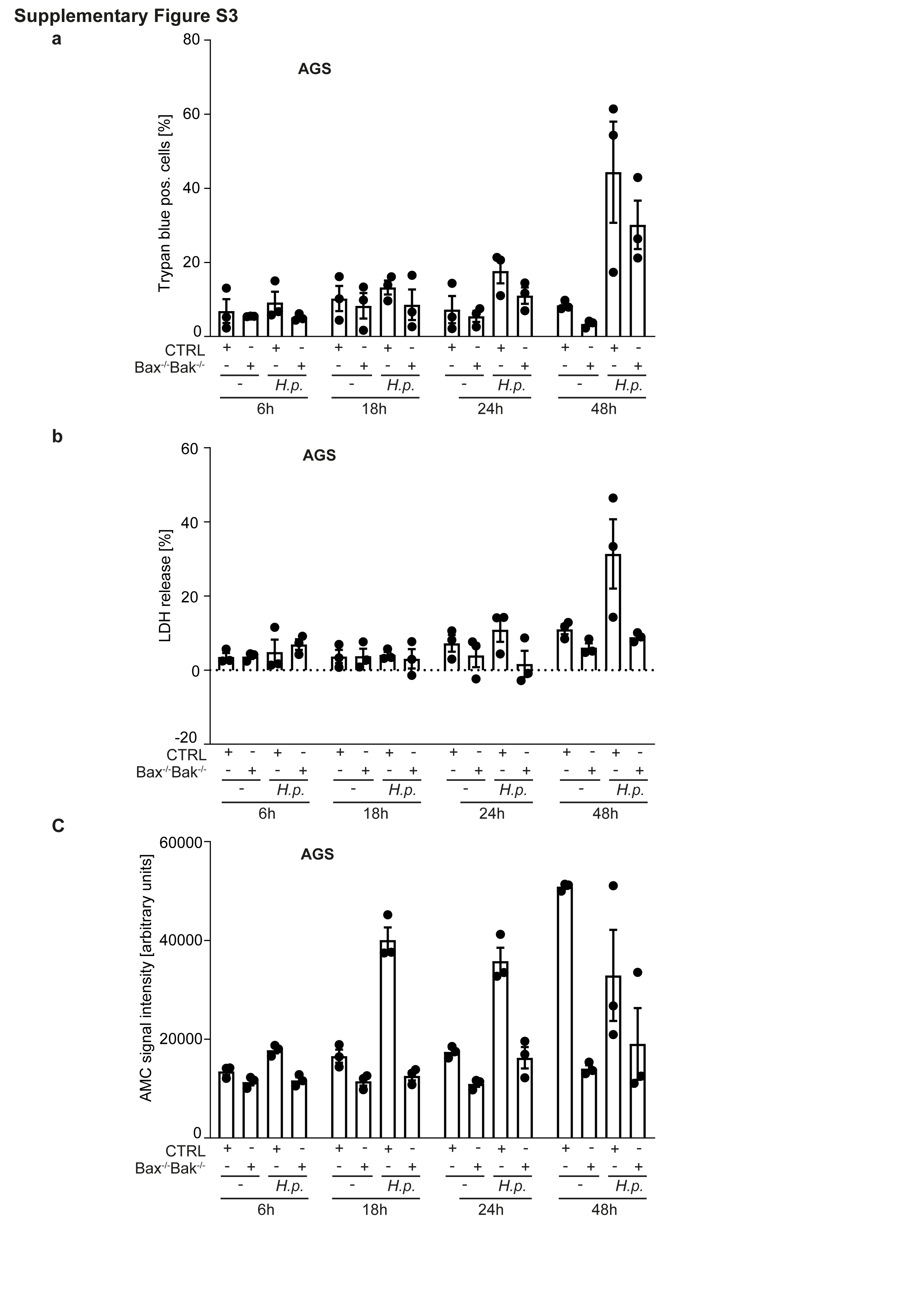

Supplement: Supplementary file 4 — Suppl. FigS3 [file 41418_2022_1009_MOESM4_ESM.tif]

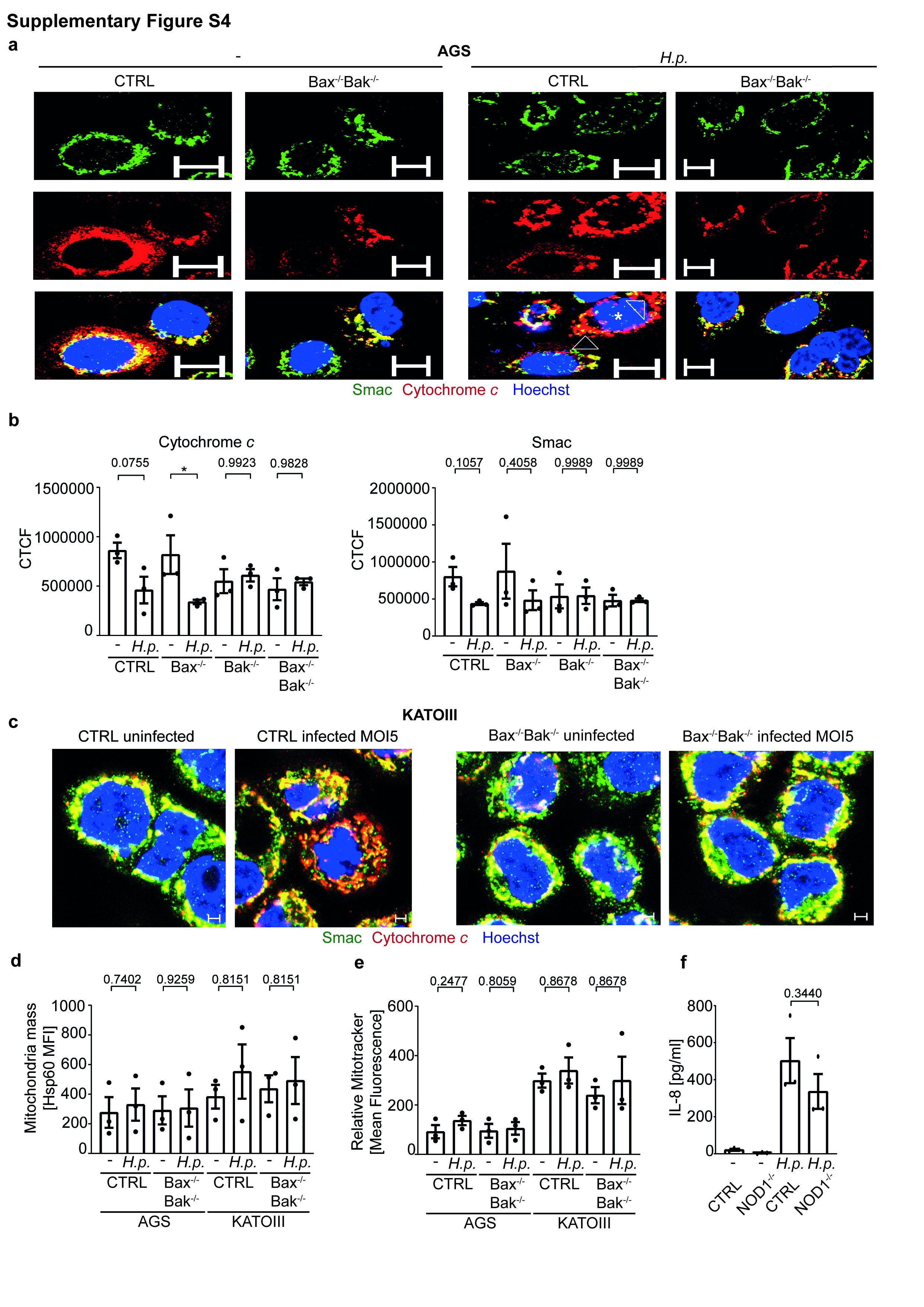

Supplement: Supplementary file 5 — Suppl. FigS4_part1 [file 41418_2022_1009_MOESM5_ESM.tif]

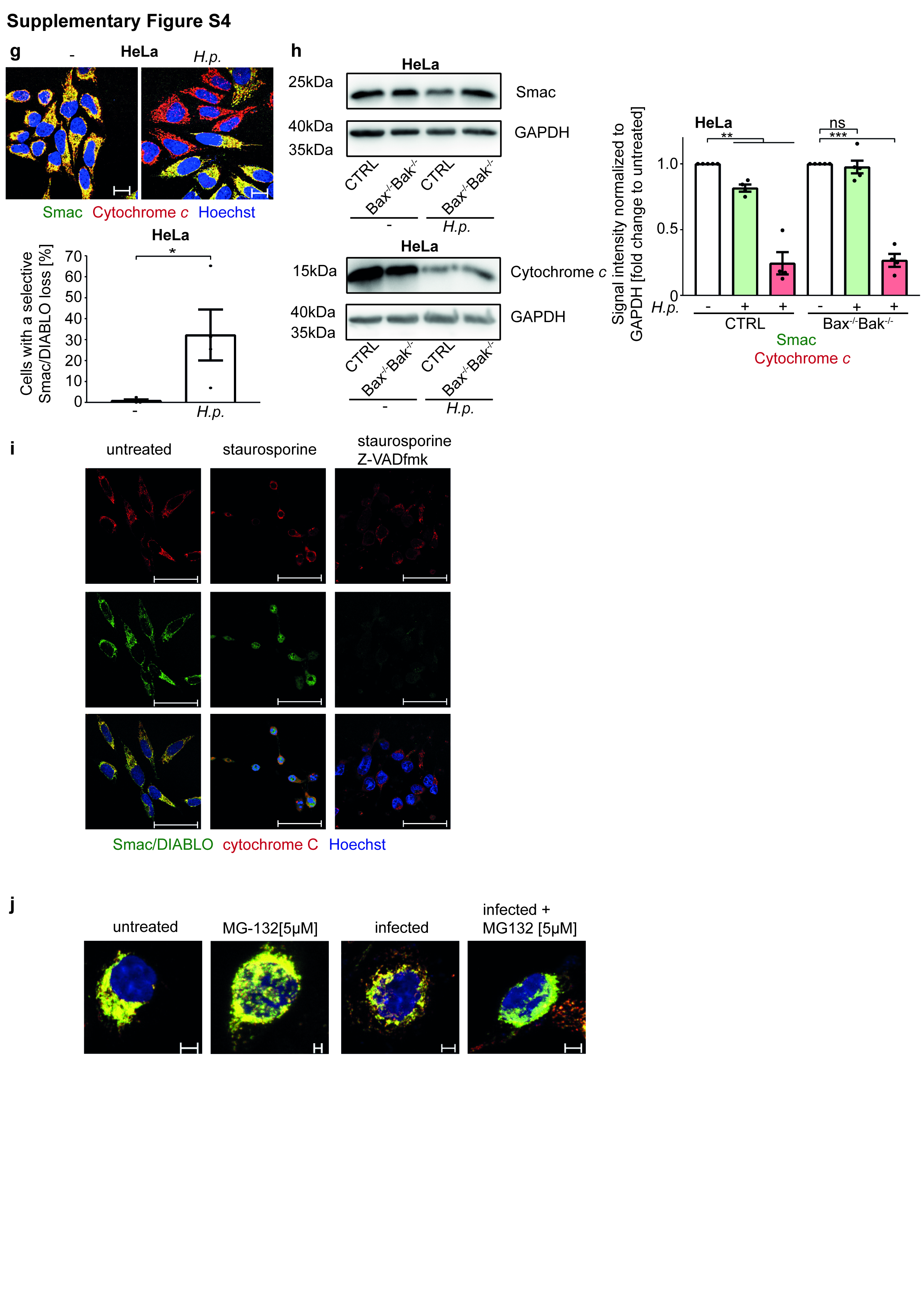

Supplement: Supplementary file 6 — Suppl. FigS4_part2 [file 41418_2022_1009_MOESM6_ESM.tif]

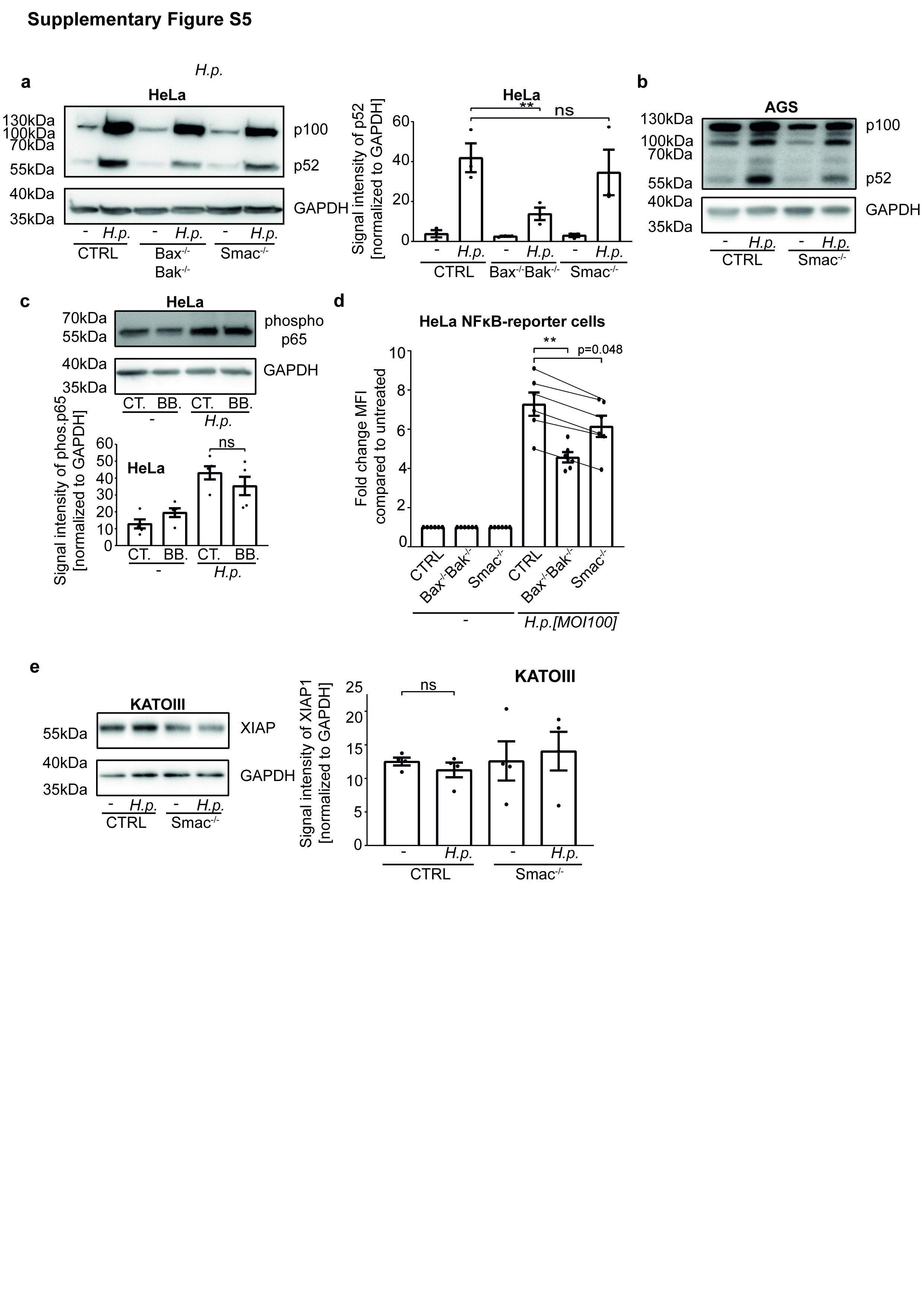

Supplement: Supplementary file 7 — Suppl. FigS5 [file 41418_2022_1009_MOESM7_ESM.tif]

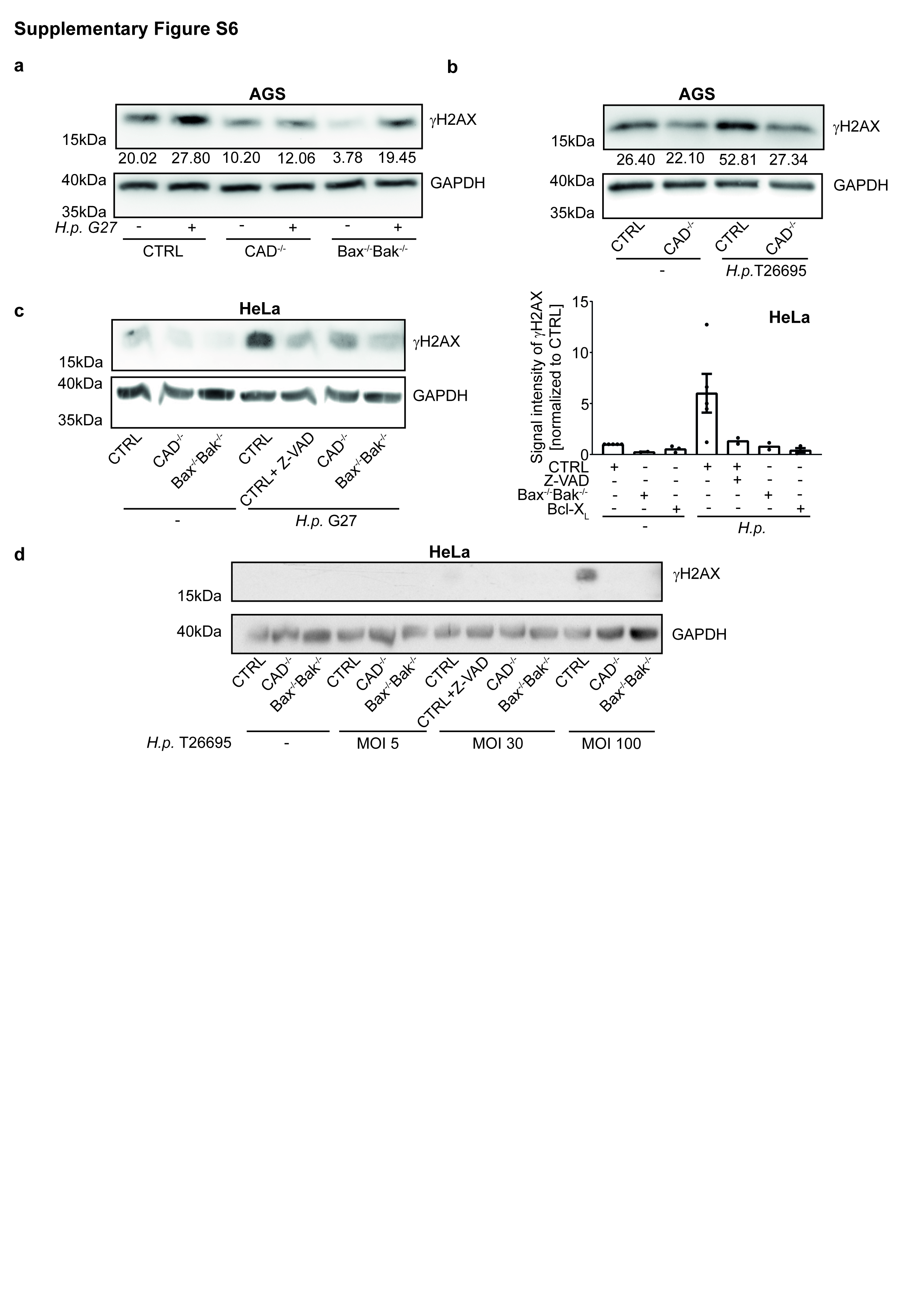

Supplement: Supplementary file 8 — Suppl. FigS6 [file 41418_2022_1009_MOESM8_ESM.tif]

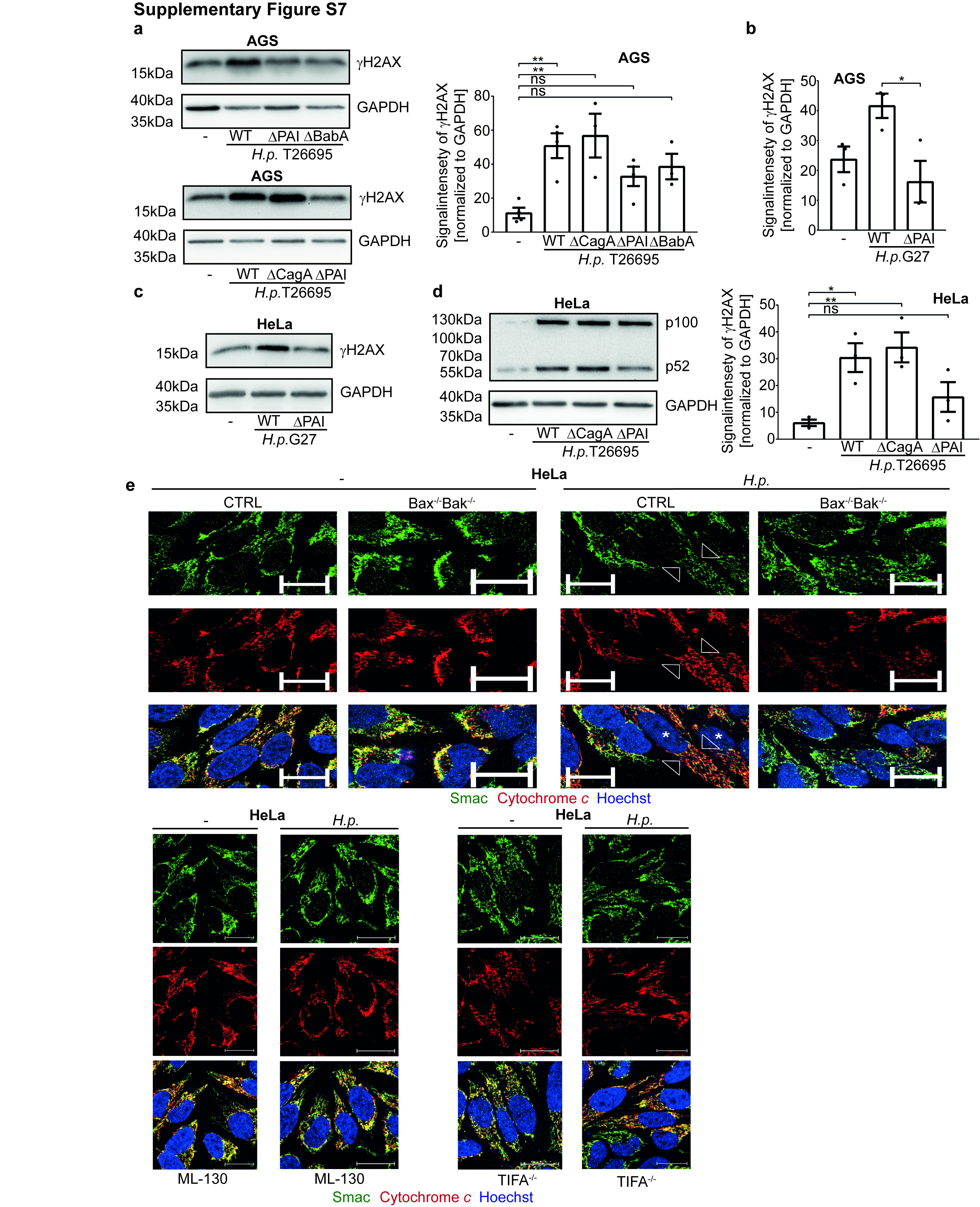

Supplement: Supplementary file 9 — Suppl. FigS7_part1 [file 41418_2022_1009_MOESM9_ESM.tif]

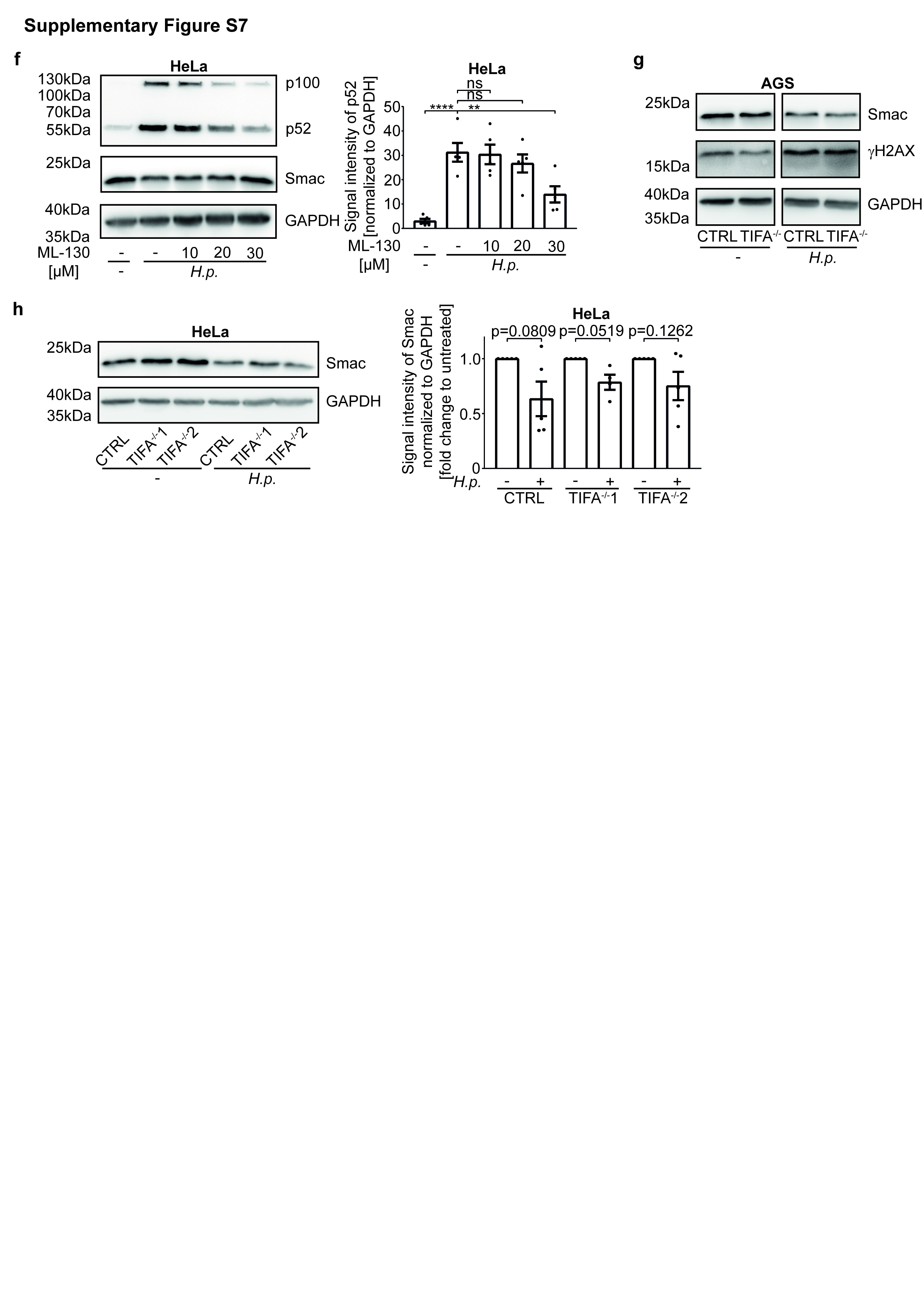

Supplement: Supplementary file 10 — Suppl. FigS7_part2 [file 41418_2022_1009_MOESM10_ESM.tif]

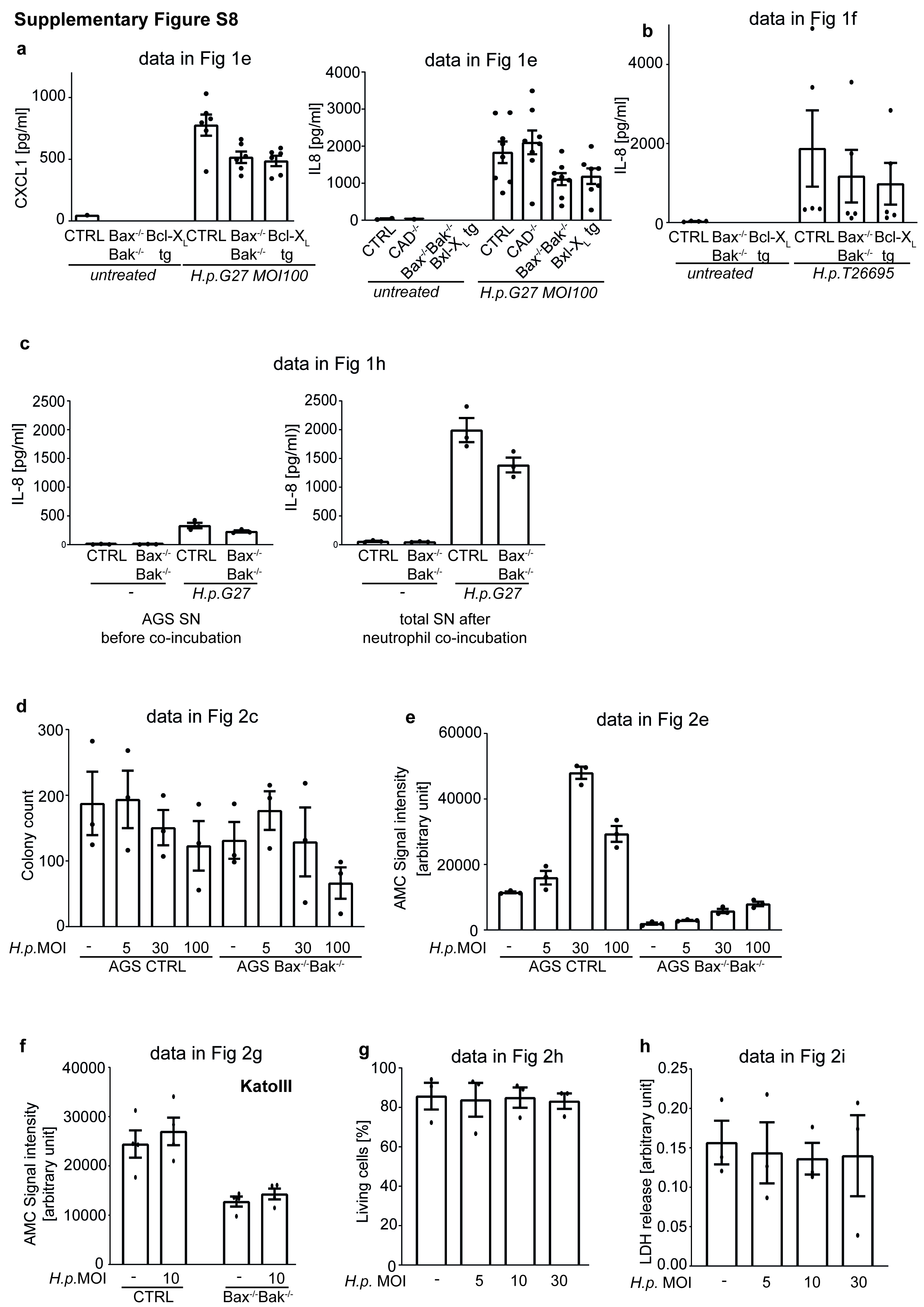

Supplement: Supplementary file 11 — Suppl. FigS8_part1 [file 41418_2022_1009_MOESM11_ESM.tif]

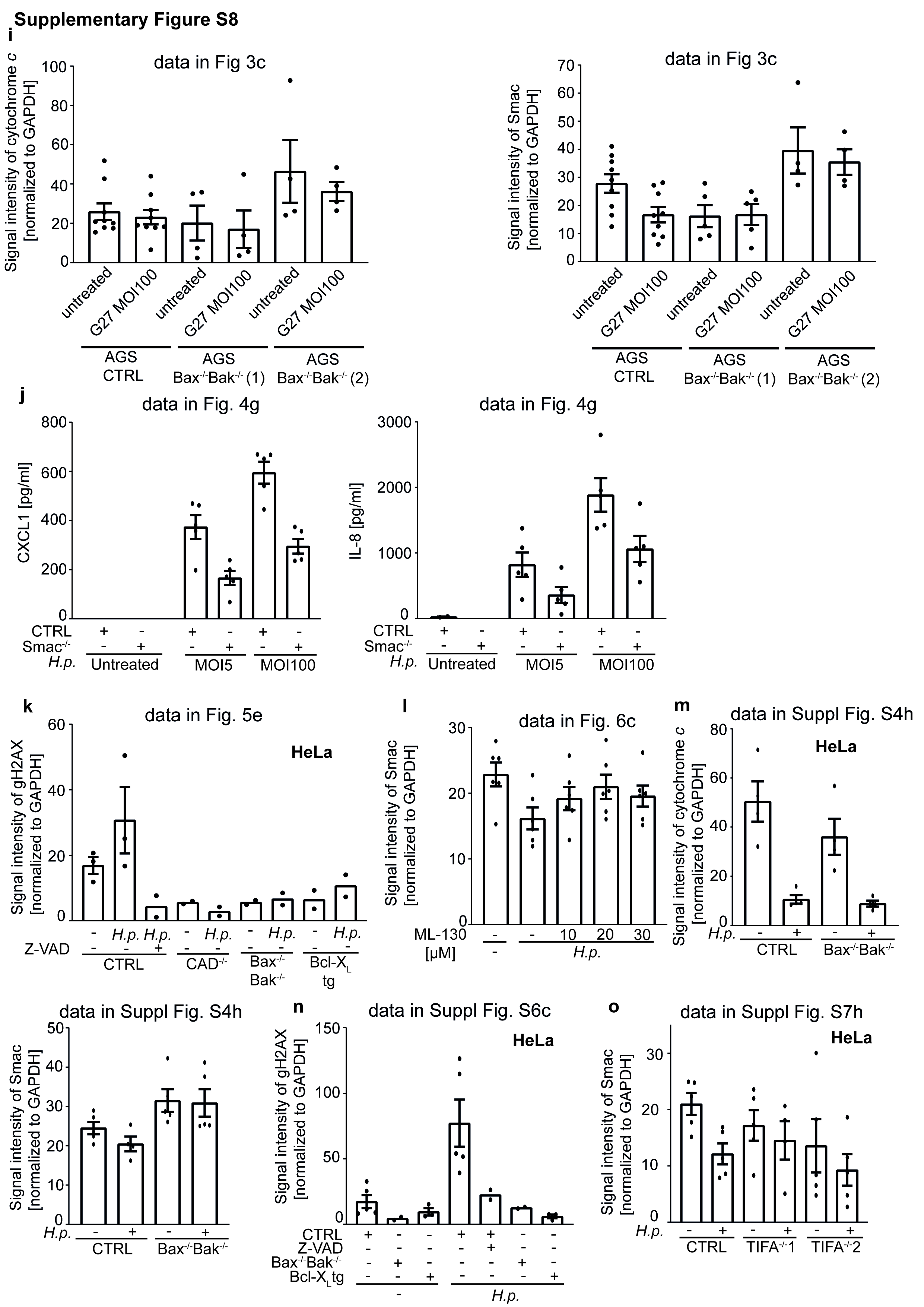

Supplement: Supplementary file 12 — Suppl. FigS8_part2 [file 41418_2022_1009_MOESM12_ESM.tif]
