# Supplementary material for: Mitochondria supply sub-lethal signals for cytokine secretion and DNA-damage in H. pylori infection
Source: Cell Death Differ. 2022 May 3;29(11):2218–32. doi: 10.1038/s41418-022-01009-9 (PMC9613881; doi:10.1038/s41418-022-01009-9)

Fig 2d

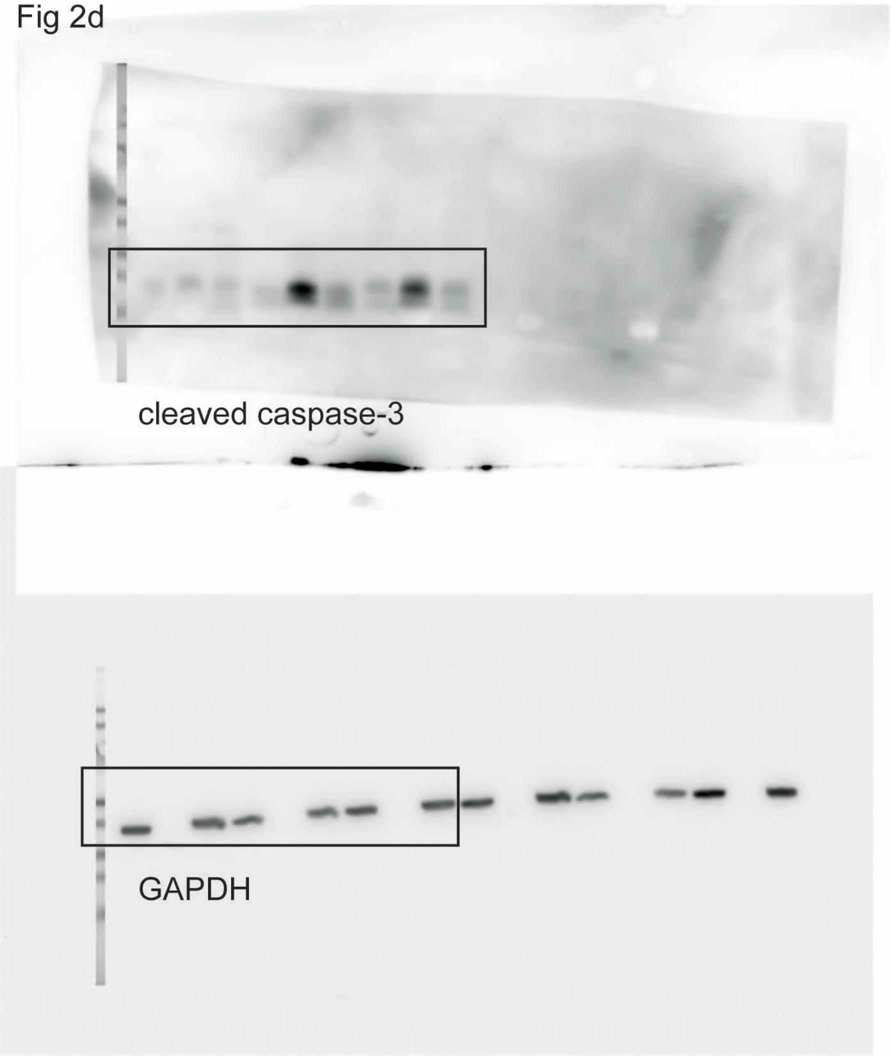

Fig 3a

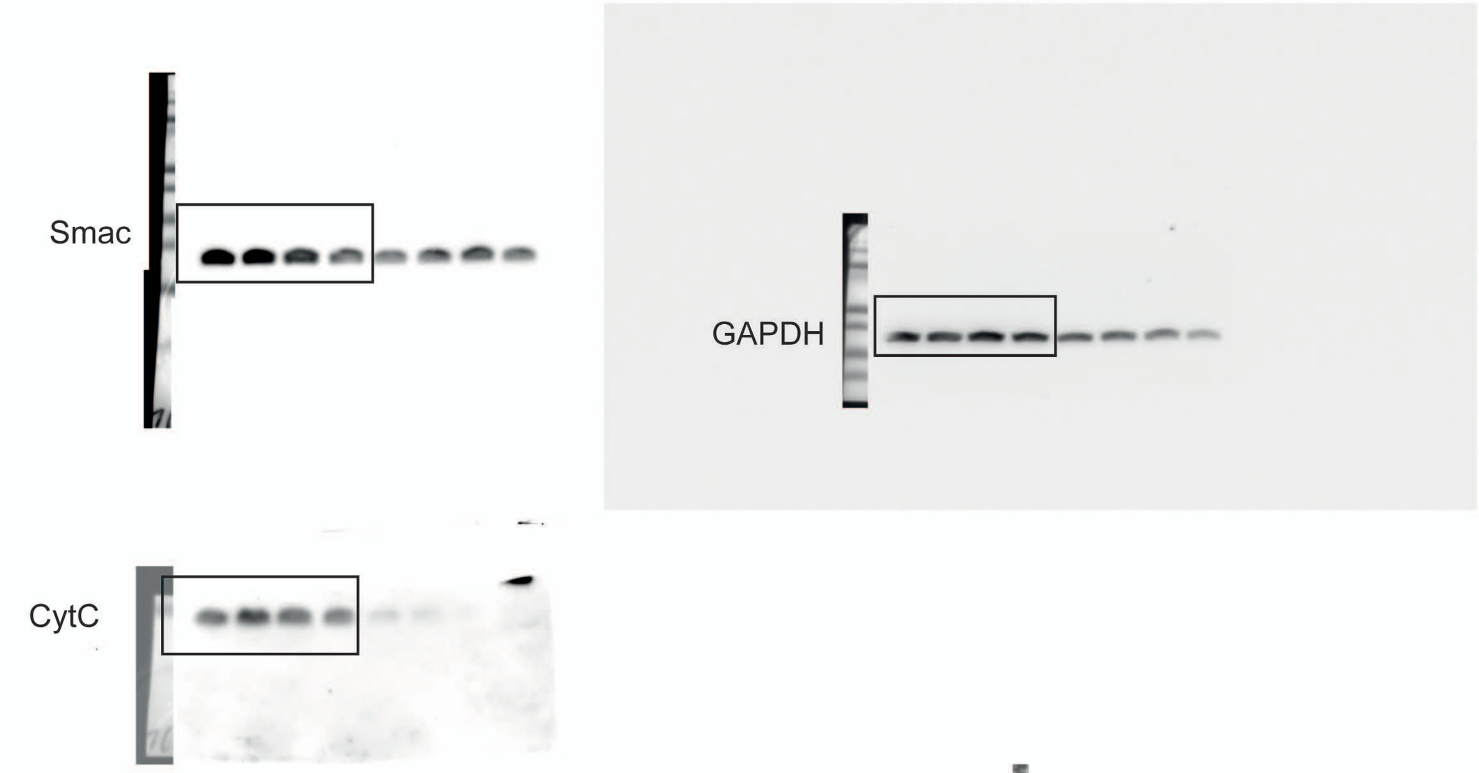

Fig 3b

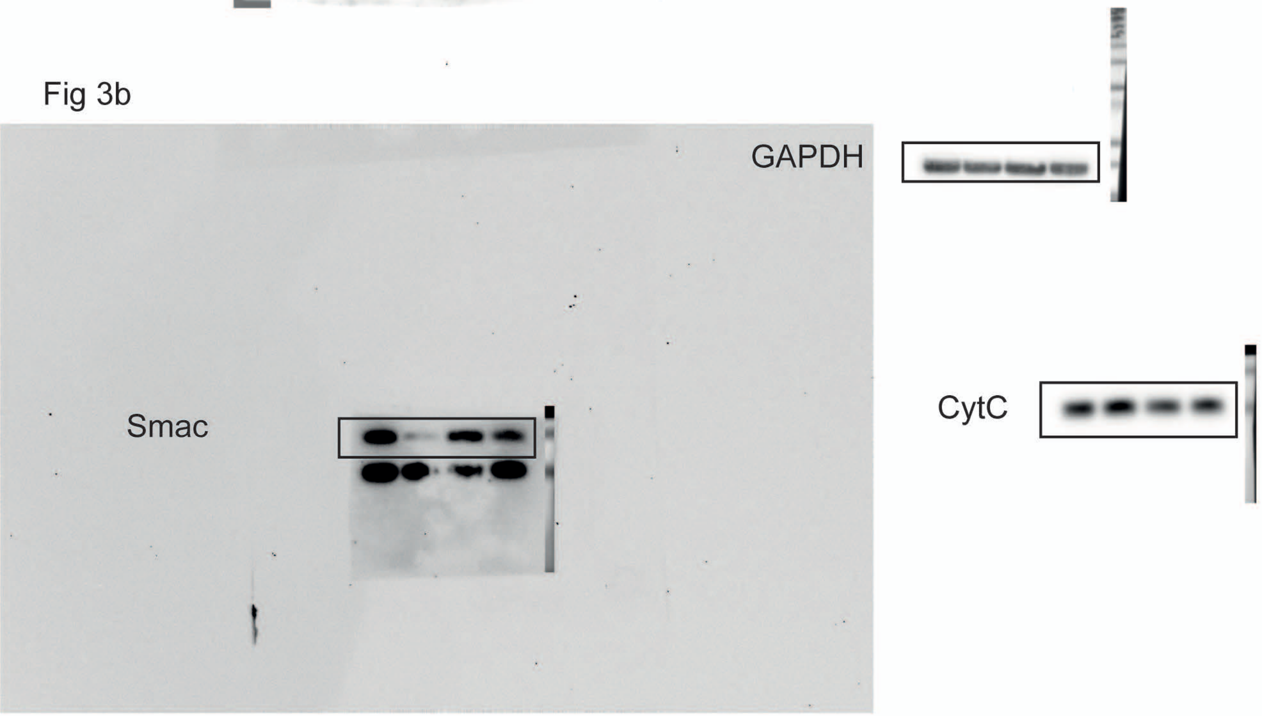

Fig 3e

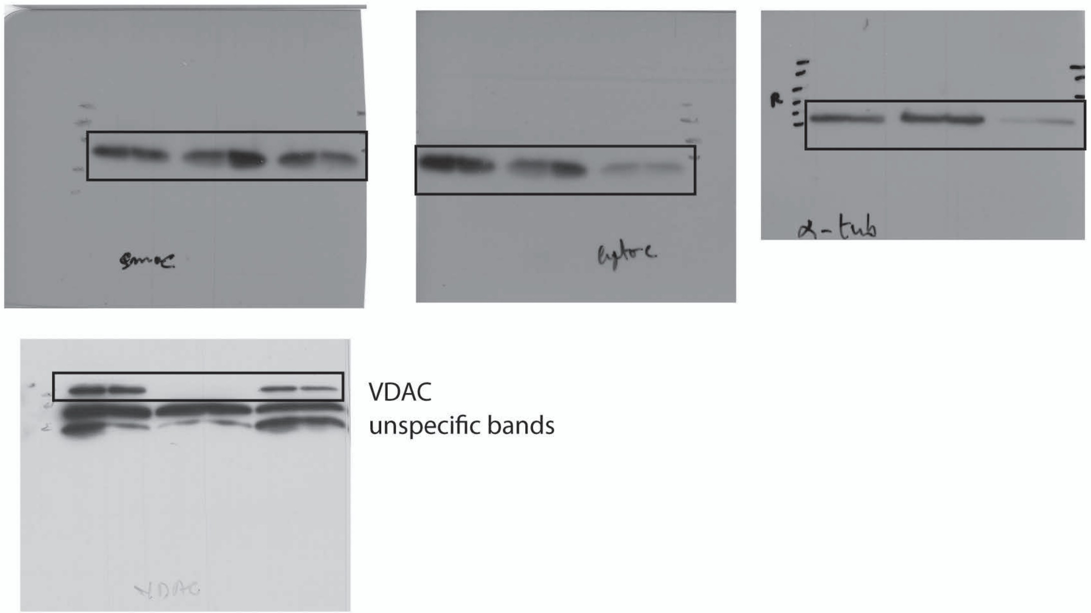

Fig 4a

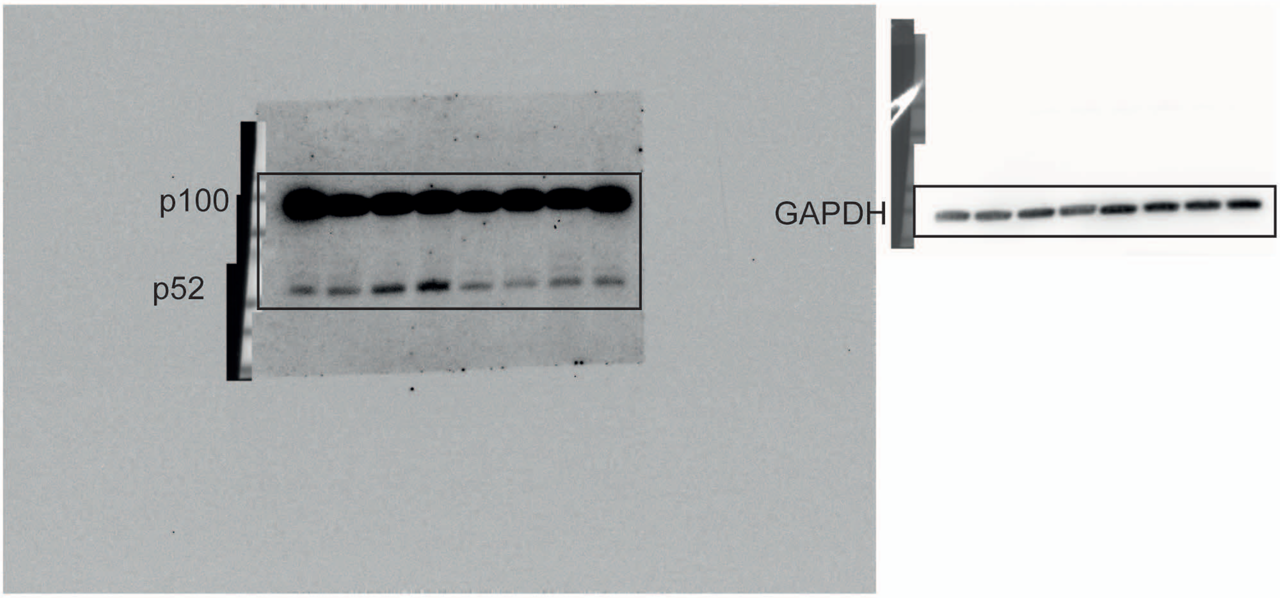

Fig 4b

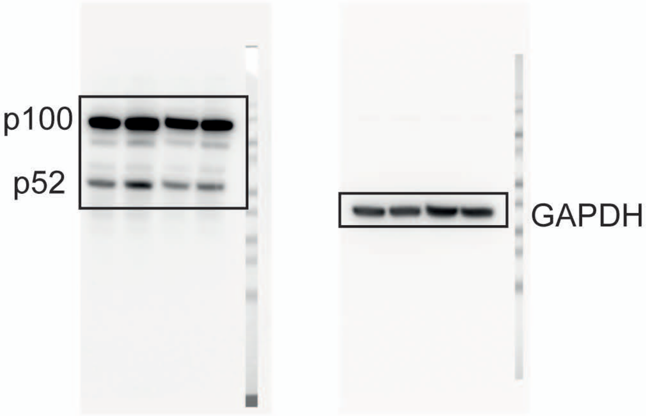

Fig 4c

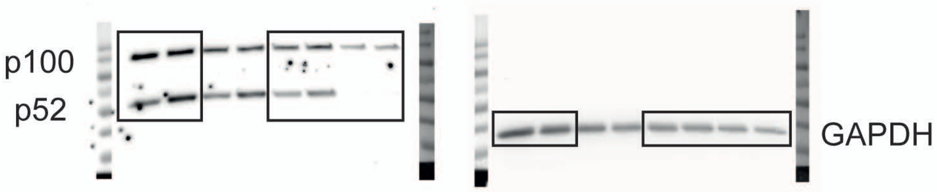

Fig 4d

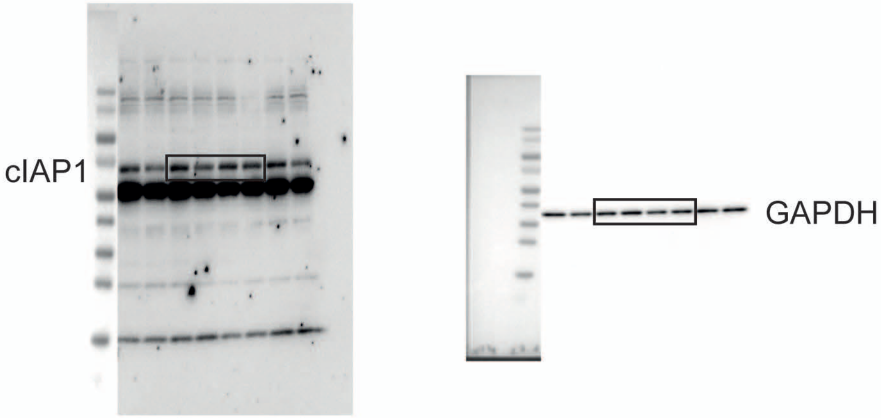

Fig 5c

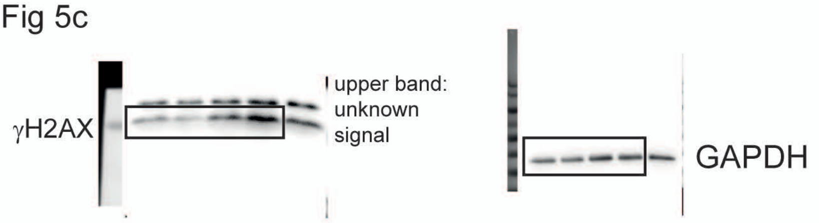

Fig 5d

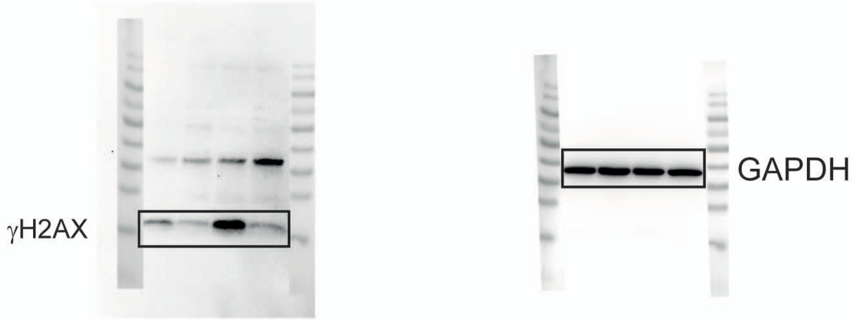

Fig 5e

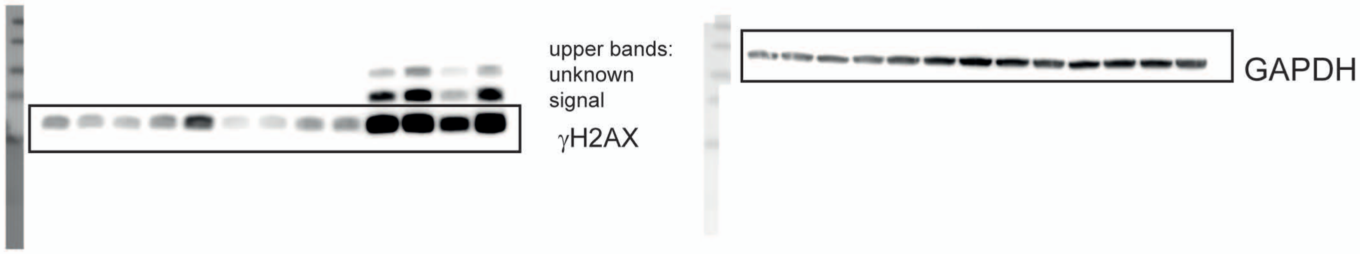

Fig 5f

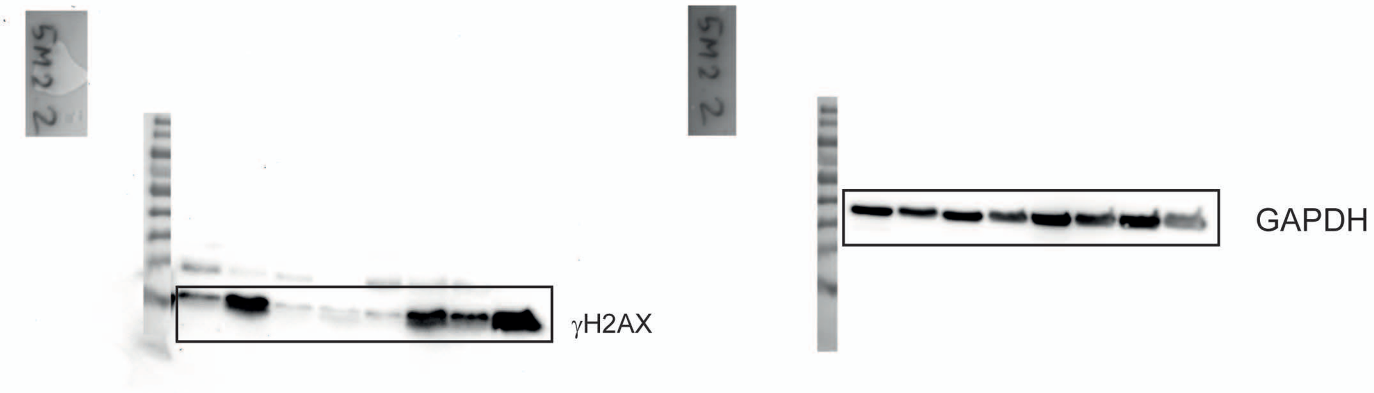

Fig 6b

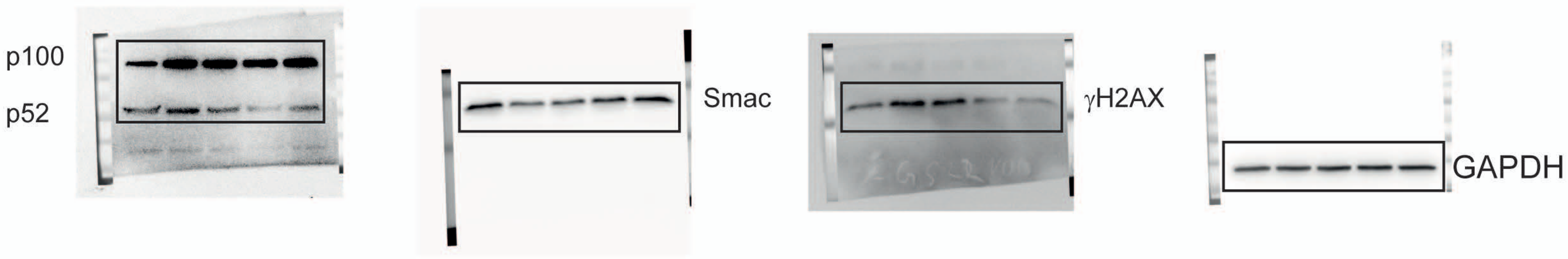

Fig 6d

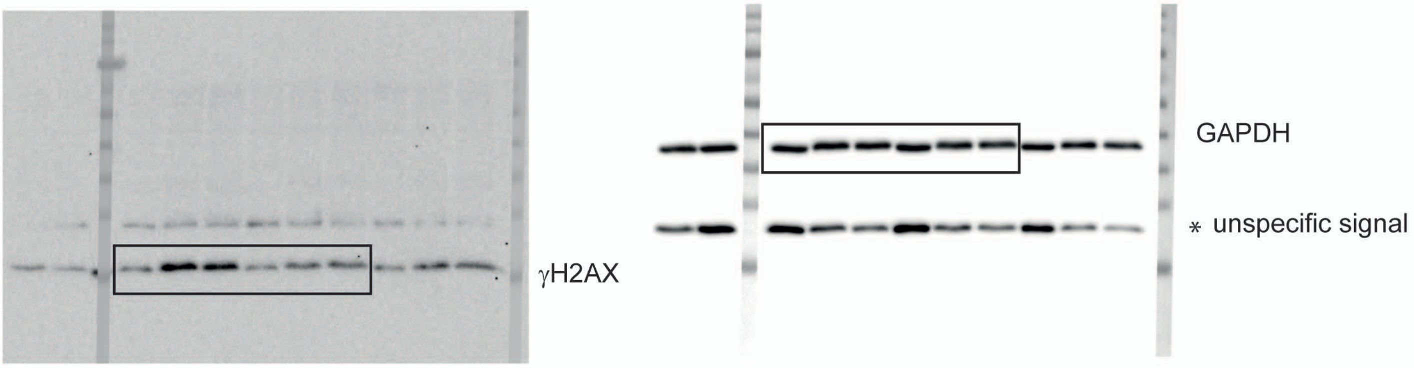

Fig 6e

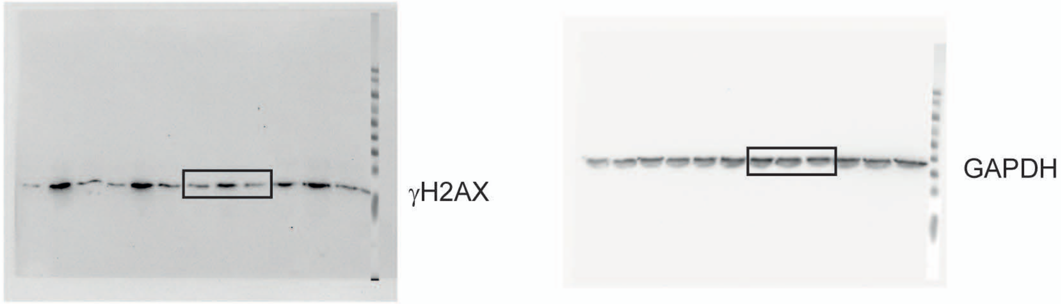

Suppl. Fig. S1a

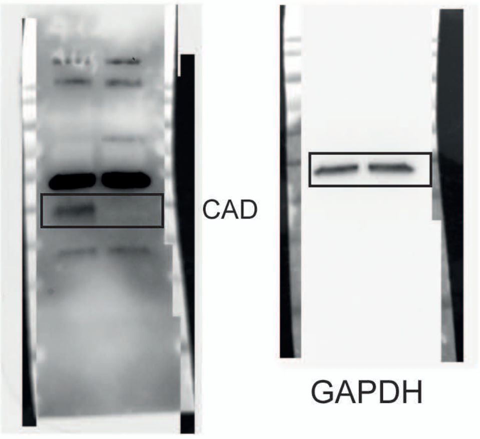

Suppl. Fig. S1b

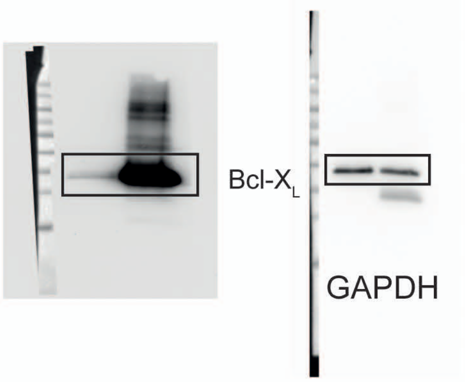

Suppl. Fig. S1c

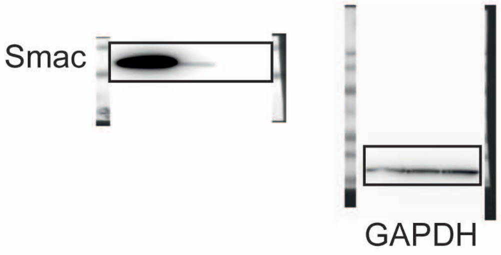

Suppl. Fig. S1d

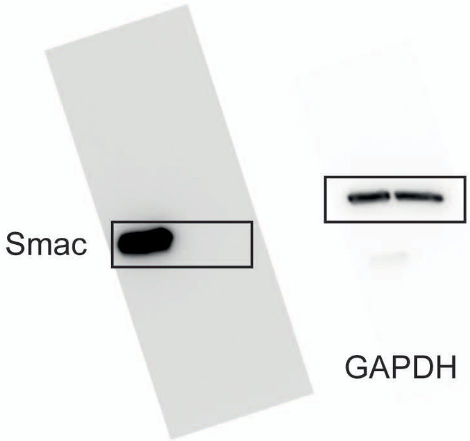

Suppl. Fig. S1e

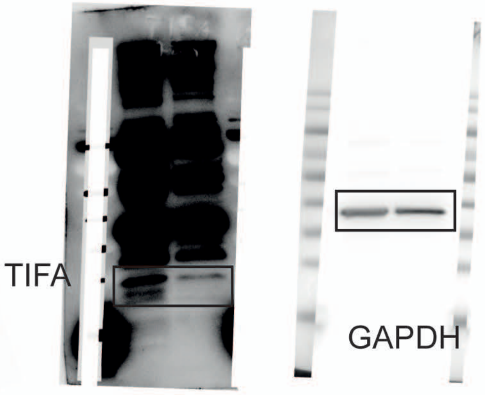

Suppl. Fig. S1f

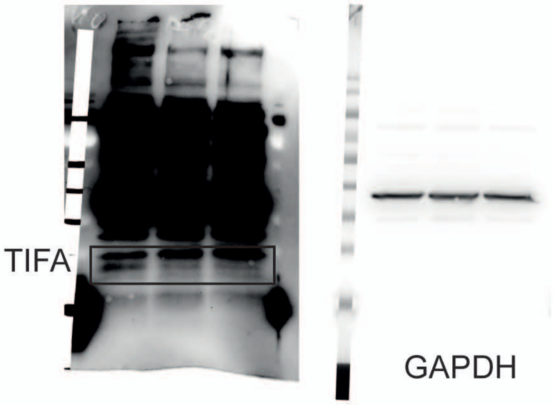

Suppl. Fig S1g

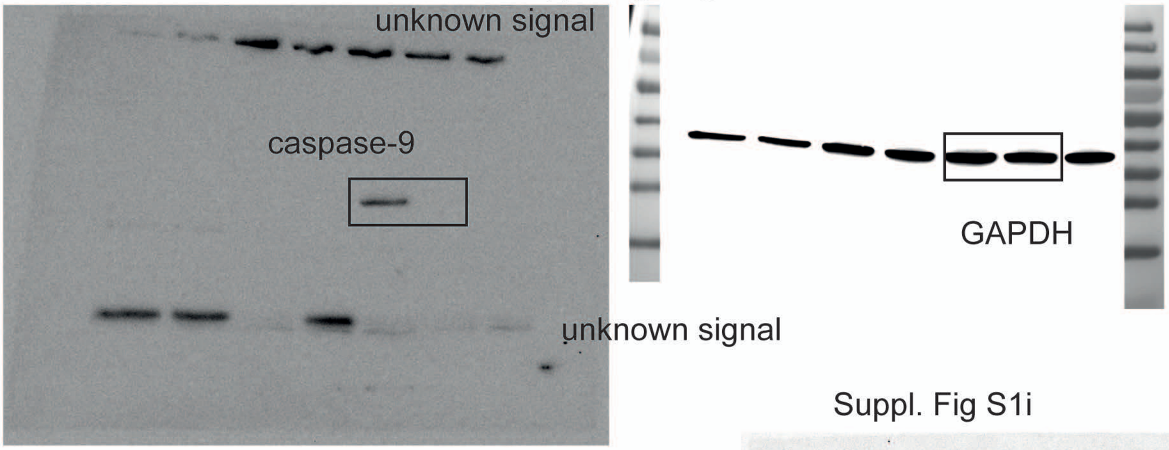

Suppl. Fig S1h

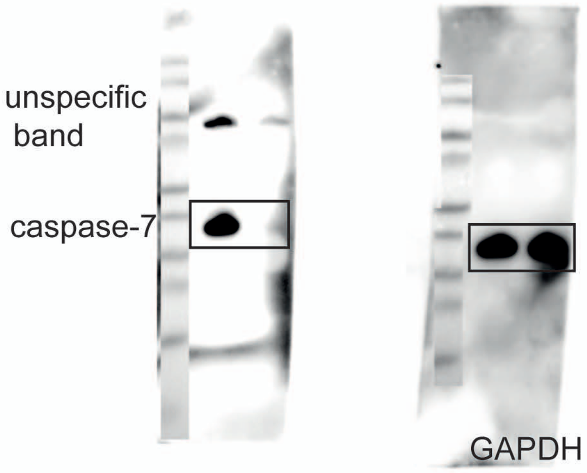

Suppl. Fig S1i

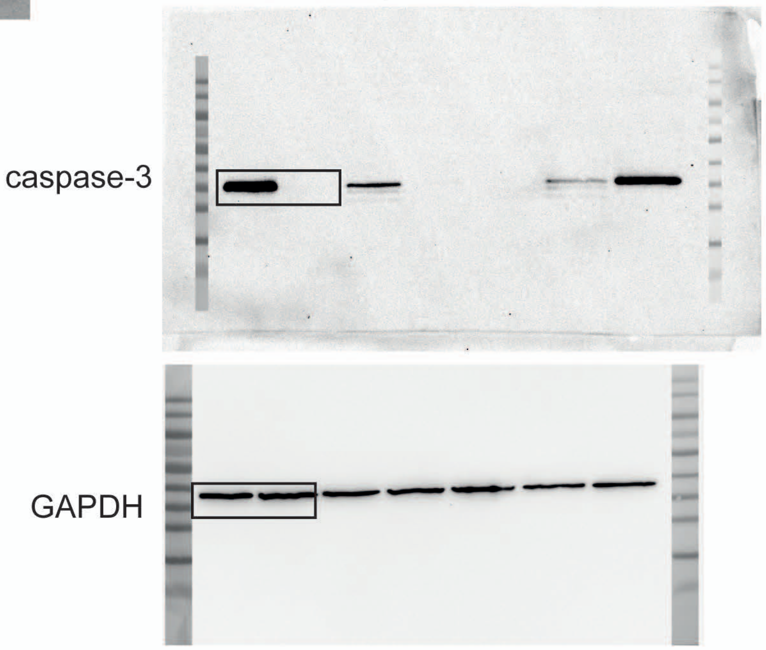

Suppl. Fig. S1j

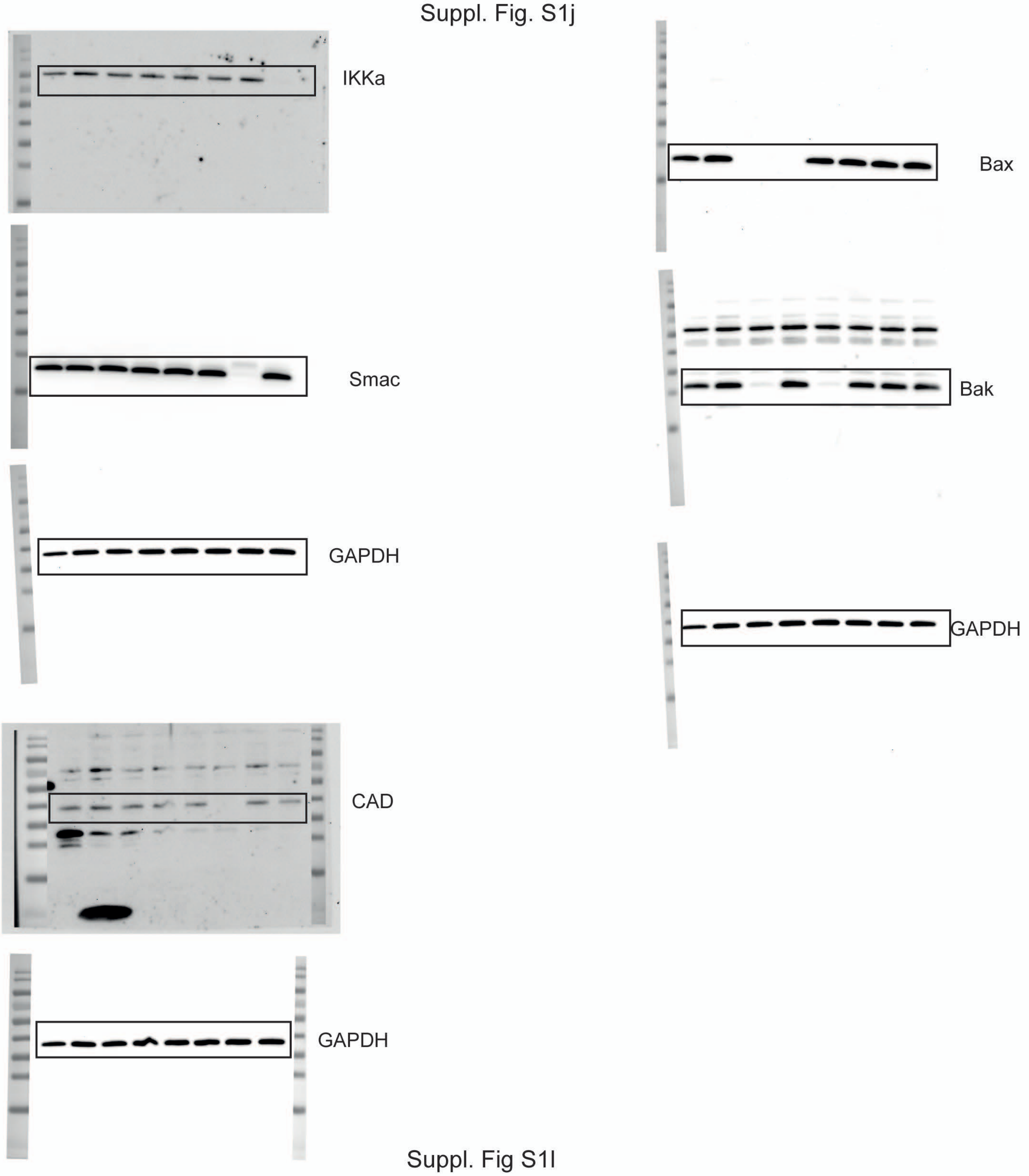

Suppl. Fig S1l

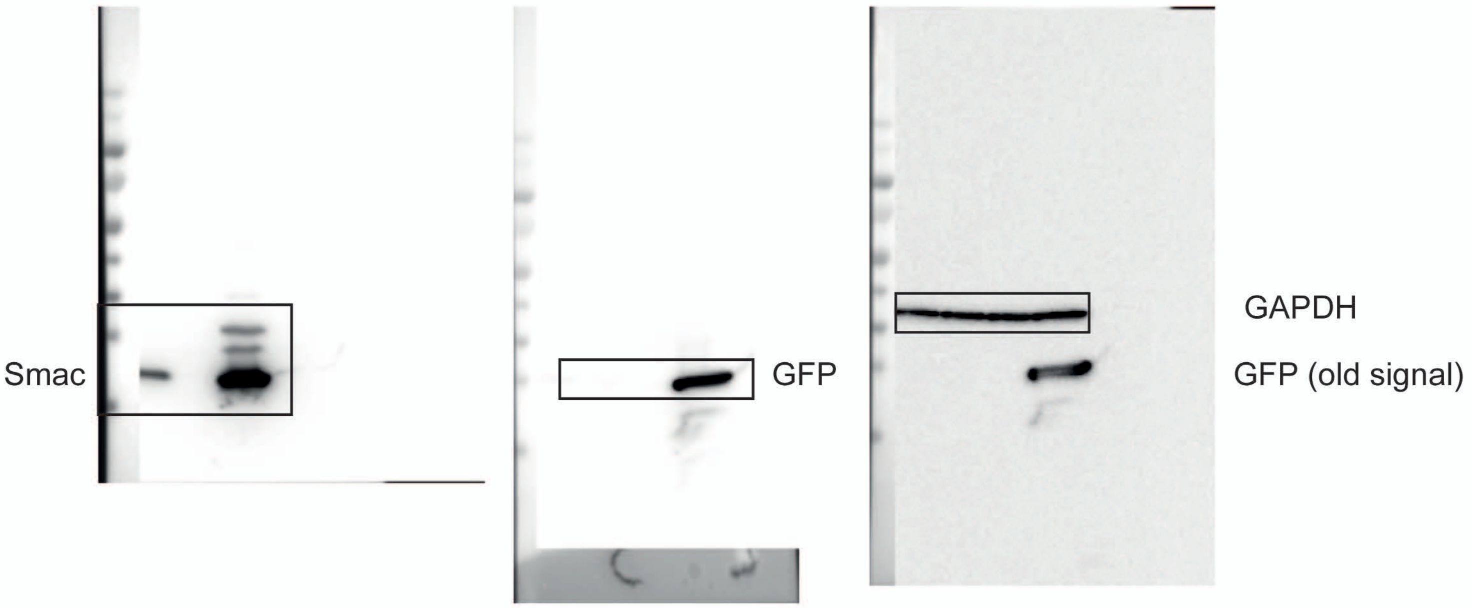

Suppl. Fig S1m

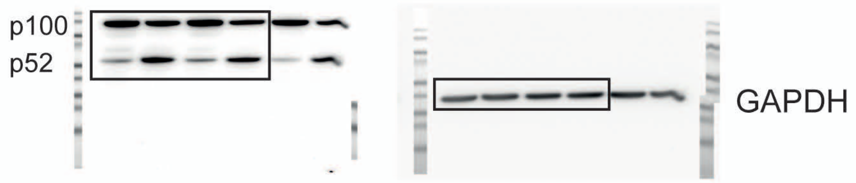

Suppl. Fig S1n

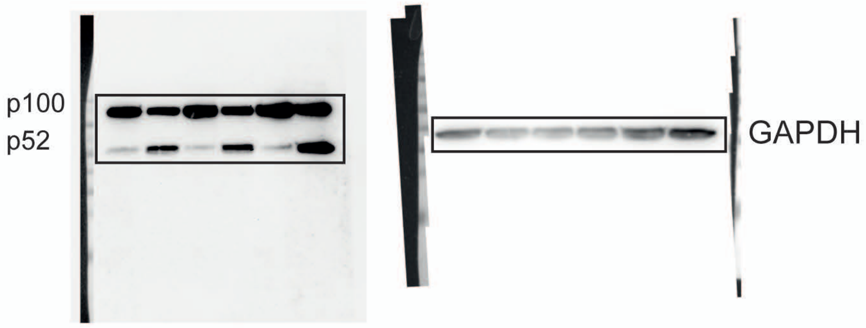

Suppl. Fig S2e

cleaved Caspase-3

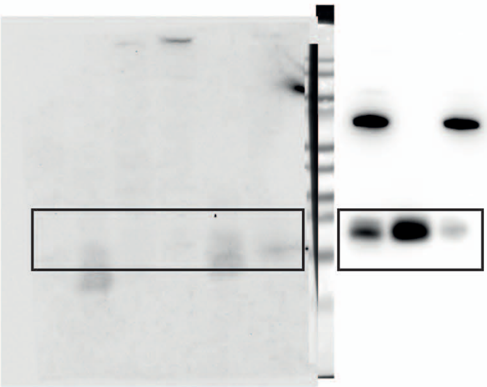

unknown signal

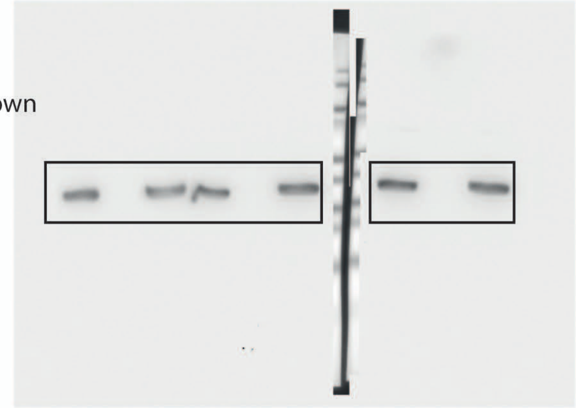

GAPDH

Suppl. Fig S4h

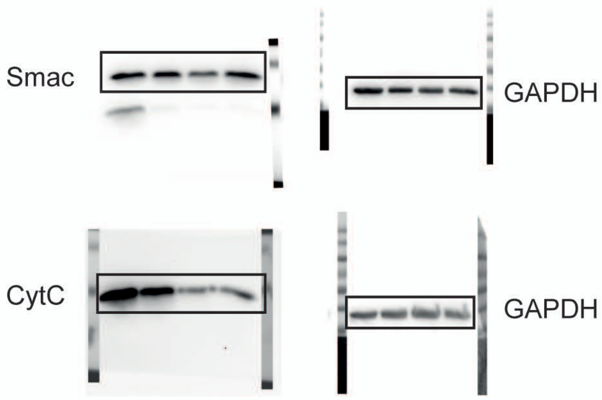

Suppl. Fig S5a

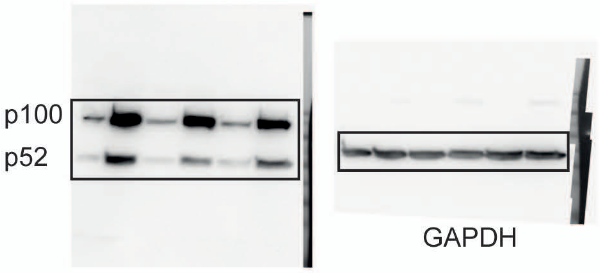

Suppl. Fig S5b

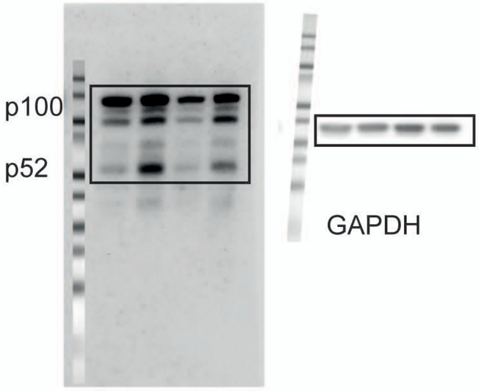

Suppl. Fig S5c

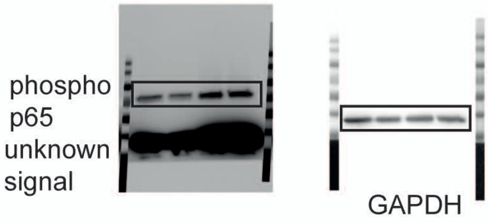

Suppl. Fig S5e

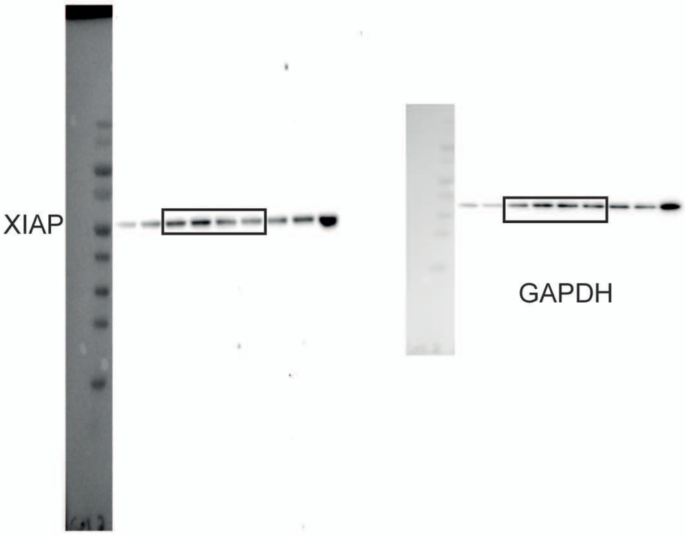

Suppl. Fig S6a

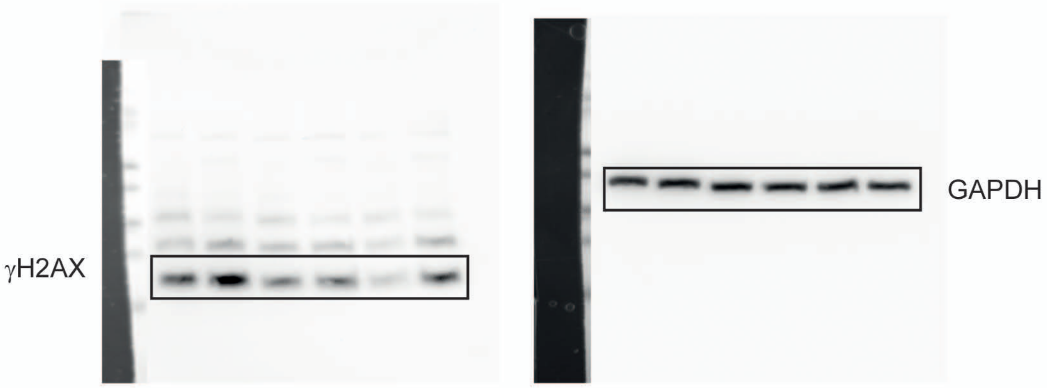

Suppl. Fig S6b

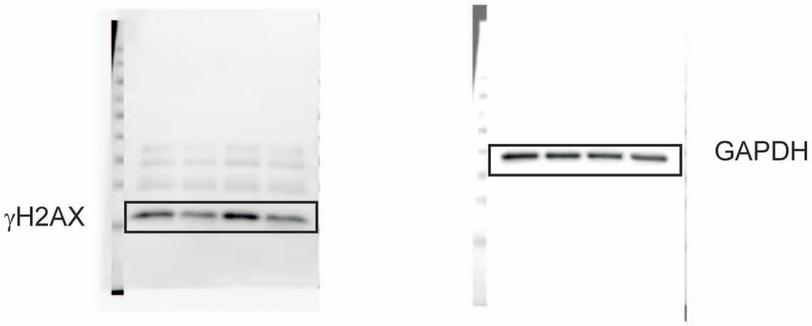

Suppl. Fig S6c

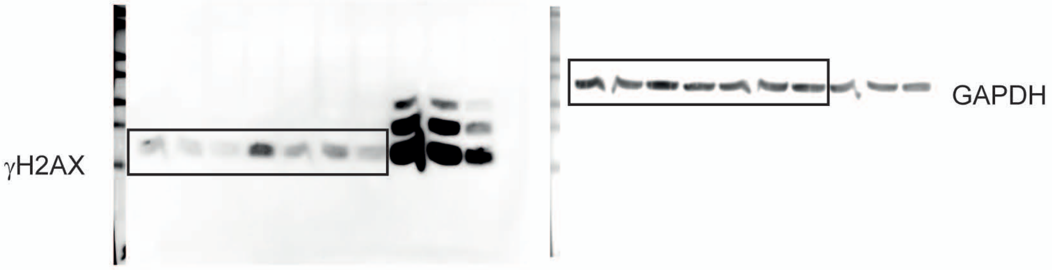

Suppl. Fig S6d

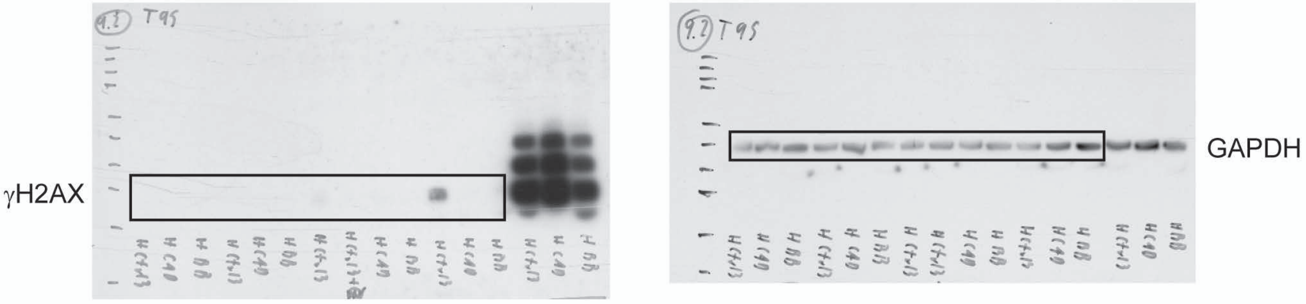

Suppl. Fig S7a

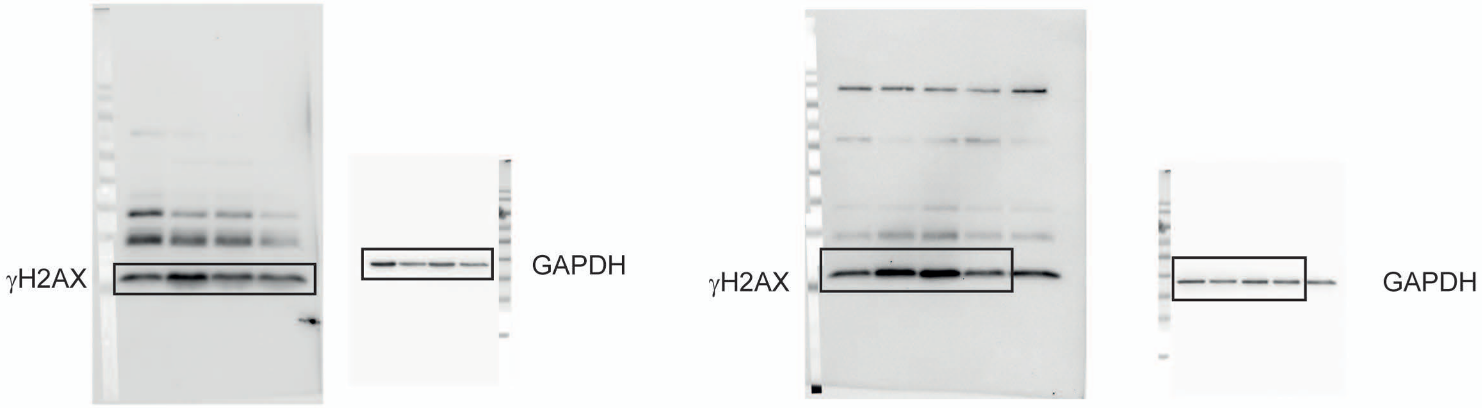

Suppl. Fig S7c

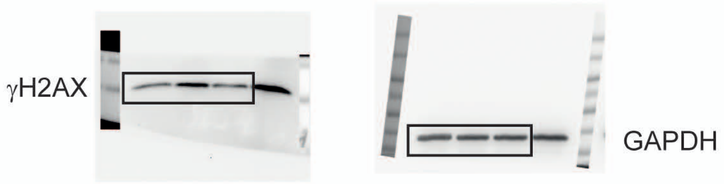

Suppl. Fig S7d

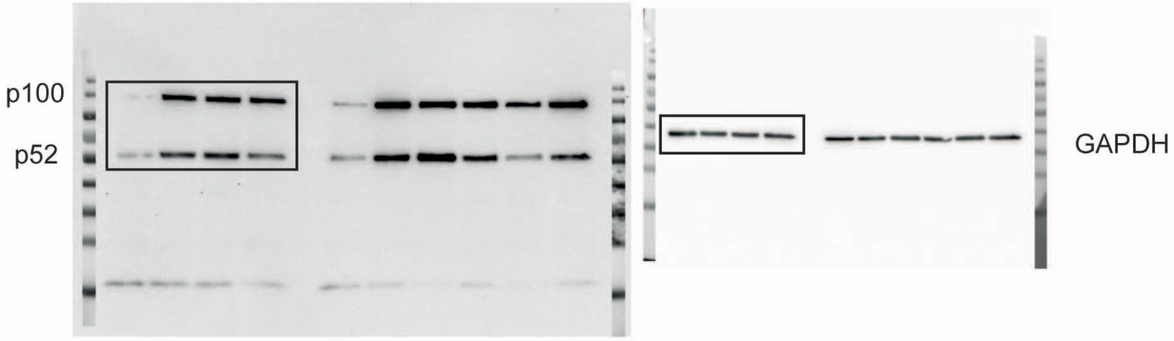

Suppl. Fig S7f

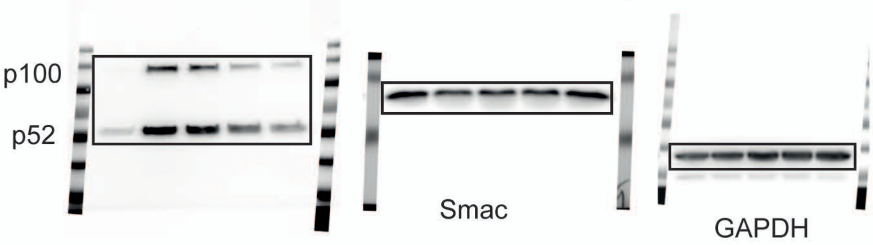

Suppl. Fig S7g

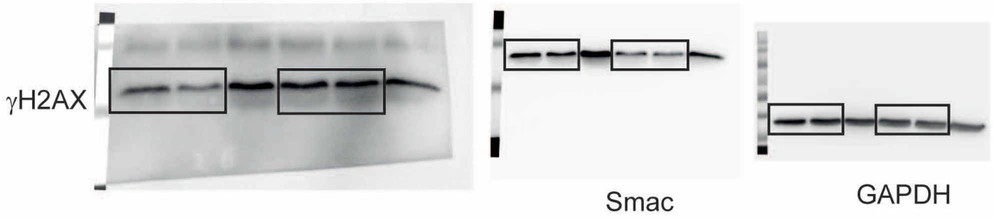

Suppl. Fig S7h

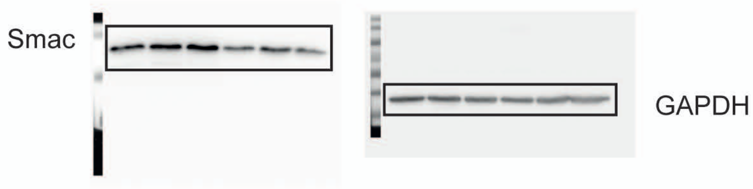

Supplement: Supplementary file 13 — Supplementary File 1 [file 41418_2022_1009_MOESM13_ESM.pdf]
