# Supplementary material for: Mitochondria supply sub-lethal signals for cytokine secretion and DNA-damage in H. pylori infection
Source: Cell Death Differ. 2022 May 3;29(11):2218–32. doi: 10.1038/s41418-022-01009-9 (PMC9613881; doi:10.1038/s41418-022-01009-9)
Supplement: Supplementary file 15 — Pre-authorship form [file 41418_2022_1009_MOESM15_ESM.pdf]

SPRINGER NATURE

Change of authorship request form - Journals (pre-acceptance)

Section 6: Declaration of agreement. *All* authors, unchanged, new and removed *must* sign this declaration.
~~(NB: Please print the form, (docu) sign and return/~~upload a scanned copy. ~~Please note that signatures that have been inserted as an image file are acceptable as long as it is handwritten. Typed names in the signature box are unacceptable.)~~ * Please delete as appropriate. Delete all of the bold if you were on the original authorship list and are remaining as an author.

	First name	Family name		Signature	Date
1st author	Benedikt	Doerflinger	I agree to the proposed new authorship shown in section 4 /~~and the addition/removal*of my name to the authorship list~~ /and the proposed change in corresponding author	[illegible]	15.03.22
2nd author	M. Tarek	Badr	I agree to the proposed new authorship shown in section 4 /~~and the addition/removal*of my name to the authorship list~~ /and the proposed change in corresponding author	Mohamed Badr	14-04.22
3rd author	Aladin	Haimovici	I agree to the proposed new authorship shown in section 4 /~~and the addition/removal*of my name to the authorship~~ list /and the proposed change in corresponding author	[illegible]	16.3.22
4th authors	Lena	Fischer	I agree to the proposed new authorship shown in section 4 /~~and the addition/removal*of my name to the authorship list~~ /and the proposed change in corresponding author	Lena Fischer	13.04.22
5th author	JULIANE	VIER	I agree to the proposed new authorship shown in section 4 /~~and the addition/removal*of my name to the authorship list~~ /and the proposed change in corresponding author	Juliane Vier	11.3.22
6th author	Arlena	Metz	I agree to the proposed new authorship shown in section 4 /~~and the addition/removal*of my name to the authorship list~~ /and the proposed change in corresponding author	A. Metz	14.4.22
7th author	Bianca	Eisele	I agree to the proposed new authorship shown in section 4 /~~and the addition/removal*of my name to the authorship~~ list /and the proposed change in corresponding author	B. Eisele	16.3.22

SPRINGER NATURE Springer Nature is one of the world's leading global research, educational and professional publishers, created in May 2015 through the combination of Nature Publishing Group, Palgrave Macmillan, Macmillan Education and Springer Science+Business Media.

SPRINGER NATURE

Change of authorship request form - Journals (pre-acceptance)

	First name	Family name		Signature	Date
8th author	Peter	Bronsert	I agree to the proposed new authorship shown in section 4 /~~**and the addition/removal*of my name to the authorship list**~~ /and the proposed change in corresponding author	[illegible]	15.09.22
9th author	Konrad	Aumann	I agree to the proposed new authorship shown in section 4 /**and the addition/removal*of my name to the authorship list** /and the proposed change in corresponding author		
10th author	Jens	Höppner	I agree to the proposed new authorship shown in section 4 /**and the addition/removal*of my name to the authorship list** /and the proposed change in corresponding author		

Please use an additional sheet if there are more than 10 authors.

In case of author collaborations with formal agreement:

	Name of consortium/consortia	First name	Family name		Signature	Date
Representative/legal guarantor				I agree to the proposed new authorship shown in section 4 /**and the addition/removal*of my name to the authorship list** /and the proposed change in corresponding author		

Both added/removed authors should complete the information in the first table under Section 6.

---- End of form ----

SPRINGER NATURE Springer Nature is one of the world's leading global research, educational and professional publishers, created in May 2015 through the combination of Nature Publishing Group, Palgrave Macmillan, Macmillan Education and Springer Science+Business Media.

SPRINGER NATURE

Change of authorship request form - Journals (pre-acceptance)

	First name	Family name		Signature	Date
8th author	Peter	Bronsert	I agree to the proposed new authorship shown in section 4 /and the **addition/removal*of my name to the authorship list** /and the proposed change in corresponding author		
9th author	Konrad	Aumann	I agree to the proposed new authorship shown in section 4 /~~and the addition/removal*of my name to the authorship list~~ /and the proposed change in corresponding author	[signature]	19.4.22
10th author	Jens	Höppner	I agree to the proposed new authorship shown in section 4 /and the **addition/removal*of my name to the authorship list** /and the proposed change in corresponding author		

Please use an additional sheet if there are more than 10 authors.

In case of author collaborations with formal agreement:

	Name of consortium/consortia	First name	Family name		Signature	Date
Representative/legal guarantor				I agree to the proposed new authorship shown in section 4 **/and the addition/removal*of my name to the authorship list** /and the proposed change in corresponding author		

Both added/removed authors should complete the information in the first table under Section 6.

---- End of form ----

Change of authorship request form - Journals (pre-acceptance)

	First name	Family name		Signature	Date
8th author	Peter	Bronsert	I agree to the proposed new authorship shown in section 4 /**and the addition/removal*of my name to the authorship list** /and the proposed change in corresponding author		
9th author	Konrad	Aumann	I agree to the proposed new authorship shown in section 4 /**and the addition/removal*of my name to the authorship list** /and the proposed change in corresponding author		
10th author	Jens	Höppner	I agree to the proposed new authorship shown in section 4 /~~**and the addition/removal*of my name to the authorship list**~~ /and the proposed change in corresponding author	[illegible]	14.4.22

Please use an additional sheet if there are more than 10 authors.

In case of author collaborations with formal agreement:

	Name of consortium/consortia	First name	Family name		Signature	Date
Representative/legal guarantor				I agree to the proposed new authorship shown in section 4 /**and the addition/removal*of my name to the authorship list** /and the proposed change in corresponding author		

Both added/removed authors should complete the information in the first table under Section 6.

---- End of form ----

SPRINGER NATURE

Change of authorship request form - Journals (pre-acceptance)

Section 6: Declaration of agreement. *All* authors, unchanged, new and removed *must* sign this declaration.
(NB: Please print the form, (docu)-sign and return/upload a scanned copy. Please note that signatures that have been inserted as an image file are acceptable as long as it is handwritten. Typed names in the signature box are unacceptable.) *** Please delete as appropriate. Delete all of the bold if you were on the original authorship list and are remaining as an author.**

	First name	Family name		Signature	Date
11st author	Collins	Waguia Kontchou	I agree to the proposed new authorship shown in section 4 /~~**and the addition/removal*of my name to the authorship list**~~ /and the proposed change in corresponding author	[illegible]	16.03.22
12nd author	Ishita	Parui	I agree to the proposed new authorship shown in section 4 /~~**and the addition/removal*of my name to the authorship list**~~ /and the proposed change in corresponding author	Ishita Parui	17.03.22
13rd author	Arnim	Weber	I agree to the proposed new authorship shown in section 4 /~~**and the addition/removal*of my name to the authorship list**~~ /and the proposed change in corresponding author	Arnim Weber	17.3.22
14th authors	SUSANNE	KIRSCHNEK	I agree to the proposed new authorship shown in section 4 /~~**and the addition/removal*of my name to the authorship list**~~ /and the proposed change in corresponding author	[illegible]	11.3.22
15th author	Georg	Häcker	I agree to the proposed new authorship shown in section 4 /~~**and the addition/removal*of my name to the authorship list**~~ /and the proposed change in corresponding author	[illegible]	21.3.22
6th author			I agree to the proposed new authorship shown in section 4 **/and the addition/removal*of my name to the authorship list** /and the proposed change in corresponding author		
7th author			I agree to the proposed new authorship shown in section 4 **/and the addition/removal*of my name to the authorship list** /and the proposed change in corresponding author		

SPRINGER NATURE Springer Nature is one of the world's leading global research, educational and professional publishers, created in May 2015 through the combination of Nature Publishing Group, Palgrave Macmillan, Macmillan Education and Springer Science+Business Media.
